# The cost of not breastfeeding: global results from a new tool

**DOI:** 10.1093/heapol/czz050

**Published:** 2019-06-24

**Authors:** Dylan D Walters, Linh T H Phan, Roger Mathisen

**Affiliations:** 1 Nutrition International, 180 Elgin St, Ottawa, ON, Canada; 2 Alive & Thrive, 60 Ly Thai To Street, Hoan Kiem, Ha Noi, Viet Nam

**Keywords:** Breastfeeding, nutrition, maternal and child health, economic evaluation

## Abstract

Evidence shows that breastfeeding has many health, human capital and future economic benefits for young children, their mothers and countries. The new *Cost of Not Breastfeeding tool*, based on open access data, was developed to help policy-makers and advocates have information on the estimated human and economic costs of not breastfeeding at the country, regional and global levels. The results of the analysis using the tool show that 595 379 childhood deaths (6 to 59 months) from diarrhoea and pneumonia each year can be attributed to not breastfeeding according to global recommendations from WHO and UNICEF. It also estimates that 974 956 cases of childhood obesity can be attributed to not breastfeeding according to recommendations each year. For women, breastfeeding is estimated to have the potential to prevent 98 243 deaths from breast and ovarian cancers as well as type II diabetes each year. This level of avoidable morbidity and mortality translates into global health system treatment costs of US$1.1 billion annually. The economic losses of premature child and women’s mortality are estimated to equal US$53.7 billion in future lost earnings each year. The largest component of economic losses, however, is the cognitive losses, which are estimated to equal US$285.4 billion annually. Aggregating these costs, the total global economic losses are estimated to be US$341.3 billion, or 0.70% of global gross national income. While the aim of the tool is to capture the majority of the costs, the estimates are likely to be conservative since economic costs of increased household caregiving time (mainly borne by women), and treatment costs related to other diseases attributable to not breastfeeding according to recommendations are not included in the analysis. This study illustrates the substantial costs of not breastfeeding, and potential economic benefits that could be generated by government and development partners’ investments in scaling up effective breastfeeding promotion and support strategies.

**Key Messages**

- The new *Cost of Not Breastfeeding Tool* is a first-of-its-kind resource to help policy-makers and advocates quantify the human and economic costs of not breastfeeding including lost life, lost productivity, and increased costs to health systems at country, regional and global levels.
- Globally, 595 379 childhood deaths are attributed to not breastfeeding annually. Optimal breastfeeding also has the potential to prevent an additional 98 243 deaths of mothers from cancers and type II diabetes each year.
- The total annual global economic losses are estimated to be between US$257 billion and US$341 billion, or between 0.37% and 0.70% of global gross national income.
- The costs of not breastfeeding are significant and should compel policy-makers and donors to invest in scaling up breastfeeding and nutrition interventions for children and their mothers to strengthen human capital development and economic outcomes around the world.

## Introduction

Newborns and young children receive essential benefits through breastfeeding resulting in improved survival, health and human capital outcomes. Breastfeeding reduces the risk of childhood infections such as diarrhoea and pneumonia and premature mortality as well as minimizes nutrition-related harm to cognitive development in early childhood (Horta *et al.*, 2015; Victora *et al.*, 2016). For breastfeeding mothers, breastfeeding reduces the risk of post-partum haemorrhage and depression as well as premature mortality from several diseases later in life (Chowdhury *et al.*, 2015; Victora *et al.*, 2016). For these reasons, the World Health Organization (WHO) and UNICEF recommend early initiation of breastfeeding within an hour of birth, exclusive breastfeeding of infants for the first 6 months of life and continued breastfeeding with complementary foods for two or more years (UNICEF, 2016a).

Despite the substantial evidence on the health and cognitive benefits of breastfeeding, the vast majority of children globally are not breastfed in line with the recommendations. The global prevalence of exclusive breastfeeding increased from 36% in 2000 to 43% in 2015, and the current prevalence of early initiation of breastfeeding and continued breastfeeding until 2 years of age are 45% and 46%, respectively (UNICEF, 2016a). The Lancet Series on Breastfeeding estimated that >800 000 child deaths globally and cognitive losses totalling US$302 billion per year were attributable to not breastfeeding according to recommendation and exposure to breastmilk substitutes (Rollins et al., 2016; Victora *et al.*, 2016).

The current pace of increase in the prevalence of exclusive breastfeeding is insufficient for achieving the World Health Assembly’s (WHA) Global Nutrition Target of ‘increasing the rate of exclusive breastfeeding in the first six months up to at least 50%’ by 2025 (WHO and UNICEF, 2014). While the cultural, social, economic and corporate forces that shape breastfeeding in different regions around the world may be challenging to counter, there is evidence that policy and programmatic actions by governments, donors and civil society can effectively increase the prevalence of breastfeeding practices (Rollins et al., 2016). The World Bank’s Investment Framework for Nutrition estimated that US$5.7 billion in additional financing is needed from 2016 to 2025 to scale up breastfeeding promotion interventions across low- and middle-income countries (LMICs) to achieve the WHA target for breastfeeding (Shekar *et al.*, 2017; Walters *et al.*, 2017). Any new resources mobilized for increasing breastfeeding should be invested towards fully implementing and enforcing the International Code of Marketing of Breastmilk Substitutes, enacting paid family leave and workplace breastfeeding policies, implementing the Ten Steps to Successful Breastfeeding in facilities providing maternity and newborn services (WHO, 2017) and improving access to skilled breastfeeding counselling.

Previous studies have quantified the economic costs of not breastfeeding, or malnutrition, at the global level (Holla *et al.*, 2015; Rollins et al., 201641), regional level (Horton *et al.*, 1996; Walters *et al.*, 2016) and national or sub-national levels in some countries (Smith *et al.*, 2002; Büchner *et al.*, 2007; Bagriansky and Voladet, 2013; Council for Agriculture and Rural Development *et al.*, 2013; Bagriansky, 2014; Bartick *et al.*, 2017). The recent nutrition investments of US$500 million in Nigeria in 2016 and US$4.5 billion in Indonesia in 2017 nutrition are examples where the use of economic research by advocacy groups helped to raise policy-maker awareness that led to the prioritization and investments in nutrition programmes. Unfortunately, there remain gaps in access to evidence on the human capital and economic consequences of malnutrition or not breastfeeding by policy-makers and advocates in the majority of the world’s countries.

To address this gap in economic research, the *Cost of Not Breastfeeding Tool* was envisioned to provide an evidence-based and user-friendly tool for policy-makers and advocates to generate accurate estimates of the human and economic consequences of not breastfeeding and exposure to breastmilk substitutes in their countries on a consistent basis. Alive & Thrive, an initiative managed by FHI 360 and funded by the Bill and Melinda Gates Foundation that is dedicated to saving and improving lives through optimal maternal nutrition, breastfeeding and complementary feeding practices, supported the development of the tool. The objective of this article is to present the underpinning methodology of the new *Cost of Not Breastfeeding Tool* as well as new findings on the cost of not breastfeeding at the country, regional and global levels.

## Methods

**Figure 1:**
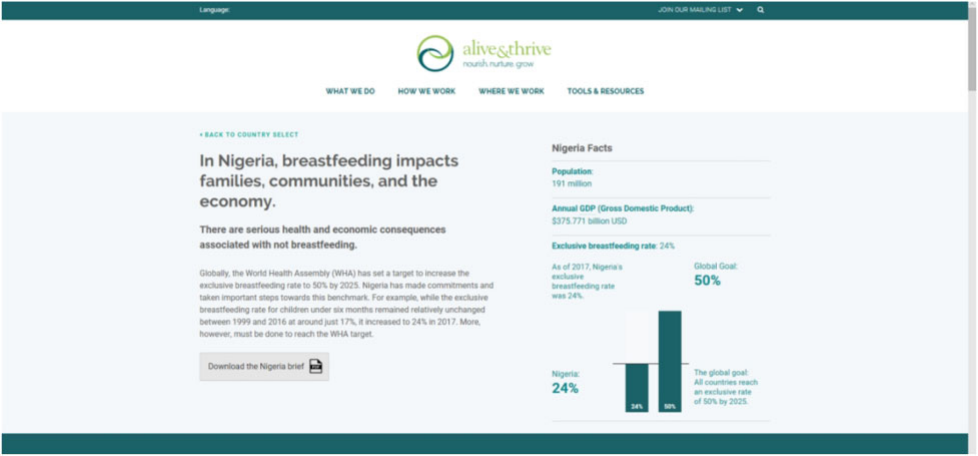
The online *Cost of Not Breastfeeding Tool*.

This *Cost of Not Breastfeeding Tool* was developed in Microsoft Excel, and is now available as both an interactive online tool (Figure 1) and a downloadable workbook file.^1^ The user-friendly online tool summarizes the results of the national estimates for the human consequences (including morbidity and mortality) and economic costs for select LMICs. The tool provides users with data and narrative advocacy briefs that summarize the results. The downloadable workbook version, intended for advanced technical users, consists of worksheets containing open access datasets, the calculations for each indicator, and the results of the global, regional and national analysis for the human and economic costs for over 130 countries. The workbook version includes a macro programme that computes the regional and global estimates based on two user inputs (i.e. discount rate and long-term Gross Domestic Product (GDP) growth rate). This section describes the analytical methods and data sources used for each of the included indicators of the cost of not breastfeeding.

### Analytical methods

The analytical methods used in the *Cost of Not Breastfeeding Tool* were drawn from previously published studies on the economic consequences of malnutrition (Bagriansky and Voladet, 2013; Bagriansky, 2014), cost of not breastfeeding (Horton *et al.*, 1996; Drane, 1997; Smith *et al.*, 2002; Büchner *et al.*, 2007; Bartick and Reinhold, 2010; Bartick *et al.*, 2013; Pokhrel *et al.*, 2014; Walters *et al.*, 2016; Bartick *et al.*, 2017), the Lancet Series on Breastfeeding (Rollins et al., 2016; Victora *et al.*, 2016) and the Investment Framework for Nutrition (Shekar *et al.*, 2017; Walters *et al.*, 2017). Some modifications to the methods were necessary to be compatible with the variables contained in open access datasets.

The cost of not breastfeeding tool incorporates three categories of indicators for human and economic costs attributed to not breastfeeding according to recommendations, including (1) women’s and child morbidity and mortality, (2) for health system and household formula costs and (3) the future economic costs due to mortality and cognitive losses. This study was based on the framework for estimating economic costs of not breastfeeding from the Rollins *et al.* (2016) Lancet Series on Breastfeeding, which focused on cognitive losses and healthcare treatment costs for children. While this analysis was conducted from the societal perspective with certain costs borne by households, ministries of health and the economy as a whole included, the tool design focused on the key costs with available data for the majority of countries rather than the exhaustive inclusion of all type of costs. The estimates are likely to be conservative since the economic costs of increased household caregiving time, which are mainly borne by women (Leslie, 1989), transportation costs, and several child and maternal diseases that can be prevented in part by breastfeeding are not included in the model due to the data availability. Each indicator was estimated for a 1-year cohort of newborns and infants not being breastfed according to recommendations or their mothers. The future economic costs of mortality and cognitive losses for this 1-year cohort of child–mother pairs, however, are projected into the long-term future over their productive years.

#### Child and maternal morbidity and mortality

The estimation of the human cost of not breastfeeding follows the same methodology from previous studies on the cost of malnutrition and not breastfeeding (Bagriansky and Voladet, 2013; Bagriansky, 2014; Walters *et al.*, 2016). To estimate the number of childhood cases and child deaths averted each year attributable to not breastfeeding according to recommendation, published relative risks for either the diarrhoea or pneumonia infection pathway (Black *et al.*, 2008) were multiplied by the current percentage of households in each breastfeeding behaviour category, and then multiplied by the morbidity or mortality for each disease for infants and young children (age 0–23 months). The method used for estimating the morbidity and mortality of breast and ovarian cancer and type II diabetes attributed with not breastfeeding follows previous approaches used in the UK (Pokhrel *et al.*, 2014) and for global estimates (Victora *et al.*, 2016). This calculation is based on the fact that lifetime duration of breastfeeding a child reduces the relative risk of morbidity from cancer and it is assumed that the effect on mortality is of the same magnitude. The number of potential cases averted with full breastfeeding rates (100%) was calculated by multiplying the incidence of type II diabetes in women (Institute for Health Metrics and Evaluation, 2017) by published relative risk of type II diabetes (Victora *et al.*, 2016) and the current level of breastfeeding in each country. Data on breast and ovarian cancer mortality in women by country are from *GLOBOCAN 2012* (International Agency for Research on Cancer, 2012). The tool does not include the attributed morbidity and mortality consequences from childhood diabetes and cancer, sudden infant death syndrome, and necrotizing enterocolitis. All regional and country income group estimates of morbidity and mortality are based on the cumulative totals from countries with available data and are not extrapolated to fit the total population of the respective group.

#### Health system and household formula costs

The health system cost refers specifically to the direct medical costs for the treatment of cases of childhood diarrhoea and pneumonia (Bagriansky and Voladet, 2013; Bagriansky, 2014; Walters *et al.*, 2016) and cases of type II diabetes in women that can be attributed to not breastfeeding. The number of cases of each childhood disease attributed to not breastfeeding was multiplied by the percentage of children with the disease taken to a health facility, the percentage of cases taken to a health facility that receive either outpatient care services or inpatient services (Lamberti *et al.*, 2012; Niederman and Krilov, 2013) and the percentage of patients that seek care at each level of care (health centre, primary hospital, secondary hospital or teaching/tertiary hospital) in each country. The annual total cost of treatment of diarrhoea and pneumonia attributed to not breastfeeding is equal to the number of cases that receive outpatient and inpatient services at each level of care multiplied by the unit cost of treatment for children at each level of care.

The default country-level unit cost data used for the estimation of the treatment of childhood diseases was from the WHO-CHOICE data (Johns *et al.*, 2005), which were adjusted for the cumulative inflation based on country-specific data from 2008 to 2017 in US dollars (World Bank, 2016). These unit costs pertain crudely to the mean cost of general outpatient services per visit and inpatient care per day. Additional data on the unit costs of treatment for childhood diarrhoea and pneumonia were collected from China, Ethiopia, Ghana, Mexico and Nigeria by UNICEF and Alive & Thrive country teams, consultants and partners, either from national administrative databases or directly from health facilities (see Supplementary Appendix Table A1).

The estimated health system cost of treatment for type II diabetes in women is calculated by multiplying the number of annual cases of type II diabetes attributed to not breastfeeding by an estimate on the percentage of cases of type II diabetes that are diagnosed. This figure represents the number of cases that are diagnosed and potentially treated, which is then multiplied by the health expenditure per case of type II diabetes in each country from the International Diabetes Federation Diabetes Atlas (International Diabetes Federation, 2015).

The cost of feeding a young child with formula from birth until age two as a percentage of household earnings was calculated by multiplying the total estimated quantity of formula advised by breastmilk substitute producers and manufacturers by the unit cost of formula, then divided by the nominal wage or mean earnings of employees in each country. Data on the unit price of infant formula from 97 countries was collected for this study through an online search for e-commerce vendors based in each country. The lowest price of an economy brand of infant formula was selected for this analysis to be conservative since the average price paid is unknown (see Supplementary Appendix Table A2 for unit costs). For countries where data on the unit cost of formula was not available, the mean global unit cost of formula was used as a proxy.

#### Future economic cost of mortality and cognitive losses

The estimation of potential future cost of child and maternal mortality, or future income lost due to premature mortality attributed to not breastfeeding, follows the *WHO-CHOICE* methodology (Johns *et al.*, 2005) and the approach in the *Investment Framework for Nutrition* (Shekar *et al.*, 2017). This cost reflects the potential contribution to a country’s economy through future earnings over a person’s productive years that will be lost with premature mortality attributed to not breastfeeding. The total potential future income lost due to child mortality is equal to the sum of multiplying the number of child deaths attributed to not breastfeeding according to recommendation for the 1-year cohort by each country’s projected gross national income (GNI) per capita (World Bank, 2016) from the year the children turn 18 years of age until the earliest point between the expected retirement at age 65 or the country’s age of life expectancy (World Bank, 2016). The calculation of total potential future income lost due to maternal mortality is similar; however, the economic losses are only counted for the foregone productive years between the mean age of women’s cancer and diabetes-related mortality until the earliest point between the expected retirement at age 65 or the country’s age of life expectancy.

The estimation of potential future income not realized due to child cognitive losses attributed to not breastfeeding follows a methodology used previously (Rollins et al., 2016; Walters *et al.*, 2016; Walters *et al.*, 2017). This cost reflects the potential contribution to a country’s economy through increased earnings over a person’s productive years that will be lost due to not achieving cognitive gains in intelligence provided by being breastfed according to recommendations in the early years of childhood. This analysis calculated the cognitive losses by assuming that exclusive breastfeeding below 6 months of age compared with non-exclusive breastfeeding can achieve the same cognitive gains equal to a 2.62 IQ increase compared with not being breastfed (Horta *et al.*, 2015; Victora *et al.*, 2016). The 2.62 IQ increase point estimate was adjusted for maternal IQ and used in the tool in order to be conservative (Horta *et al.*, 2015). This approach was supported by evidence from a study that found that children exclusively breastfed for greater than 1 month experienced an increase in three IQ points compared with children not or partially breastfed for less than 1 month (Eickmann *et al.*, 2007). Although there is only limited research on the direct effects of exclusive breastfeeding due to challenges with feasibility, the approach aligns with UNICEF and WHO guidelines and data in the infant and young child database (UNICEF, 2016a; UNICEF, 2017).

The potential future income lost due to cognitive losses is equal to multiplying the number of children not breastfed by GNI per capita (World Bank, 2016), the 2.62 IQ point increase lost per child not breastfed (Horta *et al.*, 2015; Victora *et al.*, 2016) and 1.067% increase in earnings lost for each IQ point lost (Hanushek and Woessmann, 2008). The total future income lost in a country is equal to the sum of this calculation for each year from the point that the children would have turned 18 years of age until expected retirement at age 65 or the country’s age of life expectancy, whichever comes first (World Bank, 2016).

The total combined economic losses of not breastfeeding is equal to the sum of the health system cost, future economic cost of mortality and future economic cost of cognitive losses for each country, region or the globe.^2^ This is also presented as a percent share of GNI (World Bank, 2016). The results for economic losses presented in this article all assume a 3% discount rate on potential future economic losses of not breastfeeding as recommended in the Bill and Melinda Gates Foundation *Reference Case for Economic Evaluations* (Bill and Melinda Gates Foundation, 2014), *WHO-CHOICE* methodology (Johns *et al.*, 2005) and the *Investment Framework for Nutrition* (Shekar *et al.*, 2017). The calculations assumed a long-term mean annual GDP growth rate of 3% in each country (Shekar *et al.*, 2017) and the wage share of national income in each country from International Labour Organization (ILO) STAT database (Lubker, 2007; ILO, 2015; Shekar *et al.*, 2017).

Sensitivity analysis can help to describe the nuances of estimates of economic losses based on changes to key variables with uncertainty.^3^ Sensitivity analysis on the economic losses is presented using both conservative assumptions of 5% discount rates on benefits and 1.5% mean long-term GDP growth rate as well as optimistic assumptions using a 1.5% discount rate and 5% mean long-term GDP growth rate.^4^ The estimated economic losses for both sets of conservative and optimistic assumptions are presented in parentheses next to the result using default assumptions in Table 4 for regions and country income groups Supplementary Appendix Tables A6–8 for all individual countries with data available. In addition, estimates for economic losses are also presented using an alternative approach for calculating children’s cognitive losses, which assumes that breastfeeding at 6 months is associated with a 2.62 point IQ increase compared with not being breastfed^5^ (Supplementary Appendix Tables A6–8). A limitation of the sensitivity analysis in the tool is that it does not account for variation in the effects of not breastfeeding on disease morbidity, mortality and cognition.

### Data sources

Data sources used across the indicators for each country include: (1) the United Nations World Population Prospects for population data by age group (United Nations Department of Economic and Social Affairs, 2015); (2) the UNICEF Infant and Young Child Feeding Database (UNICEF, 2017), data from Lancet Series on Breastfeeding Supplementary Appendix, Demographic Health Survey (DHS) STAT compiler (Demographic and Health Surveys Program, 2017), or Multiple-Indicator Cluster Surveys (MICS) compiler (UNICEF, 2016b) for breastfeeding practice prevalence; (3) the Global Burden of Disease results tool (Institute for Health Metrics and Evaluation, 2017) or WHO Global Health Observatory data (WHO, 2015) for the total mortality by disease by age group and gender; (4) the Global Burden of Disease Results Tool (Institute for Health Metrics and Evaluation, 2017) for disease incidence by age group and gender; (5) MICS compiler (UNICEF, 2016b) for data on care seeking behaviour for childhood diarrhoea and pneumonia; (6) the World Bank’s World Development Indicators Database (World Bank, 2016) for other economic and health indicators such as country-level GNI, GNI per capita and life expectancy; and (7) the International Labour Office’s ILOSTAT database (ILO, 2015) for the labour force participation rate, wage share of income and mean earnings.

## Results

### Global and regional results

#### Child morbidity and mortality

**Figure 2:**
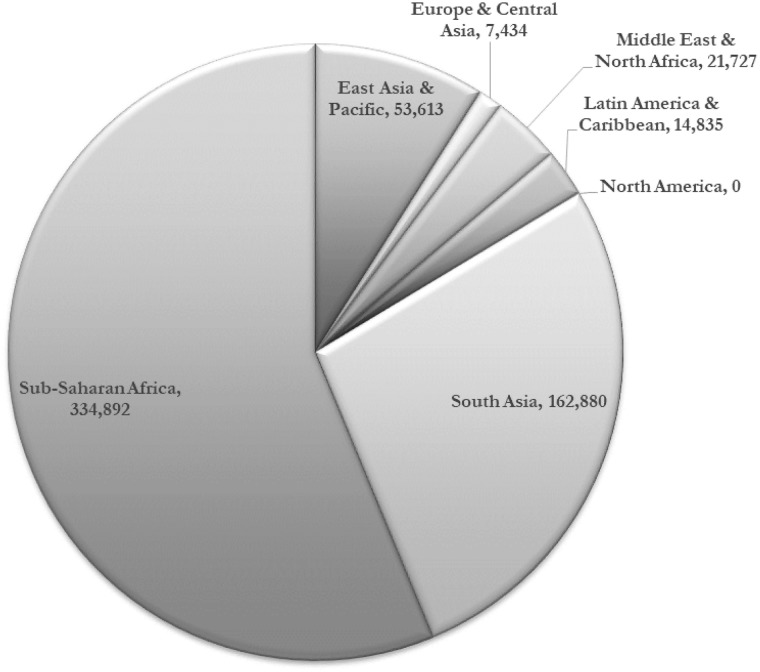
Number of child deaths attributed to not breastfeeding by region.

Based on epidemiological and demographic health surveillance data available for 130 countries, not breastfeeding according to recommendations was attributed with 166 million and 9 million avoidable cases of diarrhoea and pneumonia in children under the age of two each year (Table 1). Approximately two-thirds of these cases of infectious illnesses occur in the South Asia and Sub-Saharan Africa regions and 84% within countries in the lower middle-income and low-income groups (Table 1). This high level of avoidable morbidity leads to substantial preventable child mortality. Breastfeeding was attributed with 595 379 child deaths to diarrhoea (38%) and pneumonia (62%) each year (Table 2). Of the global total of child mortality, over 56% occurs in Sub-Saharan Africa and 64% occur in lower middle-income countries (Table 2 and Figure 2). An estimated 974 956 cases of childhood obesity each year were attributed to not breastfeeding according to recommendations. Over 40% of the new preventable childhood obesity cases were in East Asia and the Pacific region and 54% in upper middle-income countries (Table 1), both groupings with rapid growth in commercial milk-based formula sales in recent years (Baker *et al.*, 2016).

**Table 1:** Number of annual cases of morbidity in women and children attributable to not breastfeeding by region and country income group

Number of cases of morbidity attributable to not breastfeeding						
Regions	Child diarrhoea (0–23 months)	Child ARI/ pneumonia (0–23)	Childhood obesity	Breast cancer in mothers	Ovarian Cancer in mothers	Type II diabetes
East Asia and Pacific	19 533 482	1 058 553	374 405	54 393	9 905	389 006
Europe and Central Asia	3 514 920	357 835	69 231	11 016	3 214	52 077
Middle East and North Africa	12 704 537	716 047	168 568	8379	902	101 441
Latin America and Caribbean	13 160 409	778 792	134 192	18 254	3 355	118 363
North America	33 571	64 739	0	0	0	0
South Asia	56 056 468	3 346 020	74 249	14 051	2435	121 620
Sub-Saharan Africa	60 843 179	2 317 553	154 311	11 711	1745	75 009
High income	335 534	86 075	9362	3249	728	15 719
Upper-middle-income	24 816 631	1 706 300	528 011	68 921	12 696	494 165
Lower-middle-income	99 358 380	5 375 448	356 162	39 297	7230	313 822
Low income	41 336 021	1 471 716	81 421	6337	902	33 809
Total	165 846 566	8 639 539	974 956	117 804	21 556	857 515

#### Mother’s morbidity and mortality

Breastfeeding was estimated to have the potential to prevent 27 069 future deaths of women from breast cancer and 13 644 from ovarian cancer each year with universal breastfeeding (Table 2). Also, breastfeeding could potentially prevent 58 230 deaths of women from type II diabetes. In contrast with child mortality, the majority of preventable maternal deaths due to not breastfeeding, 53% occur in upper middle-income countries and 38% occur in the East Asia and the Pacific region alone (Table 2). Results for the maternal and child morbidity and mortality indicators from 130 individual countries with data available can be found in Supplementary Appendix Tables A3 and A4.

**Table 2:** Number of annual deaths of women and children attributable to not breastfeeding by region and country income group

Number of deaths attributable to not breastfeeding							
Regions	Due to child diarrhoea (0–23 months)	Due to child ARI/pneumonia (0–23)	Total child deaths	Due to breast cancer in mothers	Due to ovarian cancer in mothers	Due to Type II diabetes in mothers	Total number of maternal deaths
East Asia and Pacific	13 932	39 680	53 613	11 898	5922	19 964	37 785
Europe and Central Asia	2132	5302	7434	3007	1877	2683	7567
Middle East and North Africa	6455	15 272	21 727	1801	606	5261	7668
Latin America and Caribbean	3938	10 897	14 835	4292	2092	11 503	17 887
North America	0	0	0	0	0	0	0
South Asia	66 530	96 350	162 880	3444	1677	10 791	15 913
Sub-Saharan Africa	132 828	202 064	334 892	2626	1471	8028	12 125
High income	11	95	106	704	471	654	1829
Upper-middle-income	10 928	33 952	44 879	15 677	7619	29 414	52 711
Lower-middle-income	147 999	233 025	381 024	9313	4763	25 323	39 399
Low income	66 877	102 493	169 370	1374	791	2839	5004
Total	225 815	369 565	595 379	27 069	13 644	58 230	98 943

#### Health system and household formula costs

The total global health system cost, based on the tool design, for the treatment of childhood diarrhoea and pneumonia as well as women’s type II diabetes that could be prevented with breastfeeding is estimated to be US$1.1 billion annually (Table 3). The treatment costs for childhood pneumonia alone equal US$697 million each year, and childhood diarrhoea and women’s type II diabetes are estimated to cost US$196 million and US$254 million, respectively. For all three diseases, East Asia and the Pacific would incur the highest cost of any region with a total of US$315 million per year. The upper middle-income group of countries would incur the highest health system cost at US$604 million per year. Supplementary Appendix Table A3 shows the total annual health system treatment cost from not breastfeeding according to recommendation for >138 individual countries with data available.

**Table 3:** Total health system cost attributed to not breastfeeding by region and country income group

Cost of avoidable healthcare treatment due to not breastfeeding				
Regions	Due to childhood diarrhoea (US$ m)	Due to childhood pneumonia (US$ m)	Due to type II diabetes morbidity in mothers' (US$ m)	Total (US$ m)
East Asia and Pacific	78.40	182.05	54.60	315.04
Europe and Central Asia	27.16	38.81	29.86	95.84
Middle East and North Africa	19.88	81.51	30.69	132.08
Latin America and Caribbean	48.30	128.67	59.99	236.96
North America	0.00	1.83	26.44	28.27
South Asia	5.43	128.38	33.59	167.41
Sub-Saharan Africa	17.02	135.44	18.76	171.21
High income	20.76	16.83	33.86	71.45
Upper-middle-income	149.10	308.56	146.79	604.46
Lower-middle-income	25.16	335.49	69.38	430.03
Low income	1.17	35.80	3.91	40.88
Total	196.19	696.69	253.94	1146.81

The online search for e-commerce vendors in all countries found data on infant formula in 97 countries (Supplementary Appendix Table A2). The global mean price per 900-g container of the lowest price of an economy brand was found to be US$18.74. With this country-level unit cost data on infant formula, it was estimated that feeding a child with an economy brand of formula for the first 2 years of life instead of breastfeeding would cost on average over 6.1% of a household’s wages. This figure would be even higher in low-income families and LMICs (Supplementary Appendix Table A5).

#### Economic costs of mortality and cognitive losses

In 125 countries with data available, the economic losses due to child mortality, which are the future earnings not generated by over a half-million children who die prematurely each year due to not breastfeeding according to recommendations, equal US$53.7 billion annually (Table 4). Over US$23.6 billion, or 43% of the total losses calculated, would be lost in the Sub-Saharan African region, and another US$10.6 and US$10.4 billion would be lost in each of South Asia and East Asia and the Pacific regions, respectively. The economic losses due to maternal mortality, which are the future earnings not generated by 98 943 mothers who will die prematurely, is estimated to equal US$1.26 billion annually (Table 4).

**Table 4:** Global and regional economic losses due to mortality, cognitive losses and total attributable to not breastfeeding by region and by country income group

Economic losses attributable to not breastfeeding					
	Due to child mortality (US$ b)	Due to maternal mortality (US$ b)	Due to cognitive losses (US$ b)	Total cost (health, mortality and cognitive) (US$ b)	Total cost as % of GNI
East Asia and Pacific	10.39 (2.71, 49.67)	0.66 (0.55, 0.80)	74.76 (19.46, 357.66)	86.12 (23.04, 408.45)	0.59 (0.16, 2.78)
Europe and Central Asia	1.35 (0.35, 6.38)	0.25 (0.21, 0.29)	14.76 (3.86, 70.15)	16.27 (4.37, 76.71)	0.42 (0.11, 1.97)
Middle East and North Africa	3.76 (0.98, 17.99)	0.03 (0.03, 0.04)	18.65 (4.85, 89.38)	22.57 (5.98, 107.54)	0.91 (0.24, 4.32)
Latin America and Caribbean	4.08 (1.07, 19.35)	0.28 (0.23, 0.34)	32.25 (8.41, 154.02)	36.85 (9.94, 173.95)	0.70 (0.19, 3.32)
North America	0.00 (0.00, 0.00)	0.00 (0.00, 0.00)	114.94 (29.87, 551.37)	114.97 (29.90, 551.40)	0.63 (0.16, 3.04)
South Asia	10.58 (2.75, 50.59)	0.02 (0.02, 0.02)	11.73 (3.05, 56.12)	22.49 (5.99, 106.90)	0.84 (0.22, 3.99)
Sub-Saharan Africa	23.56 (6.59, 101.00)	0.02 (0.01, 0.02)	18.31 (5.05, 80.05)	42.06 (11.82, 181.25)	2.58 (0.72, 11.11)
Low and middle income	53.57 (14.41, 244.27)	1.08 (0.90, 1.30)	162.55 (42.62, 769.48)	218.27 (59.01, 1, 016.13)	0.83 (0.22, 3.86)
High income	0.15 (0.04, 0.71)	0.18 (0.15, 0.22)	122.84 (31.92, 589.28)	123.06 (32.03, 590.06)	0.25 (0.06, 1.20)
Upper-middle income	14.44 (3.81, 67.79)	0.95 (0.80, 1.16)	114.07 (29.74, 544.54)	130.07 (34.95, 614.10)	0.65 (0.17, 3.07)
Lower-middle income	35.19 (9.53, 158.64)	0.12 (0.10, 0.14)	44.73 (11.88, 207.87)	80.47 (21.94, 367.09)	1.36 (0.37, 6.20)
Low income	3.95 (1.07, 17.83)	0.01 (0.00, 0.01)	3.74 (1.01, 17.07)	7.73 (2.12, 34.95)	1.99 (0.54, 8.98)
Total	53.72 (14.45, 244.99)	1.26 (1.06, 1.52)	285.39 (74.55, 1, 358.75)	341.33 (91.04, 1, 606.19)	0.70 (0.19, 3.29)

(1) Default scenario (not in parentheses) based on discount rate on benefits of 3% and long-term GDP growth rate assumption of 3%. Figures in parentheses are lower and higher bound estimates based on assumptions of 1.5% discount rate on benefits and 5% long-term GDP growth rate for the figure on the left and 5% discount rate on benefits and 1.5% long-term GDP growth rate for the figure on the right.

**Figure 3:**
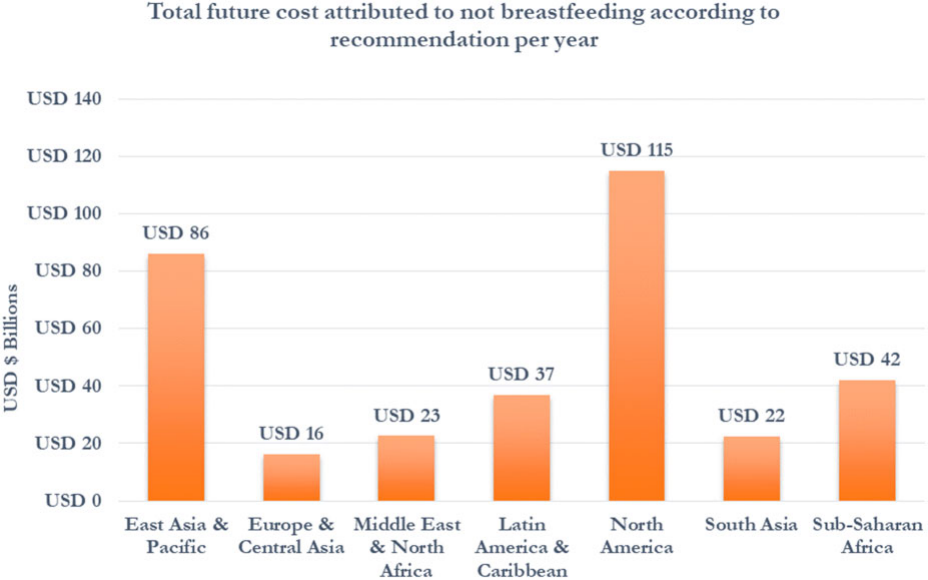
Total economic cost by region per year (US$ billion).

**Figure 4:**
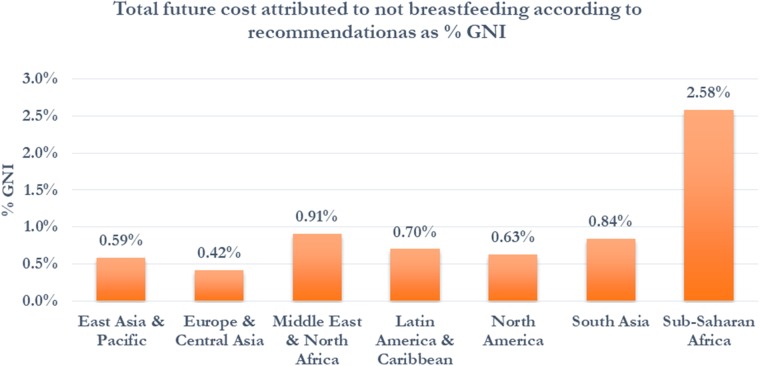
Total economic cost by region per year as percentage of GNI.

While mortality is a major contributor to economic losses for LMICs, the future economic losses due to cognitive losses in children are found to be much larger globally. Using data in 136 countries to calculate cognitive losses, the economic losses by persons who were not exclusively breastfed according to recommendations increased to US$285.39 billion annually (Table 4). The economic losses estimated for North America decreased to US$114.9 billion, or 43% of the total losses calculated. The economic losses for regions with a higher concentration of LMICs increased substantially with this approach. The economic losses estimated for East Asia and the Pacific, Latin America and the Caribbean, and Sub-Saharan Africa regions equal US$74.8, US$32.3, US$18.3 billion, respectively (Figure 3 and Table 4). Over 56% of the share of the total global economic losses is projected to be borne by LMICs (Table 4). The combined total economic losses, including health system cost, mortality losses and cognitive losses were estimated to be US$341.3 billion annually or 0.7% of global GNI (Table 4). The total economic losses as a share of GNI are highest in Sub-Saharan Africa at 2.58% followed by the Middle East and North Africa and Latin America and the Caribbean at 0.91% and 0.84%, respectively (Figure 4). In country income group, the total economic losses as a share of GNI are highest in low-income countries at 1.96% followed by lower middle-income countries at 1.35% of GNI (Table 4). The estimates of economic losses for all individual countries with data available can be found in Supplementary Appendix A6–8.

#### Sensitivity analysis

Estimating the economic losses far into the future comes with significant uncertainty. In the sensitivity analysis, the total global economic losses decreased to US$91.0 billion or 0.2% of GNI using conservative discounting and growth assumptions and increased to US$1.6 trillion or 3.29% of GNI with optimistic assumptions (Table 4). By calculating children’s cognitive losses with the alternative approach, which assumes that breastfeeding at 6 months is associated with a 2.62 point IQ increase compared with not being breastfed with data from 117 countries, the economic losses by persons who were not breastfeeding according to recommendations were estimated to equal US$210.13 billion annually. The total economic losses, which combine the health system costs, economic losses from mortality and cognitive losses, are therefore estimated to be US$257.9 billion annually or 0.37% of global GNI. For this alternative approach, the total global economic losses decreased to US$69.0 billion or 0.1% of GNI using conservative discounting and growth assumptions and increased to US$1.2 trillion or 1.75% of GNI with optimistic assumptions. Therefore, the potential future economic losses could vary widely, but regardless the economic consequences in all scenarios remain substantial. The key drivers of these economic losses estimated were by the number of children not breastfed according to recommendations, the scale of mortality due to sub-optimal breastfeeding, economic growth and discount rates selected for the analysis. Sensitivity analysis results for regions and income groups are listed in Table 4 and Supplementary Appendix Tables A6–8 for individual countries with data.

### Case study countries: China, India, Indonesia, Mexico and Nigeria

This section highlights the country-level results from the *Cost of Not Breastfeeding Tool* from five large emerging economies, which together contain 53% of the population of all developing countries, because of their strategic importance for achieving the Sustainable Development Goals.

#### China

In China, the prevalence of exclusive breastfeeding in children below the age of 6 months is 21% (UNICEF, 2017). An estimated 16 146 child deaths due to preventable diarrhoea and pneumonia were attributed to not breastfeeding according to recommendations and 22 537 deaths in women from cancers and type II diabetes each year (Table 5 and Supplementary Appendix Tables A3 and A4). The estimated health system cost of treatment of cases of childhood diarrhoea and pneumonia and mothers’ type II diabetes equals US$196 million per year (Supplementary Appendix Table A5). The economic loss of mortality, from the number of preventable deaths, was estimated to cost the Chinese economy approximately $6.3 billion each year (Supplementary Appendix Table A6). However, with a rapidly declining child mortality rate in China, cognitive losses, estimated to be US$59 billion (Supplementary Appendix Table A7), are the main driver of the total economic losses. Therefore, the total economic cost of not breastfeeding according to recommendation was estimated to be US$66.1 billion, which translates to 0.61 of China’s GNI (Table 5 and Supplementary Appendix Table A8).

**Table 5:** Total mortality and economic losses due to not breastfeeding for China, India, Indonesia, Mexico and Nigeria

Total human and economic costs attributable to not breastfeeding				
	Total child deaths	Total maternal deaths	Total cost (health, mortality and cognitive) (US$ b)	Total cost as % of GNI
China	16 146	22 537	66.1 (17.6, 314.1)	0.61 (0.16, 2.89)
India	99 552	11 404	14.5 (3.8, 68.9)	0.69 (0.18, 3.29)
Indonesia	15 028	5170	9.4 (2.5, 44.5)	1.05 (0.28, 5.01)
Mexico	2360	5195	8.2 (2.2, 39.2)	0.67 (0.18, 3.18)
Nigeria	103 742	1511	21.1 (5.8, 92.7)	4.10 (1.12, 18.04)

#### India

Despite a reported 55% prevalence of exclusive breastfeeding in children below the age of 6 months (UNICEF, 2017), there were an estimated 99 552 child deaths each year due to diarrhoea and pneumonia that could have been prevented with breastfeeding in India (Table 5 and Supplementary Appendix Table A3). The key drivers of this high mortality are India’s large population and high under-five mortality rate. The estimated health system cost of treatment of cases of childhood diarrhoea and pneumonia and women’s type II diabetes equals US$106 million per year (Supplementary Appendix Table A5). The high level of child mortality disproportionately affects the total economic cost of not breastfeeding in India, which was estimated to be US$14.5 billion, or 0.69% of GNI (Supplementary Appendix Table A8).

#### Indonesia

In Indonesia, the prevalence of exclusive breastfeeding in children below the age of 6 months was at 42% (UNICEF, 2017). At this level, it was estimated that there were 15 028 child deaths and 5174 deaths in women each year linked to not breastfeeding according to recommendations (Table 5 and Supplementary Appendix Tables A3 and A4). The estimated health system cost of treatment of cases of childhood diarrhoea and pneumonia and women’s type II diabetes equals US$85 million per year (Supplementary Appendix Table A5). The economic cost of mortality was a key driver of total economic losses in Indonesia, which are estimated to be US$9.3 billion, or 1.05% of its GNI (Supplementary Appendix Table A8).

#### Mexico

Approximately 31% of children below the age of 6 months in Mexico were exclusively breastfed (UNICEF, 2017). It was estimated that 2360 child deaths were due to preventable diarrhoea and pneumonia and 5195 women’s deaths were from cancers and type II diabetes each year, all of which are attributed to not breastfeeding according to recommendations (Table 5). The health system cost was estimated to be US$47 million per year (Supplementary Appendix Tables A5). Primarily driven by cognitive losses equal to US$7.1 billion (Supplementary Appendix Tables A7), the total economic losses in Mexico were estimated to be US$8.2 billion, or 0.67% of its GNI (Supplementary Appendix Tables A8).

#### Nigeria

In Nigeria, the prevalence of exclusive breastfeeding in children below the age of 6 months was only 17% (UNICEF, 2017), which means that at least 5.4 million children each year do not get the full benefits of breastfeeding. Not breastfeeding according to recommendation was estimated to lead to 103 742 child deaths each year (Table 5). The estimated health system cost of treating cases of childhood diarrhoea and pneumonia and women’s type II diabetes was estimated to equal US$21.8 million per year (Supplementary Appendix Tables A3). The combined economic losses of health system cost, mortality losses, and cognitive losses are estimated to be and US$21.0 billion, or 4.1% of its GNI.

## Discussion

The study found that the total global costs of not breastfeeding were estimated at 694 322 lives lost annually and economic losses of US$341.3 billion. Key drivers of the economic losses included low exclusive and continued breastfeeding rates, high child mortality rates and high incomes, which compounded with income growth into higher economic losses in the future. This analysis showed that the economic costs of mortality should not be forgotten in economic evaluations, since the mortality losses may be as significant as cognitive losses—particularly for countries with high mortality rates and low breastfeeding rates. The human and economic costs of not breastfeeding highlight the continued importance of enabling a culture to better support and protect breastfeeding globally.

Overall the results of this study were aligned with that of previous studies (Rollins et al., 2016; Victora *et al.*, 2016), and any differences in results can be explained by changes in the model design and the requirement for open access data for analysis by the tool. The sensitivity analysis of this study demonstrated that the economic losses could be even greater than estimated with potential accelerated economic growth in some countries. The new *Cost of Not Breastfeeding Tool*, however, provides policy-makers and other stakeholders with access to an evidence-based, customizable analysis that can be helpful for creating their investment cases and telling the complex story of breastfeeding and nutrition.

### Policy implications

There are several policy implications of this study to consider. First, the interpretation of health system treatment costs must be informed by local context since the absolute figures on health system cost since the lower health system costs in some settings may reflect lack of universal access to publicly funded maternal and child health services rather than optimal breastfeeding practices. With improved breastfeeding rates, the health system costs could be converted into health budget cost-savings and, in turn, redirected as allocations for breastfeeding promotion interventions. Second, formula feeding is neither beneficial for improving child health and cognitive development nor affordable for the vast majority of families living in LMICs and poor households globally. Third, the concentration of the global costs of not breastfeeding in China, India, Indonesia, Mexico and Nigeria, which together totalled 282 645 lives lost and up to US$119 billion in economic losses each year, is noteworthy. These large emerging economies need to pay more attention to the growing double-burden of malnutrition since both the human and economic costs of under- and over-nutrition were high. From a utilitarian perspective, this level of concentration could also motivate donors to invest in a few strategic countries to better the chances of achieving the Sustainable Development Goals or WHA Global Nutrition Targets. However, donors and policy-makers need to balance efficiency with equity in the investment prioritization process. It could be very feasible for governments of these countries and other middle-income countries to finance the cost of breastfeeding promotion interventions from domestic sources. International donor investment may be better focused on countries where the cost of not breastfeeding as a share of GNI was the highest.

### Limitations

While the tool provides certain advantages in improving access to data on the human and economic costs of not breastfeeding, there remain some gaps that could be improved upon in future versions of the tool. Many of the current limitations, including the exclusion of human and economic consequences from nearly 100 countries (mostly smaller countries) and additional economic costs related to caregiver time, which are mainly borne by women (Leslie, 1989) as well as household treatment fees, childhood diabetes, childhood cancers, sudden infant death syndrome, necrotizing enterocolitis, preterm births, post-partum haemorrhage and depression due to lack of data, result in conservative estimates from this model. Updating the tool with new data on the cost of care by age group and disease category and sensitivity analyses using the lower and upper bound relative risks of the effect of not breastfeeding on infectious illness and cognitive development would improve the accuracy of estimates. A more systematic collection of formula cost across countries would also be helpful. It should also be noted that the tool computes the total economic costs of not breastfeeding at current prevalence levels compared with universal breastfeeding; however, it may be more feasible in the future to compute the costs of not breastfeeding relative to country-specific targets. While the tool may provide policy-makers with improved access to data on human and economic costs of not breastfeeding, the tool does not replace the value of detailed economic evaluations in individual countries, which should supersede the results from this global-level analysis.

## Conclusion

The *Cost of Not Breastfeeding Tool* is an example how the big data revolution in global health can support stronger advocacy and meaningful policy change. More research and development of analytical knowledge translation tools may help educate policy-makers on the economics benefits, to children, women and caregivers, households, governments and societies as a whole, related to breastfeeding and nutrition. The substantial human and economic costs of not breastfeeding in countries with low breastfeeding rates can to some extent be reversed with government, donor and civil society action to increase the financing envelope available for the evidence-based high-impact breastfeeding and nutrition interventions and policies. Investments made now will accelerate progress towards the WHA Global Nutrition Targets and the SDGs as well as boost human capital development in the long-run.

## Supplementary Material

czz050_Supplementary_Appendix

## Footnotes

1 The *Cost of Not Breastfeeding Tool* (version 1) is now available to the public at the www.aliveandthrive.org/costofnotbreastfeeding.

2 All aggregated results are presented using the World Bank Group’s classification systems of regions (i.e. East Asia and Pacific, Europe and Central Asia, Middle East and North Africa, Latin America and Caribbean, North America, South Asia and Sub-Saharan Africa) and income groups (i.e. high income, upper-middle income, lower-middle income and low income) (World Bank, 2017).

3 The main analysis of economic losses in this article were conducted using the default assumptions of a long-term mean GDP growth rate of 3% and discount rate on benefits of 3%.

4 Sensitivity is also presented using conservative assumptions of a long-term GDP growth rate of 1.5% and a discount rate on benefits of 5% as well as optimistic assumptions of a long-term GDP growth rate of 5% and a discount rate on benefits of 1.5%.

5 This alternative approach to calculating cognitive losses assumes that the children who are breastfed until at least 6 months receive the cognitive benefits of breastfeeding over children not breastfed to 6 months as described in the Lancet Series on Breastfeeding (Rollins *et al.* 2016).
