# Supplementary material for: The cost of not breastfeeding: global results from a new tool
Source: Health Policy Plan. 2019 Jun 24;34(6):407–17. doi: 10.1093/heapol/czz050 (PMC6735804; doi:10.1093/heapol/czz050)
Supplement: czz050_Supplementary_Appendix [file czz050_supplementary_appendix.docx]

**WEB APPENDIX**

**WEB APPENDIX TABLE 1**. Health system treatment unit cost data collected for five countries (US$).

|  | **Country** | **China** | **Ethiopia** | **Ghana** | **Mexico** | **Nigeria** | **Philippines** |
| --- | --- | --- | --- | --- | --- | --- | --- |
| **Diarrhea (0-5 months)** | Cost of outpatient care at health center level per day | 7.7 | 1.1 | 5.7 | 44.7 | 6.0 | 6.3 |
|  | Cost of outpatient in primary level hospital per day | 10.6 | 2.4 | 5.7 | 44.8 | 7.4 | 6.3 |
|  | Cost of outpatient in secondary level hospital per day | 6.9 | 3.3 | 5.7 | 48.7 | 6.4 | 6.3 |
|  | Cost of inpatient in primary hospital per day | 71.2 | 0.5 | Not Available | 396.7 | 3.9 | 14.2 |
|  | Mean number of day for inpatient treatment at primary hospital | 6.5 | 8.4 | Not Available | 1.2 | 5.0 | 8.4 |
|  | Cost of inpatient in secondary hospital per day | 40.7 | 2.3 | Not Available | 396.9 | 4.1 | 14.2 |
|  | Mean number of day for inpatient treatment at secondary hospital | 6.5 | 8.4 | Not Available | 2.8 | 5.0 | 8.4 |
|  | Cost of inpatient in teaching/tertiary/referral hospital per day | 79.3 | 2.3 | Not Available | 400.7 | 6.8 | 14.2 |
|  | Mean number of days for inpatient treatment at teaching/tertiary/referral hospital | 6.5 | 8.4 | Not Available | 4.3 | 5.0 | 8.4 |
| **Diarrhea (6-23 months)** | Cost of outpatient care at health center level per day | 20.4 | 1.1 | 5.7 | 44.7 | 6.0 | 6.3 |
|  | Cost of outpatient in primary level hospital per day | 11.2 | 2.4 | 5.7 | 44.8 | 7.4 | 6.3 |
|  | Cost of outpatient in secondary level hospital per day | 10.4 | 3.3 | 5.7 | 48.7 | 6.4 | 6.3 |
|  | Cost of inpatient in primary hospital per day | 72.4 | 0.5 | Not Available | 396.7 | 3.9 | 14.2 |
|  | Mean number of days for inpatient treatment at primary hospital | 6.5 | 8.4 | Not Available | 1.2 | 5.0 | 8.4 |
|  | Cost of inpatient in secondary hospital per day | 41.2 | 2.3 | Not Available | 396.9 | 4.1 | 14.2 |
|  | Mean number of days for inpatient treatment at secondary hospital | 6.5 | 8.4 | Not Available | 2.8 | 5.0 | 8.4 |
|  | Cost of inpatient in teaching/tertiary/referral hospital per day | 79.3 | 2.3 | Not Available | 400.7 | 6.8 | 14.2 |
|  | Mean number of days for inpatient treatment at teaching/tertiary/referral hospital | 6.5 | 8.4 | Not Available | 4.3 | 5.0 | 8.4 |
| **Pneumonia (0-5 months)** | Cost of outpatient care at health center level per day | 18.7 | 1.6 | 5.7 | 45.3 | 8.5 | 6.2 |
|  | Cost of outpatient in primary level hospital per day | 28.2 | 1.7 | 5.7 | 45.5 | 10.0 | 6.2 |
|  | Cost of outpatient in secondary level hospital per day | 31.2 | 6.2 | 5.7 | 49.3 | 14.8 | 6.2 |
|  | Cost of inpatient in primary hospital per day | 96.5 | 0.7 | Not Available | 398.3 | 2.8 | 47.8 |
|  | Mean number of days for inpatient treatment at primary hospital | 8.6 | 6.4 | Not Available | 1.5 | 7.0 | 6.4 |
|  | Cost of inpatient in secondary hospital per day | 45.0 | 4.1 | Not Available | 398.4 | 1.8 | 47.8 |
|  | Mean number of days for inpatient treatment at secondary hospital | 8.6 | 6.4 | Not Available | 4.8 | 7.0 | 6.4 |
|  | Cost of inpatient in teaching/tertiary/referral hospital per day | 84.7 | 4.1 | Not Available | 402.3 | 4.7 | 47.8 |
|  | Mean number of days for inpatient treatment at teaching/tertiary/referral hospital | 8.6 | 6.4 | Not Available | 9.1 | 7.0 | 6.4 |
| **Pneumonia (6-23 months)** | Cost of outpatient care at health center level per day | 53.5 | 1.6 | 5.7 | 45.3 | 8.5 | 6.2 |
|  | Cost of outpatient in primary level hospital per day | 28.0 | 1.7 | 5.7 | 45.5 | 10.0 | 6.2 |
|  | Cost of outpatient in secondary level hospital per day | 29.0 | 6.2 | 5.7 | 49.3 | 14.8 | 6.2 |
|  | Cost of inpatient in primary hospital per day | 78.4 | 0.7 | Not Available | 398.3 | 2.8 | 47.8 |
|  | Mean number of days for inpatient treatment at primary hospital | 8.6 | 6.4 | Not Available | 1.5 | 7.0 | 6.4 |
|  | Cost of inpatient in secondary hospital per day | 44.6 | 4.1 | Not Available | 398.4 | 1.8 | 47.8 |
|  | Mean number of days for inpatient treatment at secondary hospital | 8.6 | 6.4 | Not Available | 4.8 | 7.0 | 6.4 |
|  | Cost of inpatient in teaching/tertiary/referral hospital per day | 78.5 | 4.1 | Not Available | 402.3 | 4.7 | 47.8 |
|  | Mean number of days for inpatient treatment at teaching/tertiary/referral hospital | 8.6 | 6.4 | Not Available | 9.1 | 7.0 | 6.4 |

**WEB APPENDIX TABLE 2**. Unit cost data for the price of formula collected from online vendors by country.

| **Country** | **Unit cost for price of lowest economy brand of formula per 900-gram container (US$)** |
| --- | --- |
| **Afghanistan** | Not Available |
| **Albania** | Not Available |
| **Algeria** | 13.41 |
| **American Samoa** | Not Available |
| **Andorra** | Not Available |
| **Angola** | Not Available |
| **Antigua and Barbuda** | Not Available |
| **Argentina** | 11.64 |
| **Armenia** | 18.78 |
| **Aruba** | 20.67 |
| **Australia** | 22.54 |
| **Austria** | 15.55 |
| **Azerbaijan** | 36.17 |
| **Bahamas, The** | 16.99 |
| **Bahrain** | 22.11 |
| **Bangladesh** | 30.15 |
| **Barbados** | 22.49 |
| **Belarus** | 19.14 |
| **Belgium** | 13.62 |
| **Belize** | Not Available |
| **Benin** | Not Available |
| **Bermuda** | 43.69 |
| **Bhutan** | Not Available |
| **Bolivia** | Not Available |
| **Bosnia and Herzegovina** | Not Available |
| **Botswana** | 11.41 |
| **Brazil** | 13.49 |
| **British Virgin Islands** | Not Available |
| **Brunei** | Not Available |
| **Bulgaria** | 16.31 |
| **Burkina Faso** | Not Available |
| **Burundi** | Not Available |
| **Cabo Verde** | Not Available |
| **Cambodia** | 14.63 |
| **Cameroon** | Not Available |
| **Canada** | 17.80 |
| **Cayman Islands** | Not Available |
| **Central African Republic** | Not Available |
| **Chad** | Not Available |
| **Channel Islands** | Not Available |
| **Chile** | 37.83 |
| **China** | 15.90 |
| **Colombia** | 37.17 |
| **Comoros** | Not Available |
| **Congo** | Not Available |
| **Costa Rica** | 23.68 |
| **Côte d'Ivoire** | Not Available |
| **Croatia** | 20.67 |
| **Cuba** | Not Available |
| **Curacao** | Not Available |
| **Cyprus** | 18.17 |
| **Czech Republic** | 15.05 |
| **Democratic Republic of the Congo** | Not Available |
| **Denmark** | 12.70 |
| **Djibouti** | Not Available |
| **Dominica** | Not Available |
| **Dominican Republic** | 12.12 |
| **Ecuador** | Not Available |
| **Egypt** | 29.25 |
| **El Salvador** | 18.49 |
| **Equatorial Guinea** | Not Available |
| **Eritrea** | Not Available |
| **Estonia** | 14.37 |
| **Ethiopia** | Not Available |
| **Faroe Islands** | Not Available |
| **Fiji** | 12.29 |
| **Finland** | 10.68 |
| **France** | 14.31 |
| **French Polynesia** | Not Available |
| **Gabon** | Not Available |
| **Gambia, The** | Not Available |
| **Georgia** | Not Available |
| **Germany** | 14.96 |
| **Ghana** | 19.21 |
| **Gibraltar** | 21.42 |
| **Greece** | 19.99 |
| **Greenland** | Not Available |
| **Grenada** | Not Available |
| **Guam** | Not Available |
| **Guatemala** | Not Available |
| **Guinea** | Not Available |
| **Guinea-Bissau** | Not Available |
| **Guyana** | Not Available |
| **Haiti** | Not Available |
| **Honduras** | Not Available |
| **Hong Kong SAR, China** | 31.35 |
| **Hungary** | 11.81 |
| **Iceland** | Not Available |
| **India** | 12.88 |
| **Indonesia** | 8.66 |
| **Iran, Islamic Rep.** | Not Available |
| **Iraq** | Not Available |
| **Ireland** | 12.63 |
| **Isle of Man** | Not Available |
| **Israel** | 20.56 |
| **Italy** | 22.44 |
| **Jamaica** | 28.75 |
| **Japan** | 14.85 |
| **Jordan** | Not Available |
| **Kazakhstan** | Not Available |
| **Kenya** | 29.28 |
| **Kiribati** | Not Available |
| **Korea, Dem. People’s Rep.** | Not Available |
| **Korea, Rep.** | 16.91 |
| **Kosovo** | Not Available |
| **Kuwait** | 22.10 |
| **Kyrgyzstan** | Not Available |
| **Laos** | Not Available |
| **Latvia** | 11.56 |
| **Lebanon** | 15.26 |
| **Lesotho** | Not Available |
| **Liberia** | Not Available |
| **Libya** | Not Available |
| **Liechtenstein** | Not Available |
| **Lithuania** | 13.47 |
| **Luxembourg** | 13.93 |
| **Macao SAR, China** | Not Available |
| **Macedonia, FYR** | Not Available |
| **Madagascar** | Not Available |
| **Malawi** | Not Available |
| **Malaysia** | 13.77 |
| **Maldives** | Not Available |
| **Mali** | Not Available |
| **Malta** | 15.53 |
| **Marshall Islands** | Not Available |
| **Mauritania** | Not Available |
| **Mauritius** | 9.88 |
| **Mexico** | 13.84 |
| **Micronesia, Fed. Sts.** | Not Available |
| **Moldova** | Not Available |
| **Monaco** | Not Available |
| **Mongolia** | 13.70 |
| **Montenegro** | Not Available |
| **Morocco** | Not Available |
| **Mozambique** | Not Available |
| **Myanmar** | 15.48 |
| **Namibia** | Not Available |
| **Nauru** | Not Available |
| **Nepal** | 10.83 |
| **Netherlands** | 6.86 |
| **New Caledonia** | Not Available |
| **New Zealand** | 13.95 |
| **Nicaragua** | Not Available |
| **Niger** | Not Available |
| **Nigeria** | 25.14 |
| **Northern Mariana Islands** | Not Available |
| **Norway** | 19.47 |
| **Oman** | Not Available |
| **Pakistan** | 15.54 |
| **Palau** | Not Available |
| **Panama** | 16.81 |
| **Papua New Guinea** | Not Available |
| **Paraguay** | Not Available |
| **Peru** | 43.59 |
| **Philippines** | 10.90 |
| **Poland** | 13.13 |
| **Portugal** | 15.80 |
| **Puerto Rico** | Not Available |
| **Qatar** | 20.26 |
| **Romania** | 15.65 |
| **Russia** | 18.61 |
| **Rwanda** | 25.90 |
| **Samoa** | 17.32 |
| **San Marino** | Not Available |
| **Sao Tome and Principe** | Not Available |
| **Saudi Arabia** | 16.80 |
| **Senegal** | Not Available |
| **Serbia** | 14.06 |
| **Seychelles** | Not Available |
| **Sierra Leone** | Not Available |
| **Singapore** | 21.75 |
| **Sint Maarten (Dutch part)** | Not Available |
| **Slovak Republic** | Not Available |
| **Slovenia** | Not Available |
| **Solomon Islands** | Not Available |
| **Somalia** | Not Available |
| **South Africa** | 8.70 |
| **South Sudan** | Not Available |
| **Spain** | 12.39 |
| **Sri Lanka** | 18.88 |
| **St. Kitts and Nevis** | Not Available |
| **St. Lucia** | Not Available |
| **St. Martin (French part)** | Not Available |
| **St. Vincent and the Grenadines** | Not Available |
| **Sudan** | Not Available |
| **Suriname** | Not Available |
| **Swaziland** | 9.01 |
| **Sweden** | 12.00 |
| **Switzerland** | 27.47 |
| **Syria** | Not Available |
| **Tajikistan** | Not Available |
| **Tanzania** | Not Available |
| **Thailand** | 27.63 |
| **Timor-Leste** | Not Available |
| **Togo** | Not Available |
| **Tonga** | Not Available |
| **Trinidad and Tobago** | 16.15 |
| **Tunisia** | 27.41 |
| **Turkey** | 22.78 |
| **Turkmenistan** | Not Available |
| **Turks and Caicos Islands** | Not Available |
| **Tuvalu** | Not Available |
| **Uganda** | 25.43 |
| **Ukraine** | 28.12 |
| **United Arab Emirates** | 21.37 |
| **United Kingdom** | 17.19 |
| **United States** | 17.61 |
| **Uruguay** | Not Available |
| **Uzbekistan** | Not Available |
| **Vanuatu** | Not Available |
| **Venezuela** | Not Available |
| **Viet Nam** | 40.03 |
| **Virgin Islands (U.S.)** | Not Available |
| **West Bank and Gaza** | Not Available |
| **Yemen, Rep.** | Not Available |
| **Zambia** | 10.17 |
| **Zimbabwe** | 15.39 |

**WEB APPENDIX TABLE 3**. Child health disease outcomes attributable to not breastfeeding by country.

|  | **Number of cases of child diarrhea (0-23 months) attributable to not breastfeeding** | **Number of cases of child ARI/pneumonia (0-23 months) attributable to not breastfeeding** | **Number of child deaths (0-23months) due to diarrhea attributable to not breastfeeding** | **Number of child deaths (0-23months) due to ARI/pneumonia attributable to not breastfeeding** | **Number of cases of childhood obesity preventable with full breastfeeding** | **Total number of child deaths attributable to not breastfeeding** |
| --- | --- | --- | --- | --- | --- | --- |
| **Afghanistan** | 1,849,276 | 82,557 | 4,933 | 7,200 | 5,823 | 12,133 |
| **Albania** | 9,331 | 3,784 | 4 | 36 | 1,323 | 40 |
| **Algeria** | 1,357,756 | 71,868 | 663 | 1,702 | 20,830 | 2,365 |
| **American Samoa** | Not Available | Not Available | Not Available | Not Available | Not Available | Not Available |
| **Andorra** | Not Available | Not Available | Not Available | Not Available | Not Available | Not Available |
| **Angola** | 2,242,260 | 89,482 | 12,198 | 13,730 | 0 | 25,929 |
| **Antigua and Barbuda** | Not Available | Not Available | Not Available | Not Available | Not Available | Not Available |
| **Argentina** | 102,521 | 34,799 | 65 | 355 | 13,405 | 420 |
| **Armenia** | 33,656 | 2,767 | 4 | 35 | 1,419 | 39 |
| **Aruba** | Not Available | Not Available | Not Available | Not Available | Not Available | Not Available |
| **Australia** | Not Available | Not Available | Not Available | Not Available | Not Available | Not Available |
| **Austria** | Not Available | Not Available | Not Available | Not Available | Not Available | Not Available |
| **Azerbaijan** | 200,465 | 23,491 | 252 | 546 | 4,241 | 798 |
| **Bahamas** | Not Available | Not Available | Not Available | Not Available | Not Available | Not Available |
| **Bahrain** | 17,722 | 1,209 | Not Available | Not Available | Not Available | Not Available |
| **Bangladesh** | 4,484,389 | 229,195 | 1,867 | 4,081 | 1,412 | 5,948 |
| **Barbados** | 3,676 | 332 | Not Available | Not Available | Not Available | Not Available |
| **Belarus** | 32,060 | 7,293 | 2 | 20 | 2,440 | 21 |
| **Belgium** | 7,356 | 2,936 | Not Available | Not Available | Not Available | Not Available |
| **Belize** | 10,201 | 744 | 2 | 6 | 101 | 8 |
| **Benin** | 514,364 | 22,948 | 1,938 | 2,728 | 785 | 4,666 |
| **Bermuda** | Not Available | Not Available | Not Available | Not Available | Not Available | Not Available |
| **Bhutan** | 58,819 | 1,162 | 10 | 24 | 109 | 34 |
| **Bolivia** | 553,606 | 22,862 | 249 | 489 | 3,195 | 738 |
| **Bosnia and Herzegovina** | 10,285 | 2,742 | 0 | 6 | 1,453 | 6 |
| **Botswana** | 82,871 | 3,358 | 91 | 168 | 1,382 | 260 |
| **Brazil** | 4,700,756 | 298,566 | 732 | 2,687 | 41,018 | 3,419 |
| **British Virgin Islands** | Not Available | Not Available | Not Available | Not Available | Not Available | Not Available |
| **Brunei** | Not Available | Not Available | Not Available | Not Available | Not Available | Not Available |
| **Bulgaria** | Not Available | Not Available | Not Available | Not Available | Not Available | Not Available |
| **Burkina Faso** | 1,356,078 | 31,894 | 1,447 | 1,961 | 868 | 3,408 |
| **Burundi** | 892,836 | 28,267 | 796 | 1,139 | 533 | 1,935 |
| **Cabo Verde** | Not Available | Not Available | 7 | 18 | 0 | 25 |
| **Cambodia** | 478,324 | 21,408 | 321 | 776 | 1,154 | 1,097 |
| **Cameroon** | 1,622,416 | 47,925 | 4,323 | 5,359 | 9,781 | 9,682 |
| **Canada** | Not Available | Not Available | Not Available | Not Available | Not Available | Not Available |
| **Cayman Islands** | Not Available | Not Available | Not Available | Not Available | Not Available | Not Available |
| **Central African Republic** | 330,855 | 15,517 | 1,065 | 1,352 | 419 | 2,418 |
| **Chad** | 1,404,174 | 68,574 | 4,700 | 6,841 | 1,103 | 11,541 |
| **Channel Islands** | Not Available | Not Available | Not Available | Not Available | Not Available | Not Available |
| **Chile** | Not Available | Not Available | Not Available | Not Available | Not Available | Not Available |
| **China** | 4,695,212 | 423,511 | 3,081 | 13,065 | 254,825 | 16,146 |
| **Colombia** | 1,017,195 | 52,731 | 125 | 586 | 6,388 | 712 |
| **Comoros** | 51,825 | 2,096 | 66 | 138 | Not Available | 205 |
| **Congo** | 308,646 | 9,368 | 284 | 471 | 1,959 | 754 |
| **Costa Rica** | 83,427 | 5,386 | 4 | 19 | 1,098 | 22 |
| **Côte d'Ivoire** | Not Available | Not Available | 3,021 | 5,759 | 3,526 | 8,781 |
| **Croatia** | 5,827 | 1,976 | Not Available | Not Available | Not Available | Not Available |
| **Cuba** | 83,857 | 7,902 | 5 | 40 | 0 | 44 |
| **Curaçao** | Not Available | Not Available | Not Available | Not Available | Not Available | Not Available |
| **Cyprus** | Not Available | Not Available | Not Available | Not Available | Not Available | Not Available |
| **Czech Republic** | Not Available | Not Available | Not Available | Not Available | Not Available | Not Available |
| **Democratic People's Republic of Korea** | Not Available | Not Available | 269 | 601 | 0 | 870 |
| **Denmark** | Not Available | Not Available | Not Available | Not Available | Not Available | Not Available |
| **Djibouti** | 41,574 | 2,019 | 68 | 109 | 340 | 178 |
| **Dominica** | Not Available | Not Available | Not Available | Not Available | Not Available | Not Available |
| **Dominican Republic** | 395,178 | 26,090 | 145 | 477 | 3,684 | 622 |
| **Democratic Republic of the Congo** | 7,923,698 | 230,072 | 11,705 | 14,887 | 9,986 | 26,592 |
| **Ecuador** | 548,145 | 20,736 | 162 | 487 | 5,069 | 649 |
| **Egypt** | 3,525,441 | 172,019 | 1,713 | 4,083 | 69,636 | 5,797 |
| **El Salvador** | 103,182 | 7,046 | 40 | 91 | 705 | 131 |
| **Equatorial Guinea** | 51,928 | 2,534 | 122 | 245 | 581 | 367 |
| **Eritrea** | 265,130 | 15,207 | 194 | 333 | 218 | 527 |
| **Estonia** | Not Available | Not Available | Not Available | Not Available | Not Available | Not Available |
| **Ethiopia** | 4,622,831 | 184,863 | 4,849 | 8,882 | 4,660 | 13,732 |
| **Faroe Islands** | Not Available | Not Available | Not Available | Not Available | Not Available | Not Available |
| **Fiji** | 16,356 | 1,972 | Not Available | Not Available | Not Available | Not Available |
| **Finland** | Not Available | Not Available | Not Available | Not Available | Not Available | Not Available |
| **France** | Not Available | Not Available | Not Available | Not Available | Not Available | Not Available |
| **French Polynesia** | Not Available | Not Available | Not Available | Not Available | Not Available | Not Available |
| **Gabon** | 90,727 | 4,167 | 98 | 217 | 878 | 316 |
| **Gambia** | Not Available | Not Available | 217 | 289 | 274 | 506 |
| **Georgia** | 36,386 | 2,630 | 3 | 27 | 2,267 | 31 |
| **Germany** | Not Available | Not Available | Not Available | Not Available | Not Available | Not Available |
| **Ghana** | 1,328,739 | 47,231 | 1,509 | 2,265 | 2,564 | 3,774 |
| **Gibraltar** | Not Available | Not Available | Not Available | Not Available | Not Available | Not Available |
| **Greece** | Not Available | Not Available | Not Available | Not Available | Not Available | Not Available |
| **Greenland** | Not Available | Not Available | Not Available | Not Available | Not Available | Not Available |
| **Grenada** | 2,988 | 212 | Not Available | Not Available | Not Available | Not Available |
| **Guam** | Not Available | Not Available | Not Available | Not Available | Not Available | Not Available |
| **Guatemala** | 882,756 | 39,226 | 382 | 824 | 2,131 | 1,206 |
| **Guinea** | 834,024 | 32,205 | 1,403 | 2,544 | 1,291 | 3,947 |
| **Guinea-Bissau** | 115,700 | 3,569 | 246 | 433 | 158 | 679 |
| **Guyana** | 24,732 | 1,369 | 17 | 23 | 93 | 40 |
| **Haiti** | 701,635 | 31,609 | 947 | 1,858 | 1,598 | 2,805 |
| **Honduras** | 373,945 | 15,369 | 128 | 182 | 1,275 | 309 |
| **Hong Kong** | Not Available | Not Available | Not Available | Not Available | Not Available | Not Available |
| **Hungary** | Not Available | Not Available | Not Available | Not Available | Not Available | Not Available |
| **Iceland** | Not Available | Not Available | Not Available | Not Available | Not Available | Not Available |
| **India** | 34,791,524 | 2,470,429 | 41,882 | 57,669 | 40,382 | 99,552 |
| **Indonesia** | 9,044,515 | 306,200 | 3,861 | 11,167 | 62,408 | 15,028 |
| **Iran** | 1,430,182 | 85,952 | 390 | 1,309 | 0 | 1,698 |
| **Iraq** | 1,806,871 | 109,270 | 1,198 | 3,268 | 25,874 | 4,466 |
| **Ireland** | Not Available | Not Available | Not Available | Not Available | Not Available | Not Available |
| **Isle of Man** | Not Available | Not Available | Not Available | Not Available | Not Available | Not Available |
| **Israel** | Not Available | Not Available | Not Available | Not Available | Not Available | Not Available |
| **Italy** | Not Available | Not Available | Not Available | Not Available | Not Available | Not Available |
| **Jamaica** | 57,610 | 4,455 | 6 | 24 | 585 | 30 |
| **Japan** | Not Available | Not Available | Not Available | Not Available | Not Available | Not Available |
| **Jordan** | 259,172 | 16,160 | 57 | 199 | 2,011 | 255 |
| **Kazakhstan** | 216,092 | 23,672 | 172 | 378 | 10,338 | 551 |
| **Kenya** | 2,111,799 | 88,122 | 2,239 | 4,166 | 6,929 | 6,405 |
| **Kiribati** | 3,077 | 279 | 4 | 7 | 0 | 11 |
| **Kosovo** | Not Available | Not Available | Not Available | Not Available | Not Available | Not Available |
| **Kuwait** | 52,297 | 4,947 | 2 | 30 | 1,403 | 32 |
| **Kyrgyzstan** | 147,558 | 12,896 | 99 | 263 | 1,997 | 362 |
| **Laos** | 195,044 | 10,522 | 642 | 955 | 521 | 1,597 |
| **Latvia** | Not Available | Not Available | Not Available | Not Available | Not Available | Not Available |
| **Lebanon** | 103,606 | 7,112 | 10 | 27 | 3,331 | 37 |
| **Lesotho** | 107,403 | 2,947 | 262 | 445 | 712 | 707 |
| **Liberia** | 309,800 | 9,088 | 424 | 735 | 627 | 1,159 |
| **Libya** | Not Available | Not Available | Not Available | Not Available | Not Available | Not Available |
| **Liechtenstein** | Not Available | Not Available | Not Available | Not Available | Not Available | Not Available |
| **Lithuania** | Not Available | Not Available | Not Available | Not Available | Not Available | Not Available |
| **Luxembourg** | Not Available | Not Available | Not Available | Not Available | Not Available | Not Available |
| **Macao SAR, China** | Not Available | Not Available | Not Available | Not Available | Not Available | Not Available |
| **Madagascar** | 1,417,110 | 60,381 | 1,047 | 1,690 | 0 | 2,738 |
| **Malawi** | 1,265,211 | 43,495 | 1,037 | 1,785 | 2,149 | 2,821 |
| **Malaysia** | 421,618 | 20,347 | Not Available | Not Available | Not Available | Not Available |
| **Maldives** | 6,280 | 245 | 0 | 2 | 38 | 2 |
| **Mali** | 1,158,450 | 41,677 | 3,520 | 4,656 | 3,532 | 8,176 |
| **Malta** | Not Available | Not Available | Not Available | Not Available | Not Available | Not Available |
| **Marshall Islands** | 1,732 | 240 | 2 | 4 | Not Available | 5 |
| **Mauritania** | 240,991 | 8,387 | 550 | 649 | 218 | 1,200 |
| **Mauritius** | 11,525 | 686 | 3 | 13 | 246 | 15 |
| **Mexico** | 1,302,188 | 72,953 | 496 | 1,864 | 40,825 | 2,360 |
| **Micronesia** | Not Available | Not Available | Not Available | Not Available | Not Available | Not Available |
| **Moldova** | Not Available | Not Available | Not Available | Not Available | Not Available | Not Available |
| **Monaco** | Not Available | Not Available | Not Available | Not Available | Not Available | Not Available |
| **Mongolia** | 47,552 | 6,157 | 43 | 101 | 828 | 144 |
| **Montenegro** | 1,444 | 579 | 0 | 1 | 409 | 1 |
| **Morocco** | 1,057,597 | 60,201 | 513 | 1,316 | 13,742 | 1,829 |
| **Mozambique** | 1,414,097 | 55,501 | 3,210 | 4,676 | 9,070 | 7,887 |
| **Myanmar** | 938,709 | 47,944 | 1,305 | 2,924 | 2,292 | 4,229 |
| **Namibia** | 131,259 | 3,889 | 160 | 316 | 530 | 476 |
| **Nauru** | Not Available | Not Available | 0 | 0 | Not Available | 0 |
| **Nepal** | 1,456,605 | 39,994 | 263 | 574 | 369 | 837 |
| **Netherlands** | Not Available | Not Available | Not Available | Not Available | Not Available | Not Available |
| **New Caledonia** | Not Available | Not Available | Not Available | Not Available | Not Available | Not Available |
| **New Zealand** | Not Available | Not Available | Not Available | Not Available | Not Available | Not Available |
| **Nicaragua** | 203,867 | 11,611 | 105 | 207 | 1,143 | 312 |
| **Niger** | 1,921,239 | 61,115 | 4,493 | 7,337 | 2,924 | 11,830 |
| **Nigeria** | 9,782,090 | 475,562 | 39,842 | 63,899 | 17,628 | 103,742 |
| **Northern Mariana Islands** | Not Available | Not Available | Not Available | Not Available | Not Available | Not Available |
| **Norway** | Not Available | Not Available | Not Available | Not Available | Not Available | Not Available |
| **Oman** | 71,983 | 4,268 | 6 | 26 | 165 | 32 |
| **Pakistan** | 13,252,833 | 515,029 | 17,558 | 26,744 | 26,030 | 44,302 |
| **Palau** | Not Available | Not Available | Not Available | Not Available | Not Available | Not Available |
| **Panama** | 130,383 | 6,626 | 35 | 90 | 0 | 125 |
| **Papua New Guinea** | 195,444 | 25,731 | 288 | 670 | 1,996 | 958 |
| **Paraguay** | 212,804 | 28,264 | 98 | 182 | 3,534 | 280 |
| **Peru** | 829,499 | 42,428 | 194 | 377 | 8,065 | 571 |
| **Philippines** | 2,833,939 | 150,394 | 2,634 | 6,290 | 16,874 | 8,924 |
| **Poland** | Not Available | Not Available | Not Available | Not Available | Not Available | Not Available |
| **Portugal** | Not Available | Not Available | Not Available | Not Available | Not Available | Not Available |
| **Puerto Rico** | Not Available | Not Available | Not Available | Not Available | Not Available | Not Available |
| **Qatar** | 21,933 | 1,715 | 1 | 4 | 453 | 5 |
| **Republic of Korea** | Not Available | Not Available | Not Available | Not Available | Not Available | Not Available |
| **Romania** | 43,526 | 21,576 | Not Available | Not Available | Not Available | Not Available |
| **Russia** | Not Available | Not Available | Not Available | Not Available | Not Available | Not Available |
| **Rwanda** | 550,080 | 16,537 | 176 | 329 | 881 | 505 |
| **Saint Kitts and Nevis** | Not Available | Not Available | Not Available | Not Available | Not Available | Not Available |
| **Saint Lucia** | Not Available | Not Available | Not Available | Not Available | Not Available | Not Available |
| **Saint Martin** | Not Available | Not Available | Not Available | Not Available | Not Available | Not Available |
| **Saint Vincent and the Grenadines** | Not Available | Not Available | Not Available | Not Available | Not Available | Not Available |
| **Samoa** | 4,114 | 562 | 1 | 3 | 21 | 4 |
| **San Marino** | Not Available | Not Available | Not Available | Not Available | Not Available | Not Available |
| **Sao Tome and Principe** | 11,643 | 275 | 10 | 11 | 130 | 21 |
| **Saudi Arabia** | Not Available | Not Available | Not Available | Not Available | 7,147 | Not Available |
| **Senegal** | 1,187,682 | 30,901 | 1,076 | 1,547 | 851 | 2,624 |
| **Serbia** | 16,757 | 6,080 | 0 | 18 | 2,950 | 18 |
| **Seychelles** | Not Available | Not Available | Not Available | Not Available | Not Available | Not Available |
| **Sierra Leone** | 397,369 | 16,984 | 1,292 | 1,594 | 2,360 | 2,886 |
| **Singapore** | Not Available | Not Available | Not Available | Not Available | Not Available | Not Available |
| **Sint Maarten (Dutch part)** | Not Available | Not Available | Not Available | Not Available | Not Available | Not Available |
| **Slovakia** | Not Available | Not Available | Not Available | Not Available | Not Available | Not Available |
| **Slovenia** | Not Available | Not Available | Not Available | Not Available | Not Available | Not Available |
| **Solomon Islands** | 15,556 | 1,391 | 11 | 25 | 34 | 36 |
| **Somalia** | 855,882 | 44,355 | 4,907 | 7,885 | 2,042 | 12,792 |
| **South Africa** | 1,476,565 | 67,113 | 2,111 | 4,047 | 27,796 | 6,159 |
| **South Sudan** | 881,861 | 39,238 | 1,603 | 3,802 | 3,588 | 5,405 |
| **Spain** | Not Available | Not Available | Not Available | Not Available | Not Available | Not Available |
| **Sri Lanka** | 156,741 | 7,408 | 15 | 56 | 86 | 71 |
| **Sudan** | 2,751,130 | 118,268 | 4,015 | 5,781 | Not Available | 9,796 |
| **Suriname** | 14,371 | 1,058 | 2 | 9 | 86 | 10 |
| **Swaziland** | 64,951 | 2,271 | 127 | 188 | 729 | 315 |
| **Sweden** | Not Available | Not Available | Not Available | Not Available | Not Available | Not Available |
| **Switzerland** | Not Available | Not Available | Not Available | Not Available | Not Available | Not Available |
| **Syria** | 488,238 | 32,478 | 457 | 225 | 15,895 | 682 |
| **Taiwan** | Not Available | Not Available | Not Available | Not Available | Not Available | Not Available |
| **Tajikistan** | 366,078 | 22,373 | 430 | 929 | 1,839 | 1,359 |
| **Tanzania** | 3,170,944 | 106,126 | 3,567 | 6,018 | 9,350 | 9,586 |
| **Thailand** | 580,632 | 39,312 | 149 | 494 | 18,728 | 643 |
| **The former Yugoslav Republic of Macedonia** | Not Available | Not Available | 1 | 4 | 619 | 5 |
| **Timor-Leste** | 51,935 | 1,799 | 111 | 212 | 307 | 323 |
| **Togo** | 482,446 | 17,887 | 605 | 962 | 447 | 1,568 |
| **Tonga** | 2,206 | 234 | 1 | 2 | 83 | 3 |
| **Trinidad and Tobago** | 25,009 | 1,734 | 1 | 21 | 194 | 22 |
| **Tunisia** | 260,325 | 10,307 | 40 | 134 | 5,566 | 174 |
| **Turkey** | 1,584,206 | 113,692 | 83 | 377 | 25,145 | 461 |
| **Turkmenistan** | 112,463 | 10,037 | 246 | 460 | 0 | 705 |
| **Turks and Caicos Islands** | Not Available | Not Available | Not Available | Not Available | Not Available | Not Available |
| **Tuvalu** | Not Available | Not Available | 0 | 0 | Not Available | 0 |
| **Uganda** | 2,930,664 | 112,783 | 2,968 | 5,188 | 11,715 | 8,156 |
| **Ukraine** | 179,985 | 27,480 | 39 | 215 | 0 | 254 |
| **United Arab Emirates** | 101,988 | 4,195 | 3 | 12 | Not Available | 15 |
| **United Kingdom** | Not Available | Not Available | Not Available | Not Available | Not Available | Not Available |
| **United States** | 33,571 | 64,739 | Not Available | Not Available | Not Available | Not Available |
| **Uruguay** | Not Available | Not Available | Not Available | Not Available | Not Available | Not Available |
| **Uzbekistan** | 511,444 | 71,828 | 796 | 1,988 | 12,791 | 2,784 |
| **Vanuatu** | 7,515 | 551 | 10 | 10 | 44 | 20 |
| **Venezuela** | 796,878 | 44,684 | Not Available | Not Available | Not Available | Not Available |
| **Viet Nam** | Not Available | Not Available | 1,198 | 2,374 | 14,290 | 3,572 |
| **Virgin Islands (U.S.)** | Not Available | Not Available | Not Available | Not Available | Not Available | Not Available |
| **West Bank and Gaza** | Not Available | Not Available | Not Available | Not Available | Not Available | Not Available |
| **Yemen** | 2,107,853 | 132,327 | 1,335 | 2,828 | 2,174 | 4,163 |
| **Zambia** | 1,097,729 | 28,412 | 1,389 | 2,054 | 5,091 | 3,443 |
| **Zimbabwe** | 1,074,156 | 26,271 | 1,910 | 2,528 | 3,802 | 4,438 |

**WEB APPENDIX TABLE 4.** Women’s health outcomes attributable to not breastfeeding according to recommendations by country.

|  | Number of maternal deaths due to breast cancer preventable with breastfeeding | Number of cases of breast cancer (incidence) preventable with breastfeeding | Number of maternal deaths due to ovarian cancer preventable with breastfeeding | Number of cases of ovarian cancer (incidence) preventable with breastfeeding | Number of maternal deaths due to Type II diabetes preventable with breastfeeding | Incidence of maternal type II diabetes preventable with breastfeeding | Total number of maternal deaths attributable to not breastfeeding |
| --- | --- | --- | --- | --- | --- | --- | --- |
| **Afghanistan** | 127 | 660 | 28 | 29 | 220 | 3,972 | 375 |
| **Albania** | 20 | 121 | 9 | 14 | 7 | 275 | 36 |
| **Algeria** | 134 | 613 | 58 | 81 | 683 | 10,892 | 875 |
| **American Samoa** | Not Available | Not Available | Not Available | Not Available | Not Available | Not Available | Not Available |
| **Andorra** | Not Available | Not Available | Not Available | Not Available | Not Available | Not Available | Not Available |
| **Angola** | 88 | 413 | 47 | 52 | 254 | 2,248 | 389 |
| **Antigua and Barbuda** | Not Available | Not Available | Not Available | Not Available | Not Available | Not Available | Not Available |
| **Argentina** | 673 | 2,340 | 257 | 345 | 595 | 5,842 | 1,525 |
| **Armenia** | 60 | 241 | 24 | 35 | 106 | 1,041 | 189 |
| **Aruba** | Not Available | Not Available | Not Available | Not Available | Not Available | Not Available | Not Available |
| **Australia** | Not Available | Not Available | Not Available | Not Available | Not Available | Not Available | Not Available |
| **Austria** | 247 | 900 | 147 | 224 | 210 | 1,771 | 604 |
| **Azerbaijan** | 74 | 332 | 37 | 71 | 114 | 2,475 | 225 |
| **Bahamas** | Not Available | Not Available | Not Available | Not Available | Not Available | Not Available | Not Available |
| **Bahrain** | Not Available | Not Available | Not Available | Not Available | Not Available | Not Available | Not Available |
| **Bangladesh** | 101 | 479 | 36 | 50 | 473 | 4,576 | 610 |
| **Barbados** | Not Available | Not Available | Not Available | Not Available | Not Available | Not Available | Not Available |
| **Belarus** | 197 | 662 | 147 | 326 | 50 | 1,813 | 395 |
| **Belgium** | Not Available | Not Available | Not Available | Not Available | Not Available | Not Available | Not Available |
| **Belize** | 1 | 7 | 0 | 1 | 8 | 69 | 10 |
| **Benin** | 29 | 134 | 13 | 13 | 73 | 543 | 115 |
| **Bermuda** | Not Available | Not Available | Not Available | Not Available | Not Available | Not Available | Not Available |
| **Bhutan** | 1 | 5 | 1 | 1 | 5 | 83 | 7 |
| **Bolivia** | 43 | 185 | 21 | 27 | 95 | 1,136 | 159 |
| **Bosnia and Herzegovina** | 53 | 271 | 32 | 50 | 140 | 1,126 | 225 |
| **Botswana** | 20 | 88 | 8 | 12 | 100 | 762 | 128 |
| **Brazil** | 1,757 | 8,186 | 824 | 1,246 | 4,195 | 41,538 | 6,777 |
| **British Virgin Islands** | Not Available | Not Available | Not Available | Not Available | Not Available | Not Available | Not Available |
| **Brunei** | Not Available | Not Available | Not Available | Not Available | Not Available | Not Available | Not Available |
| **Bulgaria** | Not Available | Not Available | Not Available | Not Available | Not Available | Not Available | Not Available |
| **Burkina Faso** | 20 | 98 | 7 | 7 | 29 | 314 | 56 |
| **Burundi** | 10 | 45 | 7 | 7 | 12 | 172 | 28 |
| **Cabo Verde** | 3 | 11 | 1 | 1 | 9 | 92 | 12 |
| **Cambodia** | 76 | 361 | 41 | 53 | 138 | 1,876 | 256 |
| **Cameroon** | 89 | 403 | 40 | 48 | 319 | 2,313 | 448 |
| **Canada** | Not Available | Not Available | Not Available | Not Available | Not Available | Not Available | Not Available |
| **Cayman Islands** | Not Available | Not Available | Not Available | Not Available | Not Available | Not Available | Not Available |
| **Central African Republic** | 28 | 129 | 13 | 12 | 56 | 589 | 97 |
| **Chad** | 17 | 77 | 8 | 8 | 51 | 514 | 75 |
| **Channel Islands** | Not Available | Not Available | Not Available | Not Available | Not Available | Not Available | Not Available |
| **Chile** | Not Available | Not Available | Not Available | Not Available | Not Available | Not Available | Not Available |
| **China** | 8,358 | 37,995 | 3,911 | 6,658 | 10,267 | 271,447 | 22,537 |
| **Colombia** | 289 | 1,199 | 167 | 282 | 416 | 7,761 | 872 |
| **Comoros** | 2 | 8 | 1 | 2 | 3 | 41 | 6 |
| **Congo** | 36 | 160 | 20 | 23 | 101 | 879 | 157 |
| **Costa Rica** | 39 | 147 | 17 | 30 | 31 | 728 | 87 |
| **Côte d'Ivoire** | 53 | 245 | 28 | 34 | 168 | 1,663 | 249 |
| **Croatia** | Not Available | Not Available | Not Available | Not Available | Not Available | Not Available | Not Available |
| **Cuba** | 182 | 564 | 64 | 114 | 155 | 2,309 | 401 |
| **Curaçao** | Not Available | Not Available | Not Available | Not Available | Not Available | Not Available | Not Available |
| **Cyprus** | Not Available | Not Available | Not Available | Not Available | Not Available | Not Available | Not Available |
| **Czech Republic** | Not Available | Not Available | Not Available | Not Available | Not Available | Not Available | Not Available |
| **Democratic People's Republic of Korea** | 179 | 799 | 93 | 140 | 143 | 4,206 | 416 |
| **Denmark** | Not Available | Not Available | Not Available | Not Available | Not Available | Not Available | Not Available |
| **Djibouti** | 5 | 23 | 4 | 5 | 14 | 112 | 23 |
| **Dominica** | Not Available | Not Available | Not Available | Not Available | Not Available | Not Available | Not Available |
| **Dominican Republic** | 77 | 325 | 21 | 32 | 194 | 1,567 | 293 |
| **Democratic Republic of the Congo** | 119 | 541 | 62 | 60 | 160 | 3,340 | 341 |
| **Ecuador** | 85 | 374 | 55 | 84 | 342 | 3,564 | 482 |
| **Egypt** | 464 | 2,139 | 124 | 176 | 1,394 | 27,358 | 1,982 |
| **El Salvador** | 19 | 81 | 11 | 19 | 80 | 907 | 110 |
| **Equatorial Guinea** | 6 | 28 | 4 | 4 | 23 | 208 | 32 |
| **Eritrea** | 8 | 38 | 6 | 6 | 12 | 144 | 25 |
| **Estonia** | Not Available | Not Available | Not Available | Not Available | Not Available | Not Available | Not Available |
| **Ethiopia** | 137 | 631 | 84 | 92 | 237 | 2,377 | 458 |
| **Faroe Islands** | Not Available | Not Available | Not Available | Not Available | Not Available | Not Available | Not Available |
| **Fiji** | Not Available | Not Available | Not Available | Not Available | Not Available | Not Available | Not Available |
| **Finland** | Not Available | Not Available | Not Available | Not Available | Not Available | Not Available | Not Available |
| **France** | Not Available | Not Available | Not Available | Not Available | Not Available | Not Available | Not Available |
| **French Polynesia** | Not Available | Not Available | Not Available | Not Available | Not Available | Not Available | Not Available |
| **Gabon** | 15 | 61 | 9 | 12 | 56 | 411 | 80 |
| **Gambia** | 3 | 15 | 2 | 2 | 9 | 104 | 14 |
| **Georgia** | 76 | 287 | 35 | 55 | 78 | 1,020 | 190 |
| **Germany** | Not Available | Not Available | Not Available | Not Available | Not Available | Not Available | Not Available |
| **Ghana** | 91 | 418 | 35 | 43 | 176 | 1,834 | 302 |
| **Gibraltar** | Not Available | Not Available | Not Available | Not Available | Not Available | Not Available | Not Available |
| **Greece** | Not Available | Not Available | Not Available | Not Available | Not Available | Not Available | Not Available |
| **Greenland** | Not Available | Not Available | Not Available | Not Available | Not Available | Not Available | Not Available |
| **Grenada** | Not Available | Not Available | Not Available | Not Available | Not Available | Not Available | Not Available |
| **Guam** | Not Available | Not Available | Not Available | Not Available | Not Available | Not Available | Not Available |
| **Guatemala** | 29 | 128 | 15 | 22 | 212 | 2,708 | 256 |
| **Guinea** | 16 | 73 | 12 | 13 | 48 | 416 | 75 |
| **Guinea-Bissau** | 6 | 29 | 2 | 2 | 18 | 125 | 27 |
| **Guyana** | 5 | 24 | 3 | 6 | 28 | 204 | 37 |
| **Haiti** | 100 | 463 | 27 | 31 | 280 | 1,859 | 407 |
| **Honduras** | 25 | 109 | 14 | 22 | 84 | 1,109 | 123 |
| **Hong Kong** | Not Available | Not Available | Not Available | Not Available | Not Available | Not Available | Not Available |
| **Hungary** | Not Available | Not Available | Not Available | Not Available | Not Available | Not Available | Not Available |
| **Iceland** | Not Available | Not Available | Not Available | Not Available | Not Available | Not Available | Not Available |
| **India** | 2,129 | 7,976 | 1,273 | 1,748 | 8,002 | 87,855 | 11,404 |
| **Indonesia** | 947 | 4,524 | 548 | 909 | 3,675 | 43,384 | 5,170 |
| **Iran** | 177 | 810 | 76 | 141 | 398 | 13,003 | 652 |
| **Iraq** | 281 | 1,378 | 71 | 103 | 624 | 12,226 | 975 |
| **Ireland** | Not Available | Not Available | Not Available | Not Available | Not Available | Not Available | Not Available |
| **Isle of Man** | Not Available | Not Available | Not Available | Not Available | Not Available | Not Available | Not Available |
| **Israel** | Not Available | Not Available | Not Available | Not Available | Not Available | Not Available | Not Available |
| **Italy** | Not Available | Not Available | Not Available | Not Available | Not Available | Not Available | Not Available |
| **Jamaica** | 38 | 146 | 15 | 24 | 187 | 837 | 240 |
| **Japan** | Not Available | Not Available | Not Available | Not Available | Not Available | Not Available | Not Available |
| **Jordan** | 34 | 159 | 13 | 21 | 83 | 2,494 | 130 |
| **Kazakhstan** | 188 | 760 | 130 | 214 | 115 | 3,164 | 432 |
| **Kenya** | 71 | 318 | 87 | 96 | 113 | 2,065 | 271 |
| **Kiribati** | 0 | 1 | 0 | 0 | 1 | 13 | 2 |
| **Kosovo** | Not Available | Not Available | Not Available | Not Available | Not Available | Not Available | Not Available |
| **Kuwait** | 16 | 82 | 5 | 13 | 12 | 1,137 | 34 |
| **Kyrgyzstan** | 30 | 124 | 20 | 31 | 19 | 663 | 69 |
| **Laos** | 28 | 131 | 14 | 19 | 75 | 1,167 | 117 |
| **Latvia** | Not Available | Not Available | Not Available | Not Available | Not Available | Not Available | Not Available |
| **Lebanon** | 68 | 277 | 41 | 62 | 88 | 2,019 | 197 |
| **Lesotho** | 15 | 58 | 6 | 7 | 74 | 453 | 95 |
| **Liberia** | 11 | 51 | 5 | 5 | 35 | 309 | 51 |
| **Libya** | Not Available | Not Available | Not Available | Not Available | Not Available | Not Available | Not Available |
| **Liechtenstein** | Not Available | Not Available | Not Available | Not Available | Not Available | Not Available | Not Available |
| **Lithuania** | Not Available | Not Available | Not Available | Not Available | Not Available | Not Available | Not Available |
| **Luxembourg** | Not Available | Not Available | Not Available | Not Available | Not Available | Not Available | Not Available |
| **Macao SAR, China** | Not Available | Not Available | Not Available | Not Available | Not Available | Not Available | Not Available |
| **Madagascar** | 16 | 74 | 13 | 15 | 23 | 370 | 52 |
| **Malawi** | 11 | 51 | 12 | 14 | 41 | 415 | 64 |
| **Malaysia** | Not Available | Not Available | Not Available | Not Available | Not Available | Not Available | Not Available |
| **Maldives** | 0 | 2 | 0 | 0 | 1 | 21 | 1 |
| **Mali** | 21 | 100 | 12 | 12 | 78 | 827 | 111 |
| **Malta** | Not Available | Not Available | Not Available | Not Available | Not Available | Not Available | Not Available |
| **Marshall Islands** | 0 | 1 | 0 | 0 | 2 | 36 | 3 |
| **Mauritania** | 11 | 52 | 5 | 6 | 44 | 337 | 60 |
| **Mauritius** | 21 | 87 | 9 | 16 | 142 | 946 | 173 |
| **Mexico** | 729 | 3,115 | 470 | 894 | 3,995 | 40,513 | 5,195 |
| **Micronesia** | Not Available | Not Available | Not Available | Not Available | Not Available | Not Available | Not Available |
| **Moldova** | Not Available | Not Available | Not Available | Not Available | Not Available | Not Available | Not Available |
| **Monaco** | Not Available | Not Available | Not Available | Not Available | Not Available | Not Available | Not Available |
| **Mongolia** | 6 | 28 | 5 | 8 | 3 | 235 | 14 |
| **Montenegro** | 11 | 57 | 5 | 8 | 10 | 154 | 26 |
| **Morocco** | 290 | 1,350 | 96 | 117 | 1,276 | 13,494 | 1,662 |
| **Mozambique** | 70 | 328 | 45 | 48 | 148 | 1,384 | 263 |
| **Myanmar** | 416 | 1,905 | 197 | 261 | 415 | 6,715 | 1,028 |
| **Namibia** | 4 | 21 | 3 | 4 | 42 | 533 | 49 |
| **Nauru** | Not Available | Not Available | Not Available | Not Available | Not Available | Not Available | Not Available |
| **Nepal** | 16 | 70 | 8 | 9 | 56 | 619 | 80 |
| **Netherlands** | Not Available | Not Available | Not Available | Not Available | Not Available | Not Available | Not Available |
| **New Caledonia** | Not Available | Not Available | Not Available | Not Available | Not Available | Not Available | Not Available |
| **New Zealand** | Not Available | Not Available | Not Available | Not Available | Not Available | Not Available | Not Available |
| **Nicaragua** | 16 | 73 | 8 | 14 | 72 | 816 | 97 |
| **Niger** | 24 | 113 | 12 | 11 | 64 | 900 | 100 |
| **Nigeria** | 355 | 1,832 | 182 | 232 | 974 | 10,471 | 1,511 |
| **Northern Mariana Islands** | Not Available | Not Available | Not Available | Not Available | Not Available | Not Available | Not Available |
| **Norway** | 101 | 521 | 89 | 146 | 53 | 964 | 243 |
| **Oman** | 5 | 22 | 3 | 5 | 26 | 745 | 33 |
| **Pakistan** | 1,044 | 4,753 | 318 | 577 | 1,916 | 22,978 | 3,278 |
| **Palau** | Not Available | Not Available | Not Available | Not Available | Not Available | Not Available | Not Available |
| **Panama** | 24 | 91 | 12 | 23 | 67 | 805 | 102 |
| **Papua New Guinea** | 20 | 93 | 5 | 7 | 117 | 1,033 | 143 |
| **Paraguay** | 57 | 245 | 23 | 33 | 196 | 1,615 | 277 |
| **Peru** | 77 | 368 | 51 | 84 | 139 | 1,661 | 267 |
| **Philippines** | 626 | 3,238 | 274 | 467 | 1,013 | 20,693 | 1,912 |
| **Poland** | Not Available | Not Available | Not Available | Not Available | Not Available | Not Available | Not Available |
| **Portugal** | Not Available | Not Available | Not Available | Not Available | Not Available | Not Available | Not Available |
| **Puerto Rico** | Not Available | Not Available | Not Available | Not Available | Not Available | Not Available | Not Available |
| **Qatar** | 6 | 27 | 2 | 5 | 10 | 495 | 18 |
| **Republic of Korea** | Not Available | Not Available | Not Available | Not Available | Not Available | Not Available | Not Available |
| **Romania** | Not Available | Not Available | Not Available | Not Available | Not Available | Not Available | Not Available |
| **Russia** | Not Available | Not Available | Not Available | Not Available | Not Available | Not Available | Not Available |
| **Rwanda** | 8 | 38 | 6 | 7 | 11 | 157 | 26 |
| **Saint Kitts and Nevis** | Not Available | Not Available | Not Available | Not Available | Not Available | Not Available | Not Available |
| **Saint Lucia** | Not Available | Not Available | Not Available | Not Available | Not Available | Not Available | Not Available |
| **Saint Martin** | Not Available | Not Available | Not Available | Not Available | Not Available | Not Available | Not Available |
| **Saint Vincent and the Grenadines** | Not Available | Not Available | Not Available | Not Available | Not Available | Not Available | Not Available |
| **Samoa** | 0 | 1 | 0 | 0 | 3 | 23 | 4 |
| **San Marino** | Not Available | Not Available | Not Available | Not Available | Not Available | Not Available | Not Available |
| **Sao Tome and Principe** | 1 | 4 | 0 | 0 | 1 | 15 | 2 |
| **Saudi Arabia** | 56 | 253 | 25 | 43 | 61 | 5,157 | 141 |
| **Senegal** | 38 | 177 | 16 | 17 | 108 | 1,098 | 161 |
| **Serbia** | 232 | 751 | 119 | 251 | 241 | 2,761 | 592 |
| **Seychelles** | Not Available | Not Available | Not Available | Not Available | Not Available | Not Available | Not Available |
| **Sierra Leone** | 18 | 84 | 7 | 7 | 46 | 425 | 70 |
| **Singapore** | Not Available | Not Available | Not Available | Not Available | Not Available | Not Available | Not Available |
| **Sint Maarten (Dutch part)** | Not Available | Not Available | Not Available | Not Available | Not Available | Not Available | Not Available |
| **Slovakia** | Not Available | Not Available | Not Available | Not Available | Not Available | Not Available | Not Available |
| **Slovenia** | Not Available | Not Available | Not Available | Not Available | Not Available | Not Available | Not Available |
| **Solomon Islands** | 2 | 8 | 0 | 1 | 12 | 99 | 14 |
| **Somalia** | 36 | 170 | 26 | 25 | 81 | 793 | 143 |
| **South Africa** | 618 | 2,327 | 269 | 363 | 2,928 | 20,417 | 3,815 |
| **South Sudan** | 41 | 187 | 28 | 29 | 90 | 944 | 159 |
| **Spain** | Not Available | Not Available | Not Available | Not Available | Not Available | Not Available | Not Available |
| **Sri Lanka** | 25 | 107 | 13 | 21 | 119 | 1,516 | 157 |
| **Sudan** | 111 | 538 | 37 | 42 | 207 | 4,892 | 355 |
| **Suriname** | 5 | 19 | 3 | 4 | 17 | 147 | 25 |
| **Swaziland** | 10 | 40 | 4 | 6 | 59 | 429 | 73 |
| **Sweden** | 226 | 1,251 | 180 | 249 | 145 | 2,667 | 551 |
| **Switzerland** | Not Available | Not Available | Not Available | Not Available | Not Available | Not Available | Not Available |
| **Syria** | 53 | 245 | 21 | 30 | 69 | 3,166 | 144 |
| **Taiwan** | Not Available | Not Available | Not Available | Not Available | Not Available | Not Available | Not Available |
| **Tajikistan** | 15 | 71 | 10 | 15 | 22 | 632 | 47 |
| **Tanzania** | 123 | 564 | 109 | 125 | 326 | 2,680 | 557 |
| **Thailand** | 746 | 3,247 | 434 | 701 | 1,841 | 19,897 | 3,021 |
| **The former Yugoslav Republic of Macedonia** | 35 | 184 | 18 | 28 | 50 | 600 | 103 |
| **Timor-Leste** | 3 | 16 | 2 | 3 | 7 | 129 | 13 |
| **Togo** | 13 | 59 | 6 | 6 | 25 | 286 | 44 |
| **Tonga** | 2 | 6 | 0 | 0 | 6 | 37 | 8 |
| **Trinidad and Tobago** | 20 | 64 | 13 | 19 | 115 | 668 | 148 |
| **Tunisia** | 97 | 429 | 32 | 44 | 343 | 3,731 | 472 |
| **Turkey** | 344 | 1,543 | 231 | 342 | 879 | 18,777 | 1,454 |
| **Turkmenistan** | 32 | 146 | 15 | 32 | 39 | 984 | 86 |
| **Turks and Caicos Islands** | Not Available | Not Available | Not Available | Not Available | Not Available | Not Available | Not Available |
| **Tuvalu** | Not Available | Not Available | Not Available | Not Available | Not Available | Not Available | Not Available |
| **Uganda** | 60 | 274 | 84 | 100 | 146 | 1,641 | 290 |
| **Ukraine** | 951 | 2,269 | 594 | 1,066 | 225 | 7,549 | 1,769 |
| **United Arab Emirates** | 26 | 129 | 8 | 24 | 22 | 2,114 | 56 |
| **United Kingdom** | Not Available | Not Available | Not Available | Not Available | Not Available | Not Available | Not Available |
| **United States** | Not Available | Not Available | Not Available | Not Available | Not Available | Not Available | Not Available |
| **Uruguay** | Not Available | Not Available | Not Available | Not Available | Not Available | Not Available | Not Available |
| **Uzbekistan** | 115 | 525 | 35 | 58 | 179 | 3,641 | 329 |
| **Vanuatu** | 2 | 7 | 0 | 1 | 9 | 62 | 11 |
| **Venezuela** | Not Available | Not Available | Not Available | Not Available | Not Available | Not Available | Not Available |
| **Viet Nam** | 486 | 2,032 | 396 | 677 | 2,236 | 17,952 | 3,118 |
| **Virgin Islands (U.S.)** | Not Available | Not Available | Not Available | Not Available | Not Available | Not Available | Not Available |
| **West Bank and Gaza** | Not Available | Not Available | Not Available | Not Available | Not Available | Not Available | Not Available |
| **Yemen** | 88 | 444 | 28 | 31 | 158 | 3,298 | 273 |
| **Zambia** | 56 | 259 | 42 | 51 | 100 | 888 | 198 |
| **Zimbabwe** | 72 | 256 | 36 | 46 | 206 | 2,243 | 315 |

**WEB APPENDIX TABLE 5.** Annual health system and formula feeding cost attributable to not recommended breastfeeding practices by country.

|  | **Cost of avoidable health care treatment of type 2 diabetes morbidity in mothers' attributable to not breastfeeding** (US$). | **Cost of avoidable health care treatment of childhood diarrhea attributable to not breastfeeding** (US$). | **Cost of avoidable health care treatment of childhood ARI/pneumonia attributable to not breastfeeding** (US$). | **Total health system cost of not breastfeeding** (US$). | **Cost of formula feeding children 0-23 months as percent (%) of nominal wages** |
| --- | --- | --- | --- | --- | --- |
| **Afghanistan** | 218,590 | 2,639,529 | 287,379 | 3,145,498 | Not Available |
| **Albania** | 36,216 | 66,999 | 120,023 | 223,237 | Not Available |
| **Algeria** | 2,106,146 | 9,337,817 | 3,219,861 | 14,663,825 | Not Available |
| **American Samoa** | Not Available | Not Available | Not Available | Not Available | Not Available |
| **Andorra** | Not Available | Not Available | Not Available | Not Available | Not Available |
| **Angola** | 745,899 | 16,593,048 | 3,809,334 | 21,148,281 | Not Available |
| **Antigua and Barbuda** | Not Available | Not Available | Not Available | Not Available | Not Available |
| **Argentina** | 3,111,690 | 4,583,709 | 15,486,902 | 23,182,302 | 3.8 |
| **Armenia** | 88,445 | 208,115 | 73,997 | 370,558 | 10.0 |
| **Aruba** | Not Available | Not Available | Not Available | Not Available | 2.2 |
| **Australia** | Not Available | Not Available | Not Available | Not Available | Not Available |
| **Austria** | 4,064,682 | Not Available | Not Available | 4,064,682 | 1.0 |
| **Azerbaijan** | 646,115 | 1,501,809 | 612,595 | 2,760,520 | 14.0 |
| **Bahamas** | Not Available | Not Available | Not Available | Not Available | Not Available |
| **Bahrain** | Not Available | 534,873 | 265,346 | 800,219 | 5.9 |
| **Bangladesh** | 120,624 | 4,141,156 | 611,618 | 4,873,398 | Not Available |
| **Barbados** | Not Available | 0 | 31,753 | 31,753 | Not Available |
| **Belarus** | 458,779 | 352,379 | 3,031,849 | 3,843,007 | 0.0 |
| **Belgium** | Not Available | 391,025 | 1,140,764 | 1,531,789 | 0.8 |
| **Belize** | 9,477 | 8,424 | 23,457 | 41,358 | 2.0 |
| **Benin** | 28,349 | 513,381 | 39,058 | 580,788 | Not Available |
| **Bermuda** | Not Available | Not Available | Not Available | Not Available | Not Available |
| **Bhutan** | 6,050 | 42,745 | 22,314 | 71,109 | Not Available |
| **Bolivia** | 127,080 | 1,916,275 | 308,615 | 2,351,971 | 2.7 |
| **Bosnia and Herzegovina** | 254,337 | 7,912 | 128,578 | 390,828 | 2.7 |
| **Botswana** | 298,339 | 912,215 | 54,401 | 1,264,955 | 4.3 |
| **Brazil** | 25,484,956 | 12,425,838 | 4,303,455 | 42,214,249 | 4.9 |
| **British Virgin Islands** | Not Available | Not Available | Not Available | Not Available | Not Available |
| **Brunei** | Not Available | Not Available | Not Available | Not Available | Not Available |
| **Bulgaria** | Not Available | Not Available | Not Available | Not Available | 6.2 |
| **Burkina Faso** | 20,384 | 1,474,069 | 105,847 | 1,600,301 | Not Available |
| **Burundi** | 5,136 | 589,629 | 37,289 | 632,055 | Not Available |
| **Cabo Verde** | 15,124 | Not Available | Not Available | 15,124 | Not Available |
| **Cambodia** | 158,671 | 729,751 | 116,551 | 1,004,974 | Not Available |
| **Cameroon** | 172,703 | 1,616,793 | 159,729 | 1,949,226 | Not Available |
| **Canada** | Not Available | Not Available | Not Available | Not Available | 4.3 |
| **Cayman Islands** | Not Available | Not Available | Not Available | Not Available | Not Available |
| **Central African Republic** | 9,754 | 193,214 | 21,925 | 224,893 | Not Available |
| **Chad** | 26,980 | 346,908 | 102,107 | 475,995 | Not Available |
| **Channel Islands** | Not Available | Not Available | Not Available | Not Available | Not Available |
| **Chile** | Not Available | Not Available | Not Available | Not Available | Not Available |
| **China** | 66,695,355 | 88,226,076 | 41,432,577 | 196,354,008 | 4.1 |
| **Colombia** | 2,400,997 | 13,346,417 | 2,643,364 | 18,390,779 | 14.8 |
| **Comoros** | Not Available | 60,220 | 5,958 | 66,177 | Not Available |
| **Congo** | 126,045 | 535,749 | 55,733 | 717,527 | 7.0 |
| **Costa Rica** | 414,019 | 96,977 | 435,291 | 946,287 | 4.6 |
| **Côte d'Ivoire** | 169,182 | Not Available | Not Available | 169,182 | Not Available |
| **Croatia** | Not Available | 102,358 | 236,266 | 338,624 | 3.2 |
| **Cuba** | 667,735 | 1,071,136 | 391,589 | 2,130,460 | Not Available |
| **Curaçao** | Not Available | Not Available | Not Available | Not Available | Not Available |
| **Cyprus** | Not Available | Not Available | Not Available | Not Available | 1.6 |
| **Czech Republic** | Not Available | Not Available | Not Available | Not Available | 2.3 |
| **Democratic People's Republic of Korea** | Not Available | Not Available | Not Available | Not Available | Not Available |
| **Denmark** | Not Available | Not Available | Not Available | Not Available | Not Available |
| **Djibouti** | 14,983 | 63,332 | 23,674 | 101,989 | Not Available |
| **Dominica** | Not Available | Not Available | Not Available | Not Available | Not Available |
| **Dominican Republic** | 307,563 | 3,848,946 | 1,201,152 | 5,357,661 | 5.4 |
| **Democratic Republic of the Congo** | 72,692 | 3,834,224 | 252,710 | 4,159,626 | Not Available |
| **Ecuador** | 923,922 | 3,036,214 | 698,333 | 4,658,469 | 2.0 |
| **Egypt** | 2,466,355 | 22,251,342 | 5,138,687 | 29,856,384 | 13.0 |
| **El Salvador** | 142,725 | 747,733 | 217,659 | 1,108,117 | Not Available |
| **Equatorial Guinea** | 140,906 | 2,302,841 | 473,742 | 2,917,488 | Not Available |
| **Eritrea** | 3,236 | 206,962 | 27,499 | 237,697 | Not Available |
| **Estonia** | Not Available | Not Available | Not Available | Not Available | 28.0 |
| **Ethiopia** | 78,708 | 1,543,405 | 141,666 | 1,763,779 | 16.4 |
| **Faroe Islands** | Not Available | Not Available | Not Available | Not Available | Not Available |
| **Fiji** | Not Available | 87,755 | 59,444 | 147,199 | Not Available |
| **Finland** | Not Available | Not Available | Not Available | Not Available | 0.6 |
| **France** | Not Available | Not Available | Not Available | Not Available | 0.8 |
| **French Polynesia** | Not Available | Not Available | Not Available | Not Available | Not Available |
| **Gabon** | 182,569 | 1,025,774 | 306,950 | 1,515,294 | Not Available |
| **Gambia** | 4,298 | Not Available | Not Available | 4,298 | Not Available |
| **Georgia** | 183,585 | 145,385 | 71,305 | 400,275 | 3.3 |
| **Germany** | Not Available | Not Available | Not Available | Not Available | 0.6 |
| **Ghana** | 236,331 | 5,153,199 | 497,805 | 5,887,335 | Not Available |
| **Gibraltar** | Not Available | Not Available | Not Available | Not Available | 12.8 |
| **Greece** | Not Available | Not Available | Not Available | Not Available | 2.7 |
| **Greenland** | Not Available | Not Available | Not Available | Not Available | Not Available |
| **Grenada** | Not Available | 22,365 | 9,739 | 32,104 | Not Available |
| **Guam** | Not Available | Not Available | Not Available | Not Available | Not Available |
| **Guatemala** | 397,870 | 4,152,923 | 710,231 | 5,261,024 | 4.5 |
| **Guinea** | 14,050 | 883,698 | 81,573 | 979,320 | Not Available |
| **Guinea-Bissau** | Not Available | 66,353 | 3,465 | 69,818 | Not Available |
| **Guyana** | 27,233 | 84,777 | 19,365 | 131,376 | 3.2 |
| **Haiti** | 107,844 | 756,010 | 94,251 | 958,105 | Not Available |
| **Honduras** | 144,674 | 1,586,451 | 246,804 | 1,977,929 | Not Available |
| **Hong Kong** | Not Available | Not Available | Not Available | Not Available | Not Available |
| **Hungary** | Not Available | Not Available | Not Available | Not Available | 2.2 |
| **Iceland** | Not Available | Not Available | Not Available | Not Available | 0.3 |
| **India** | 4,345,470 | 74,295,217 | 27,414,080 | 106,054,767 | 19.4 |
| **Indonesia** | 3,915,601 | 75,873,575 | 5,236,816 | 85,025,991 | 9.9 |
| **Iran** | 3,380,161 | 20,470,035 | 9,538,217 | 33,388,413 | Not Available |
| **Iraq** | 2,766,795 | 2,526,891 | 2,662,747 | 7,956,434 | Not Available |
| **Ireland** | Not Available | Not Available | Not Available | Not Available | 0.7 |
| **Isle of Man** | Not Available | Not Available | Not Available | Not Available | Not Available |
| **Israel** | Not Available | Not Available | Not Available | Not Available | 1.5 |
| **Italy** | Not Available | Not Available | Not Available | Not Available | Not Available |
| **Jamaica** | 117,434 | 131,958 | 359,489 | 608,880 | 29.4 |
| **Japan** | Not Available | Not Available | Not Available | Not Available | 1.0 |
| **Jordan** | 576,625 | 2,263,549 | 632,944 | 3,473,118 | Not Available |
| **Kazakhstan** | 1,146,458 | 2,960,414 | 3,685,861 | 7,792,732 | 2.1 |
| **Kenya** | 102,438 | 5,242,075 | 674,769 | 6,019,283 | Not Available |
| **Kiribati** | 1,720 | 4,835 | 2,936 | 9,491 | Not Available |
| **Kosovo** | Not Available | Not Available | Not Available | Not Available | Not Available |
| **Kuwait** | 815,639 | 2,533,642 | 1,801,407 | 5,150,689 | Not Available |
| **Kyrgyzstan** | 50,081 | 303,461 | 122,067 | 475,610 | 6.1 |
| **Laos** | 43,818 | 217,140 | 51,391 | 312,348 | Not Available |
| **Latvia** | Not Available | Not Available | Not Available | Not Available | 1.4 |
| **Lebanon** | 723,437 | 967,989 | 504,082 | 2,195,508 | Not Available |
| **Lesotho** | 61,582 | 194,175 | 17,730 | 273,487 | Not Available |
| **Liberia** | 18,637 | 172,794 | 12,074 | 203,505 | Not Available |
| **Libya** | Not Available | Not Available | Not Available | Not Available | Not Available |
| **Liechtenstein** | Not Available | Not Available | Not Available | Not Available | Not Available |
| **Lithuania** | Not Available | Not Available | Not Available | Not Available | 8.6 |
| **Luxembourg** | Not Available | Not Available | Not Available | Not Available | 0.7 |
| **Macao SAR, China** | Not Available | Not Available | Not Available | Not Available | Not Available |
| **Madagascar** | 9,916 | 1,238,005 | 153,659 | 1,401,579 | Not Available |
| **Malawi** | 15,556 | 1,936,717 | 198,725 | 2,150,997 | Not Available |
| **Malaysia** | Not Available | 4,179,314 | 1,317,360 | 5,496,675 | 4.1 |
| **Maldives** | 12,251 | 96,559 | 3,229 | 112,039 | 2.3 |
| **Mali** | 63,574 | 1,004,980 | 67,411 | 1,135,965 | Not Available |
| **Malta** | Not Available | Not Available | Not Available | Not Available | 0.6 |
| **Marshall Islands** | 15,647 | 4,522 | 3,069 | 23,239 | Not Available |
| **Mauritania** | 18,331 | 286,488 | 34,165 | 338,984 | Not Available |
| **Mauritius** | 243,154 | 95,827 | 36,595 | 375,576 | 3.0 |
| **Mexico** | 12,510,348 | 25,223,420 | 9,493,647 | 47,227,415 | 8.0 |
| **Micronesia** | Not Available | Not Available | Not Available | Not Available | Not Available |
| **Moldova** | Not Available | Not Available | Not Available | Not Available | 5.0 |
| **Monaco** | Not Available | Not Available | Not Available | Not Available | Not Available |
| **Mongolia** | 45,450 | 298,876 | 177,635 | 521,962 | 5.9 |
| **Montenegro** | 0 | 19,295 | 45,683 | 64,979 | 1.5 |
| **Morocco** | 1,561,258 | 2,051,986 | 1,112,099 | 4,725,343 | Not Available |
| **Mozambique** | 79,364 | 1,477,418 | 123,461 | 1,680,244 | Not Available |
| **Myanmar** | 80,649 | 1,518,059 | 232,655 | 1,831,363 | Not Available |
| **Namibia** | 245,343 | 1,573,059 | 166,014 | 1,984,416 | Not Available |
| **Nauru** | Not Available | Not Available | Not Available | Not Available | Not Available |
| **Nepal** | 26,345 | 1,334,577 | 120,925 | 1,481,847 | Not Available |
| **Netherlands** | Not Available | Not Available | Not Available | Not Available | 0.5 |
| **New Caledonia** | Not Available | Not Available | Not Available | Not Available | Not Available |
| **New Zealand** | Not Available | Not Available | Not Available | Not Available | 0.8 |
| **Nicaragua** | 78,802 | 555,620 | 115,559 | 749,981 | 4.4 |
| **Niger** | 36,083 | 1,366,988 | 118,655 | 1,521,727 | 2.4 |
| **Nigeria** | 1,349,531 | 18,735,229 | 1,721,566 | 21,806,326 | Not Available |
| **Northern Mariana Islands** | Not Available | Not Available | Not Available | Not Available | Not Available |
| **Norway** | 4,311,758 | Not Available | Not Available | 4,311,758 | 0.6 |
| **Oman** | 268,016 | 2,840,724 | 671,193 | 3,779,933 | Not Available |
| **Pakistan** | 588,678 | 45,236,102 | 4,983,801 | 50,808,580 | 18.8 |
| **Palau** | Not Available | Not Available | Not Available | Not Available | Not Available |
| **Panama** | 355,190 | 1,254,315 | 541,075 | 2,150,580 | 4.6 |
| **Papua New Guinea** | 63,617 | 304,996 | 206,128 | 574,741 | Not Available |
| **Paraguay** | 371,287 | 718,481 | 571,790 | 1,661,557 | 2.8 |
| **Peru** | 359,060 | 4,478,543 | 1,357,467 | 6,195,070 | 11.4 |
| **Philippines** | 2,218,062 | 9,745,871 | 4,350,735 | 16,314,667 | Not Available |
| **Poland** | Not Available | Not Available | Not Available | Not Available | 2.2 |
| **Portugal** | Not Available | Not Available | Not Available | Not Available | 3.2 |
| **Puerto Rico** | Not Available | Not Available | Not Available | Not Available | Not Available |
| **Qatar** | 504,606 | 1,970,701 | 1,223,785 | 3,699,093 | 1.2 |
| **Republic of Korea** | Not Available | Not Available | Not Available | Not Available | Not Available |
| **Romania** | Not Available | 608,585 | 1,990,748 | 2,599,333 | 5.1 |
| **Russia** | Not Available | Not Available | Not Available | Not Available | 6.1 |
| **Rwanda** | 14,723 | 383,456 | 49,311 | 447,489 | Not Available |
| **Saint Kitts and Nevis** | Not Available | Not Available | Not Available | Not Available | Not Available |
| **Saint Lucia** | Not Available | Not Available | Not Available | Not Available | Not Available |
| **Saint Martin** | Not Available | Not Available | Not Available | Not Available | Not Available |
| **Saint Vincent and the Grenadines** | Not Available | Not Available | Not Available | Not Available | Not Available |
| **Samoa** | 5,697 | 26,683 | 14,557 | 46,937 | 6.0 |
| **San Marino** | Not Available | Not Available | Not Available | Not Available | 0.5 |
| **Sao Tome and Principe** | 2,016 | 44,580 | 4,171 | 50,768 | Not Available |
| **Saudi Arabia** | 2,107,265 | Not Available | Not Available | 2,107,265 | 1.7 |
| **Senegal** | 58,417 | 1,289,313 | 136,775 | 1,484,506 | Not Available |
| **Serbia** | 660,717 | 37,247 | 421,765 | 1,119,730 | 4.2 |
| **Seychelles** | Not Available | Not Available | Not Available | Not Available | 1.8 |
| **Sierra Leone** | 55,898 | 617,821 | 70,559 | 744,277 | Not Available |
| **Singapore** | Not Available | Not Available | Not Available | Not Available | 1.3 |
| **Sint Maarten (Dutch part)** | Not Available | Not Available | Not Available | Not Available | Not Available |
| **Slovakia** | Not Available | Not Available | Not Available | Not Available | Not Available |
| **Slovenia** | Not Available | Not Available | Not Available | Not Available | Not Available |
| **Solomon Islands** | 8,220 | 31,847 | 17,484 | 57,551 | Not Available |
| **Somalia** | 0 | 0 | 0 | 0 | Not Available |
| **South Africa** | 11,463,576 | 22,290,364 | 3,860,262 | 37,614,202 | 6.3 |
| **South Sudan** | 0 | 1,234,104 | 373,001 | 1,607,105 | Not Available |
| **Spain** | Not Available | Not Available | Not Available | Not Available | 1.1 |
| **Sri Lanka** | 112,278 | 596,868 | 148,811 | 857,957 | 21.6 |
| **Sudan** | 401,000 | 22,492,013 | 3,099,799 | 25,992,813 | Not Available |
| **Suriname** | Not Available | 15,733 | 74,832 | 90,565 | Not Available |
| **Swaziland** | 123,508 | 543,406 | 49,051 | 715,965 | Not Available |
| **Sweden** | 6,821,777 | Not Available | Not Available | 6,821,777 | 0.6 |
| **Switzerland** | Not Available | Not Available | Not Available | Not Available | 0.6 |
| **Syria** | 99,585 | 1,082,984 | 493,411 | 1,675,981 | 1.2 |
| **Taiwan** | Not Available | Not Available | Not Available | Not Available | Not Available |
| **Tajikistan** | 33,491 | 974,213 | 185,962 | 1,193,665 | 9.1 |
| **Tanzania** | 183,992 | 3,130,362 | 435,426 | 3,749,779 | 7.9 |
| **Thailand** | 3,602,182 | 684,889 | 1,353,385 | 5,640,456 | 12.4 |
| **The former Yugoslav Republic of Macedonia** | Not Available | Not Available | Not Available | Not Available | Not Available |
| **Timor-Leste** | 7,529 | 74,784 | 6,535 | 88,848 | Not Available |
| **Togo** | 19,953 | 383,581 | 58,310 | 461,844 | Not Available |
| **Tonga** | 6,144 | 8,377 | 4,960 | 19,481 | Not Available |
| **Trinidad and Tobago** | 237,128 | 71,838 | 295,138 | 604,105 | Not Available |
| **Tunisia** | 671,633 | 915,722 | 323,661 | 1,911,015 | Not Available |
| **Turkey** | 6,843,625 | 28,156,666 | 13,163,234 | 48,163,525 | 4.7 |
| **Turkmenistan** | 105,017 | 1,047,143 | 370,881 | 1,523,042 | Not Available |
| **Turks and Caicos Islands** | Not Available | Not Available | Not Available | Not Available | Not Available |
| **Tuvalu** | Not Available | Not Available | Not Available | Not Available | Not Available |
| **Uganda** | 0 | 7,126,613 | 787,369 | 7,913,982 | 70.1 |
| **Ukraine** | 1,165,545 | 325,581 | 3,123,192 | 4,614,318 | 25.8 |
| **United Arab Emirates** | 1,624,727 | 6,660,162 | 1,987,764 | 10,272,653 | 1.9 |
| **United Kingdom** | Not Available | Not Available | Not Available | Not Available | 0.9 |
| **United States** | Not Available | 1,826,502 | 26,443,932 | 28,270,434 | 3.0 |
| **Uruguay** | Not Available | Not Available | Not Available | Not Available | Not Available |
| **Uzbekistan** | 292,873 | 1,606,198 | 1,336,391 | 3,235,462 | Not Available |
| **Vanuatu** | 6,150 | 29,786 | 11,307 | 47,244 | Not Available |
| **Venezuela** | Not Available | 48,539,483 | 20,363,163 | 68,902,646 | 2.5 |
| **Viet Nam** | 1,525,166 | Not Available | Not Available | 1,525,166 | Not Available |
| **Virgin Islands (U.S.)** | Not Available | Not Available | Not Available | Not Available | Not Available |
| **West Bank and Gaza** | Not Available | Not Available | Not Available | Not Available | Not Available |
| **Yemen** | 190,365 | 5,036,273 | 1,091,674 | 6,318,311 | Not Available |
| **Zambia** | 100,662 | 4,725,634 | 334,043 | 5,160,339 | Not Available |
| **Zimbabwe** | 0 | 0 | 0 | 0 | Not Available |

**WEB APPENDIX TABLE 6.** Economic losses due to child and maternal mortality attributable to not recommended breastfeeding practices by country.

|  | **Economic losses due to child mortality attributable to not breastfeeding** (US$ m). | **Economic losses due to maternal mortality attributable to not breastfeeding** (US$ m). | **Combined economic losses due to maternal and child mortality attributable to not breastfeeding** (US$ m). | **Combined economic losses due to maternal and child mortality attributable to not breastfeeding as percent (%) of GNI** |
| --- | --- | --- | --- | --- |
| **Afghanistan** | 295.01 (81.72, 1,273.19) | 0.09 (0.08, 0.11) | 295.10 (81.80, 1,273.30) | 1.48 (0.41, 6.40) |
| **Albania** | 7.52 (1.96, 36.09) | 0.46 (0.38, 0.55) | 7.98 (2.34, 36.64) | 0.06 (0.02, 0.30) |
| **Algeria** | 512.47 (133.18, 2,458.33) | 1.47 (1.23, 1.79) | 513.94 (134.41, 2,460.12) | 0.27 (0.07, 1.27) |
| **American Samoa** | Not Available | Not Available | Not Available | Not Available |
| **Andorra** | Not Available | Not Available | Not Available | Not Available |
| **Angola** | 3,516.44 (1,083.57, 12,872.30) | 0.00 (0.00, 0.00) | 3,516.44 (1,083.57, 12,872.30) | 3.36 (1.04, 12.32) |
| **Antigua and Barbuda** | Not Available | Not Available | Not Available | Not Available |
| **Argentina** | 232.85 (60.51, 1,117.00) | 52.20 (43.58, 63.28) | 285.05 (104.10, 1,180.28) | 0.05 (0.02, 0.22) |
| **Armenia** | 6.80 (1.77, 32.63) | 1.67 (1.39, 2.02) | 8.47 (3.16, 34.65) | 0.07 (0.03, 0.30) |
| **Aruba** | Not Available | Not Available | Not Available | Not Available |
| **Australia** | Not Available | Not Available | Not Available | Not Available |
| **Austria** | Not Available | 94.61 (79.00, 114.70) | Not Available | Not Available |
| **Azerbaijan** | 232.79 (60.50, 1,116.70) | 4.19 (3.50, 5.08) | 236.98 (63.99, 1,121.77) | 0.37 (0.10, 1.77) |
| **Bahamas** | Not Available | Not Available | Not Available | Not Available |
| **Bahrain** | Not Available | Not Available | Not Available | Not Available |
| **Bangladesh** | 314.97 (81.85, 1,510.92) | 0.66 (0.55, 0.79) | 315.63 (82.40, 1,511.72) | 0.16 (0.04, 0.79) |
| **Barbados** | Not Available | Not Available | Not Available | Not Available |
| **Belarus** | 6.18 (1.61, 29.64) | 11.14 (9.30, 13.50) | 17.31 (10.90, 43.14) | 0.03 (0.02, 0.07) |
| **Belgium** | Not Available | Not Available | Not Available | Not Available |
| **Belize** | 1.59 (0.41, 7.65) | 0.05 (0.04, 0.05) | 1.64 (0.45, 7.70) | 0.10 (0.03, 0.48) |
| **Benin** | 152.60 (42.82, 644.99) | 0.10 (0.09, 0.11) | 152.70 (42.91, 645.10) | 1.67 (0.47, 7.04) |
| **Bermuda** | Not Available | Not Available | Not Available | Not Available |
| **Bhutan** | 3.65 (0.95, 17.51) | 0.02 (0.02, 0.03) | 3.67 (0.97, 17.54) | 0.20 (0.05, 0.95) |
| **Bolivia** | 98.45 (25.59, 472.27) | 1.14 (0.95, 1.38) | 99.59 (26.54, 473.65) | 0.31 (0.08, 1.47) |
| **Bosnia and Herzegovina** | 1.22 (0.32, 5.86) | 1.27 (1.06, 1.54) | 2.49 (1.38, 7.40) | 0.01 (0.01, 0.04) |
| **Botswana** | 73.09 (19.24, 343.23) | 1.27 (1.06, 1.53) | 74.36 (20.29, 344.77) | 0.51 (0.14, 2.36) |
| **Brazil** | 1,498.66 (389.47, 7,189.09) | 132.12 (110.31, 160.17) | 1,630.77 (499.78, 7,349.26) | 0.08 (0.02, 0.36) |
| **British Virgin Islands** | Not Available | Not Available | Not Available | Not Available |
| **Brunei** | Not Available | Not Available | Not Available | Not Available |
| **Bulgaria** | Not Available | Not Available | Not Available | Not Available |
| **Burkina Faso** | 82.89 (23.57, 343.14) | 0.04 (0.04, 0.04) | 82.93 (23.61, 343.18) | 0.72 (0.20, 2.96) |
| **Burundi** | 18.19 (5.31, 72.24) | 0.00 (0.00, 0.00) | 18.19 (5.31, 72.24) | 0.63 (0.18, 2.51) |
| **Cabo Verde** | 3.64 (0.95, 17.45) | 0.06 (0.05, 0.07) | 3.69 (0.99, 17.52) | 0.22 (0.06, 1.03) |
| **Cambodia** | 52.23 (13.57, 250.56) | 0.88 (0.74, 1.07) | 53.11 (14.31, 251.63) | 0.32 (0.09, 1.51) |
| **Cameroon** | 450.21 (133.21, 1,751.84) | 0.00 (0.00, 0.00) | 450.21 (133.21, 1,751.84) | 1.47 (0.43, 5.70) |
| **Canada** | Not Available | Not Available | Not Available | Not Available |
| **Cayman Islands** | Not Available | Not Available | Not Available | Not Available |
| **Central African Republic** | 24.41 (7.73, 85.82) | 0.00 (0.00, 0.00) | 24.41 (7.73, 85.82) | 1.49 (0.47, 5.24) |
| **Chad** | 320.10 (100.00, 1,148.36) | 0.00 (0.00, 0.00) | 320.10 (100.00, 1,148.36) | 2.60 (0.81, 9.33) |
| **Channel Islands** | Not Available | Not Available | Not Available | Not Available |
| **Chile** | Not Available | Not Available | Not Available | Not Available |
| **China** | 5,697.19 (1,480.58, 27,329.57) | 568.46 (474.65, 689.18) | 6,265.65 (1,955.24, 28,018.75) | 0.06 (0.02, 0.26) |
| **Colombia** | 226.06 (58.75, 1,084.41) | 17.48 (14.59, 21.19) | 243.54 (73.34, 1,105.60) | 0.07 (0.02, 0.32) |
| **Comoros** | 6.81 (1.81, 31.29) | 0.01 (0.01, 0.01) | 6.81 (1.82, 31.30) | 1.11 (0.30, 5.11) |
| **Congo** | 72.82 (20.70, 301.42) | 0.30 (0.27, 0.32) | 73.11 (20.98, 301.74) | 0.62 (0.18, 2.57) |
| **Costa Rica** | 9.60 (2.59, 43.19) | 1.97 (1.70, 2.31) | 11.57 (4.29, 45.50) | 0.02 (0.01, 0.09) |
| **Côte d'Ivoire** | 554.79 (144.18, 2,661.35) | 0.56 (0.47, 0.68) | 555.35 (144.65, 2,662.03) | 1.73 (0.45, 8.28) |
| **Croatia** | Not Available | 0.00 (0.00, 0.00) | Not Available | Not Available |
| **Cuba** | 11.56 (3.00, 55.43) | 5.64 (4.71, 6.84) | 17.20 (7.72, 62.27) | 0.03 (0.01, 0.09) |
| **Curaçao** | Not Available | Not Available | Not Available | Not Available |
| **Cyprus** | Not Available | Not Available | Not Available | Not Available |
| **Czech Republic** | Not Available | Not Available | Not Available | Not Available |
| **Democratic People's Republic of Korea** | Not Available | Not Available | Not Available | Not Available |
| **Denmark** | Not Available | Not Available | Not Available | Not Available |
| **Djibouti** | Not Available | Not Available | Not Available | Not Available |
| **Dominica** | Not Available | Not Available | Not Available | Not Available |
| **Dominican Republic** | 172.65 (44.87, 828.22) | 2.98 (2.49, 3.62) | 175.63 (47.36, 831.83) | 0.27 (0.07, 1.27) |
| **Democratic Republic of the Congo** | 485.13 (126.08, 2,327.19) | 0.48 (0.40, 0.59) | 485.62 (126.48, 2,327.77) | 1.52 (0.40, 7.31) |
| **Ecuador** | 174.00 (45.22, 834.69) | 3.84 (3.21, 4.65) | 177.84 (48.43, 839.35) | 0.18 (0.05, 0.86) |
| **Egypt** | 861.46 (223.88, 4,132.44) | 4.20 (3.51, 5.10) | 865.66 (227.39, 4,137.53) | 0.28 (0.07, 1.35) |
| **El Salvador** | 19.21 (5.53, 77.89) | 0.12 (0.11, 0.13) | 19.33 (5.65, 78.02) | 0.08 (0.02, 0.32) |
| **Equatorial Guinea** | 200.88 (53.54, 923.47) | 0.73 (0.62, 0.87) | 201.61 (54.16, 924.35) | 1.86 (0.50, 8.53) |
| **Eritrea** | 11.25 (2.92, 53.95) | 0.05 (0.04, 0.06) | 11.29 (2.96, 54.01) | 0.49 (0.13, 2.33) |
| **Estonia** | Not Available | Not Available | Not Available | Not Available |
| **Ethiopia** | 360.50 (93.69, 1,729.31) | 0.93 (0.78, 1.13) | 361.43 (94.46, 1,730.44) | 0.61 (0.16, 2.94) |
| **Faroe Islands** | Not Available | Not Available | Not Available | Not Available |
| **Fiji** | Not Available | Not Available | Not Available | Not Available |
| **Finland** | Not Available | Not Available | Not Available | Not Available |
| **France** | Not Available | Not Available | Not Available | Not Available |
| **French Polynesia** | Not Available | Not Available | Not Available | Not Available |
| **Gabon** | 115.82 (32.08, 499.86) | 0.47 (0.42, 0.53) | 116.29 (32.50, 500.39) | 0.73 (0.20, 3.15) |
| **Gambia** | 9.49 (2.59, 41.82) | 0.01 (0.01, 0.01) | 9.50 (2.60, 41.84) | 1.08 (0.30, 4.77) |
| **Georgia** | 5.66 (1.47, 27.16) | 2.48 (2.07, 3.01) | 8.15 (3.55, 30.17) | 0.05 (0.02, 0.20) |
| **Germany** | Not Available | Not Available | Not Available | Not Available |
| **Ghana** | 227.83 (62.30, 1,004.12) | 0.87 (0.76, 1.00) | 228.71 (63.06, 1,005.12) | 0.56 (0.16, 2.48) |
| **Gibraltar** | Not Available | Not Available | Not Available | Not Available |
| **Greece** | Not Available | Not Available | Not Available | Not Available |
| **Greenland** | Not Available | Not Available | Not Available | Not Available |
| **Grenada** | Not Available | Not Available | Not Available | Not Available |
| **Guam** | Not Available | Not Available | Not Available | Not Available |
| **Guatemala** | 164.54 (46.78, 681.13) | 0.20 (0.19, 0.22) | 164.74 (46.97, 681.35) | 0.28 (0.08, 1.16) |
| **Guinea** | 65.34 (19.33, 254.26) | 0.00 (0.00, 0.00) | 65.34 (19.33, 254.26) | 1.09 (0.32, 4.25) |
| **Guinea-Bissau** | 17.84 (4.64, 85.56) | 0.03 (0.03, 0.04) | 17.87 (4.66, 85.60) | 1.64 (0.43, 7.88) |
| **Guyana** | 6.89 (1.86, 31.01) | 0.11 (0.09, 0.13) | 7.00 (1.95, 31.14) | 0.22 (0.06, 0.99) |
| **Haiti** | 90.57 (25.09, 390.87) | 0.33 (0.29, 0.37) | 90.90 (25.38, 391.24) | 1.04 (0.29, 4.50) |
| **Honduras** | 31.39 (8.16, 150.56) | 0.39 (0.32, 0.47) | 31.78 (8.48, 151.04) | 0.17 (0.05, 0.82) |
| **Hong Kong** | Not Available | Not Available | Not Available | Not Available |
| **Hungary** | Not Available | Not Available | Not Available | Not Available |
| **Iceland** | Not Available | Not Available | Not Available | Not Available |
| **India** | 7,087.44 (1,841.88, 33,998.67) | 13.63 (11.38, 16.53) | 7,101.08 (1,853.26, 34,015.19) | 0.34 (0.09, 1.63) |
| **Indonesia** | 2,300.32 (597.81, 11,034.70) | 24.30 (20.29, 29.46) | 2,324.62 (618.10, 11,064.16) | 0.26 (0.07, 1.25) |
| **Iran** | 494.99 (128.64, 2,374.48) | 2.51 (2.09, 3.04) | 497.50 (130.73, 2,377.52) | 0.10 (0.03, 0.46) |
| **Iraq** | 1,156.47 (300.54, 5,547.64) | 2.90 (2.42, 3.52) | 1,159.38 (302.97, 5,551.16) | 0.55 (0.14, 2.62) |
| **Ireland** | Not Available | Not Available | Not Available | Not Available |
| **Isle of Man** | Not Available | Not Available | Not Available | Not Available |
| **Israel** | Not Available | Not Available | Not Available | Not Available |
| **Italy** | Not Available | Not Available | Not Available | Not Available |
| **Jamaica** | 6.66 (1.73, 31.96) | 1.44 (1.20, 1.74) | 8.10 (2.93, 33.70) | 0.06 (0.02, 0.24) |
| **Japan** | Not Available | Not Available | Not Available | Not Available |
| **Jordan** | 53.16 (13.82, 255.01) | 0.30 (0.25, 0.36) | 53.46 (14.06, 255.37) | 0.15 (0.04, 0.72) |
| **Kazakhstan** | 255.75 (69.93, 1,127.17) | 14.82 (12.99, 17.04) | 270.57 (82.92, 1,144.20) | 0.14 (0.04, 0.57) |
| **Kenya** | 381.89 (99.25, 1,831.93) | 1.22 (1.02, 1.48) | 383.11 (100.26, 1,833.41) | 0.62 (0.16, 2.97) |
| **Kiribati** | 1.70 (0.44, 8.14) | Not Available | 1.70 (0.44, 8.14) | 0.45 (0.12, 2.14) |
| **Kosovo** | Not Available | Not Available | Not Available | Not Available |
| **Kuwait** | 60.02 (15.60, 287.90) | 4.02 (3.35, 4.87) | 64.03 (18.95, 292.77) | 0.04 (0.01, 0.18) |
| **Kyrgyzstan** | 18.83 (4.89, 90.35) | 0.27 (0.23, 0.33) | 19.11 (5.12, 90.68) | 0.27 (0.07, 1.30) |
| **Laos** | 123.64 (32.13, 593.12) | 0.53 (0.44, 0.64) | 124.17 (32.57, 593.76) | 1.05 (0.28, 5.03) |
| **Latvia** | Not Available | Not Available | Not Available | Not Available |
| **Lebanon** | 12.56 (3.26, 60.27) | 1.86 (1.55, 2.25) | 14.42 (4.81, 62.51) | 0.03 (0.01, 0.14) |
| **Lesotho** | 26.86 (8.63, 92.56) | 0.00 (0.00, 0.00) | 26.86 (8.63, 92.56) | 0.98 (0.31, 3.38) |
| **Liberia** | 17.55 (4.86, 75.76) | 0.02 (0.02, 0.02) | 17.57 (4.88, 75.78) | 1.03 (0.29, 4.43) |
| **Libya** | Not Available | Not Available | Not Available | Not Available |
| **Liechtenstein** | Not Available | Not Available | Not Available | Not Available |
| **Lithuania** | Not Available | Not Available | Not Available | Not Available |
| **Luxembourg** | Not Available | Not Available | Not Available | Not Available |
| **Macao SAR, China** | Not Available | Not Available | Not Available | Not Available |
| **Madagascar** | 51.16 (13.30, 245.42) | 0.10 (0.08, 0.12) | 51.26 (13.38, 245.53) | 0.51 (0.13, 2.44) |
| **Malawi** | 42.68 (11.09, 204.75) | 0.06 (0.05, 0.07) | 42.74 (11.14, 204.82) | 0.73 (0.19, 3.48) |
| **Malaysia** | Not Available | Not Available | Not Available | Not Available |
| **Maldives** | 0.72 (0.19, 3.43) | 0.02 (0.02, 0.03) | 0.74 (0.20, 3.46) | 0.03 (0.01, 0.12) |
| **Mali** | 276.50 (71.86, 1,326.36) | 0.12 (0.10, 0.14) | 276.61 (71.95, 1,326.51) | 2.07 (0.54, 9.93) |
| **Malta** | Not Available | Not Available | Not Available | Not Available |
| **Marshall Islands** | 1.13 (0.29, 5.44) | 0.01 (0.01, 0.01) | 1.15 (0.30, 5.46) | 0.45 (0.12, 2.16) |
| **Mauritania** | 67.04 (18.33, 295.47) | 0.04 (0.04, 0.05) | 67.08 (18.37, 295.52) | 1.23 (0.34, 5.43) |
| **Mauritius** | 6.37 (1.70, 29.27) | 1.14 (0.97, 1.36) | 7.51 (2.67, 30.64) | 0.06 (0.02, 0.25) |
| **Mexico** | 1,019.52 (264.95, 4,890.66) | 49.19 (41.07, 59.63) | 1,068.71 (306.02, 4,950.29) | 0.09 (0.02, 0.40) |
| **Micronesia** | Not Available | Not Available | Not Available | Not Available |
| **Moldova** | Not Available | Not Available | Not Available | Not Available |
| **Monaco** | Not Available | Not Available | Not Available | Not Available |
| **Mongolia** | 22.78 (6.23, 100.41) | 0.15 (0.13, 0.18) | 22.93 (6.36, 100.58) | 0.20 (0.06, 0.88) |
| **Montenegro** | 0.26 (0.07, 1.25) | 0.44 (0.36, 0.53) | 0.70 (0.43, 1.78) | 0.02 (0.01, 0.04) |
| **Morocco** | 246.60 (64.09, 1,182.96) | 2.77 (2.32, 3.36) | 249.38 (66.40, 1,186.32) | 0.24 (0.06, 1.12) |
| **Mozambique** | 207.05 (53.81, 993.20) | 0.52 (0.43, 0.63) | 207.57 (54.24, 993.84) | 1.26 (0.33, 6.04) |
| **Myanmar** | 172.79 (51.12, 672.35) | 0.00 (0.00, 0.00) | 172.79 (51.12, 672.35) | 0.28 (0.08, 1.08) |
| **Namibia** | 109.98 (28.58, 527.56) | 0.18 (0.15, 0.22) | 110.16 (28.73, 527.78) | 0.86 (0.23, 4.13) |
| **Nauru** | 0.24 (0.06, 1.11) | Not Available | 0.24 (0.06, 1.11) | 0.15 (0.04, 0.71) |
| **Nepal** | 24.93 (6.82, 109.87) | 0.08 (0.07, 0.10) | 25.02 (6.89, 109.97) | 0.12 (0.03, 0.53) |
| **Netherlands** | Not Available | Not Available | Not Available | Not Available |
| **New Caledonia** | Not Available | Not Available | Not Available | Not Available |
| **New Zealand** | Not Available | Not Available | Not Available | Not Available |
| **Nicaragua** | 26.94 (7.00, 129.25) | 0.22 (0.18, 0.27) | 27.16 (7.19, 129.52) | 0.23 (0.06, 1.10) |
| **Niger** | 205.28 (53.35, 984.75) | 0.05 (0.04, 0.06) | 205.34 (53.39, 984.81) | 2.62 (0.68, 12.58) |
| **Nigeria** | 11,932.62 (3,262.83, 52,589.82) | 4.54 (3.98, 5.22) | 11,937.16 (3,266.81, 52,595.04) | 2.32 (0.64, 10.23) |
| **Northern Mariana Islands** | Not Available | Not Available | Not Available | Not Available |
| **Norway** | Not Available | 67.26 (58.96, 77.33) | Not Available | Not Available |
| **Oman** | 24.18 (6.28, 116.00) | 0.36 (0.30, 0.44) | 24.54 (6.59, 116.44) | 0.03 (0.01, 0.15) |
| **Pakistan** | 2,838.59 (737.69, 13,616.81) | 4.51 (3.77, 5.47) | 2,843.11 (741.46, 13,622.29) | 1.05 (0.27, 5.01) |
| **Palau** | Not Available | Not Available | Not Available | Not Available |
| **Panama** | 60.55 (16.56, 266.86) | 1.32 (1.16, 1.52) | 61.87 (17.71, 268.37) | 0.13 (0.04, 0.57) |
| **Papua New Guinea** | 95.50 (24.82, 458.11) | 0.37 (0.31, 0.45) | 95.87 (25.13, 458.56) | 0.57 (0.15, 2.74) |
| **Paraguay** | 48.88 (13.20, 220.03) | 1.43 (1.23, 1.67) | 50.31 (14.43, 221.70) | 0.18 (0.05, 0.80) |
| **Peru** | 155.78 (40.48, 747.29) | 4.82 (4.03, 5.85) | 160.61 (44.51, 753.14) | 0.08 (0.02, 0.39) |
| **Philippines** | 1,409.71 (366.36, 6,762.43) | 15.03 (12.55, 18.22) | 1,424.74 (378.91, 6,780.65) | 0.40 (0.11, 1.90) |
| **Poland** | Not Available | Not Available | Not Available | Not Available |
| **Portugal** | Not Available | Not Available | Not Available | Not Available |
| **Puerto Rico** | Not Available | Not Available | Not Available | Not Available |
| **Qatar** | 18.52 (4.81, 88.86) | 3.34 (2.79, 4.05) | 21.87 (7.60, 92.91) | 0.01 (0.00, 0.05) |
| **Republic of Korea** | Not Available | Not Available | Not Available | Not Available |
| **Romania** | Not Available | Not Available | Not Available | Not Available |
| **Russia** | Not Available | Not Available | Not Available | Not Available |
| **Rwanda** | 15.73 (4.09, 75.47) | 0.08 (0.07, 0.10) | 15.81 (4.16, 75.57) | 0.20 (0.05, 0.94) |
| **Saint Kitts and Nevis** | Not Available | Not Available | Not Available | Not Available |
| **Saint Lucia** | Not Available | Not Available | Not Available | Not Available |
| **Saint Martin** | Not Available | Not Available | Not Available | Not Available |
| **Saint Vincent and the Grenadines** | Not Available | Not Available | Not Available | Not Available |
| **Samoa** | 0.64 (0.17, 2.95) | 0.00 (0.00, 0.00) | 0.65 (0.17, 2.96) | 0.09 (0.02, 0.39) |
| **San Marino** | Not Available | Not Available | Not Available | Not Available |
| **Sao Tome and Principe** | 1.65 (0.43, 7.91) | 0.01 (0.01, 0.01) | 1.66 (0.44, 7.92) | 0.49 (0.13, 2.36) |
| **Saudi Arabia** | Not Available | 3.52 (2.94, 4.27) | Not Available | Not Available |
| **Senegal** | 114.42 (29.74, 548.87) | 0.22 (0.18, 0.27) | 114.64 (29.92, 549.14) | 0.77 (0.20, 3.69) |
| **Serbia** | 4.39 (1.14, 21.06) | 7.82 (6.53, 9.48) | 12.21 (7.67, 30.55) | 0.03 (0.02, 0.08) |
| **Seychelles** | Not Available | Not Available | Not Available | Not Available |
| **Sierra Leone** | 79.62 (20.69, 381.96) | 0.09 (0.08, 0.11) | 79.72 (20.77, 382.07) | 1.99 (0.52, 9.53) |
| **Singapore** | Not Available | 0.00 (0.00, 0.00) | Not Available | Not Available |
| **Sint Maarten (Dutch part)** | Not Available | Not Available | Not Available | Not Available |
| **Slovakia** | Not Available | Not Available | Not Available | Not Available |
| **Slovenia** | Not Available | Not Available | Not Available | Not Available |
| **Solomon Islands** | 3.08 (0.80, 14.80) | 0.03 (0.02, 0.03) | 3.11 (0.82, 14.83) | 0.28 (0.07, 1.32) |
| **Somalia** | Not Available | Not Available | Not Available | Not Available |
| **South Africa** | 1,319.03 (390.27, 5,132.57) | 0.00 (0.00, 0.00) | 1,319.03 (390.27, 5,132.57) | 0.39 (0.12, 1.54) |
| **South Sudan** | 190.01 (49.38, 911.48) | Not Available | 190.01 (49.38, 911.48) | 1.96 (0.51, 9.39) |
| **Spain** | Not Available | 0.00 (0.00, 0.00) | Not Available | Not Available |
| **Sri Lanka** | 12.02 (3.12, 57.68) | 0.41 (0.34, 0.50) | 12.44 (3.47, 58.18) | 0.02 (0.00, 0.07) |
| **Sudan** | 714.83 (203.24, 2,959.08) | 0.21 (0.20, 0.23) | 715.05 (203.44, 2,959.31) | 0.93 (0.26, 3.83) |
| **Suriname** | 4.19 (1.12, 19.26) | 0.24 (0.21, 0.29) | 4.43 (1.32, 19.55) | 0.09 (0.03, 0.38) |
| **Swaziland** | 45.96 (11.94, 220.46) | 0.17 (0.14, 0.21) | 46.13 (12.09, 220.67) | 1.09 (0.29, 5.22) |
| **Sweden** | Not Available | 0.00 (0.00, 0.00) | Not Available | Not Available |
| **Switzerland** | Not Available | Not Available | Not Available | Not Available |
| **Syria** | 56.41 (14.66, 270.60) | 0.16 (0.13, 0.19) | 56.57 (14.79, 270.79) | 0.16 (0.04, 0.75) |
| **Taiwan** | Not Available | Not Available | Not Available | Not Available |
| **Tajikistan** | 77.39 (20.11, 371.26) | 0.18 (0.15, 0.21) | 77.57 (20.26, 371.47) | 0.72 (0.19, 3.43) |
| **Tanzania** | 392.40 (101.98, 1,882.37) | 1.46 (1.22, 1.77) | 393.86 (103.20, 1,884.14) | 0.83 (0.22, 3.95) |
| **Thailand** | 160.20 (42.16, 752.28) | 39.18 (32.71, 47.50) | 199.38 (74.87, 799.78) | 0.05 (0.02, 0.21) |
| **The former Yugoslav Republic of Macedonia** | 1.22 (0.32, 5.87) | 1.10 (0.92, 1.33) | 2.32 (1.24, 7.20) | 0.02 (0.01, 0.07) |
| **Timor-Leste** | 31.36 (8.15, 150.43) | 0.03 (0.03, 0.04) | 31.39 (8.18, 150.47) | 1.16 (0.30, 5.56) |
| **Togo** | 32.96 (9.25, 139.29) | 0.03 (0.03, 0.04) | 32.99 (9.28, 139.33) | 0.83 (0.23, 3.51) |
| **Tonga** | 0.56 (0.15, 2.68) | 0.04 (0.03, 0.05) | 0.60 (0.18, 2.73) | 0.13 (0.04, 0.60) |
| **Trinidad and Tobago** | 17.31 (4.50, 83.03) | 2.81 (2.35, 3.41) | 20.12 (6.84, 86.43) | 0.08 (0.03, 0.36) |
| **Tunisia** | 30.77 (8.00, 147.62) | 1.19 (0.99, 1.44) | 31.96 (8.99, 149.07) | 0.07 (0.02, 0.34) |
| **Turkey** | 203.97 (53.01, 978.44) | 16.11 (13.45, 19.53) | 220.07 (66.46, 997.97) | 0.03 (0.01, 0.13) |
| **Turkmenistan** | 231.60 (60.19, 1,111.00) | 1.51 (1.26, 1.83) | 233.11 (61.45, 1,112.83) | 0.59 (0.15, 2.81) |
| **Turks and Caicos Islands** | Not Available | Not Available | Not Available | Not Available |
| **Tuvalu** | 0.12 (0.03, 0.52) | Not Available | 0.12 (0.03, 0.52) | 0.19 (0.05, 0.83) |
| **Uganda** | 217.00 (61.70, 898.26) | 0.26 (0.24, 0.28) | 217.25 (61.93, 898.54) | 0.80 (0.23, 3.30) |
| **Ukraine** | 29.82 (7.75, 143.05) | 19.76 (16.50, 23.95) | 49.58 (24.25, 167.00) | 0.04 (0.02, 0.15) |
| **United Arab Emirates** | 28.88 (7.51, 138.55) | 5.70 (4.76, 6.91) | 34.58 (12.27, 145.46) | 0.01 (0.00, 0.04) |
| **United Kingdom** | Not Available | Not Available | Not Available | Not Available |
| **United States** | Not Available | Not Available | Not Available | Not Available |
| **Uruguay** | Not Available | Not Available | Not Available | Not Available |
| **Uzbekistan** | 267.61 (69.55, 1,283.74) | 1.46 (1.22, 1.77) | 269.07 (70.77, 1,285.50) | 0.40 (0.10, 1.90) |
| **Vanuatu** | 2.82 (0.73, 13.51) | 0.04 (0.03, 0.04) | 2.85 (0.76, 13.55) | 0.35 (0.09, 1.65) |
| **Venezuela** | Not Available | Not Available | Not Available | Not Available |
| **Viet Nam** | 316.32 (82.20, 1,517.39) | 12.02 (10.03, 14.57) | 328.33 (92.24, 1,531.95) | 0.18 (0.05, 0.84) |
| **Virgin Islands (U.S.)** | Not Available | Not Available | Not Available | Not Available |
| **West Bank and Gaza** | Not Available | Not Available | Not Available | Not Available |
| **Yemen** | 202.38 (53.94, 930.37) | 0.28 (0.24, 0.34) | 202.66 (54.18, 930.71) | 0.66 (0.18, 3.04) |
| **Zambia** | 204.50 (56.64, 882.56) | 0.52 (0.47, 0.59) | 205.02 (57.11, 883.15) | 0.85 (0.24, 3.65) |
| **Zimbabwe** | 141.52 (40.77, 573.82) | 0.15 (0.14, 0.16) | 141.67 (40.91, 573.98) | 1.05 (0.30, 4.26) |

**WEB APPENDIX TABLE 7.** Economic losses due to cognitive losses in children attributable to not recommended breastfeeding practices a by country and by approach.

|  | **Economic losses attributable to cognitive losses related to not breastfeeding (EBF 0-5 months vs. non-EBF) (NPV of future potential earnings with increased breastfeeding at full coverage)** (US$ m). | **Economic losses attributable cognitive losses related to not breastfeeding (EBF 0-5 months vs. non-EBF) as percent (%) of GNI** | **Sensitivity analysis: Economic losses attributable to cognitive losses related to not breastfeeding (BF at 6 months vs. no BF) (NPV of future potential earnings with increased breastfeeding at full coverage)** (US$ m). | **Sensitivity analysis: Economic losses attributable to cognitive losses related to not breastfeeding (BF at 6 months vs. no BF) as percent (%) of GNI** |
| --- | --- | --- | --- | --- |
| **Afghanistan** | 192.74 (53.39, 831.81) | 0.97 (0.27, 4.18) | 23.45 (6.50, 101.23) | 0.12 (0.03, 0.51) |
| **Albania** | 75.24 (19.55, 360.91) | 0.61 (0.16, 2.92) | 16.91 (4.39, 81.12) | 0.14 (0.04, 0.66) |
| **Algeria** | 2,172.13 (564.49, 10,419.75) | 1.13 (0.29, 5.40) | Not Available | Not Available |
| **American Samoa** | Not Available | Not Available | Not Available | Not Available |
| **Andorra** | Not Available | Not Available | Not Available | Not Available |
| **Angola** | 1,238.72 (381.71, 4,534.47) | 1.19 (0.37, 4.34) | Not Available | Not Available |
| **Antigua and Barbuda** | Not Available | Not Available | Not Available | Not Available |
| **Argentina** | 3,976.03 (1,033.29, 19,073.15) | 0.73 (0.19, 3.52) | 1,352.92 (351.59, 6,489.97) | 0.25 (0.06, 1.20) |
| **Armenia** | 40.78 (10.60, 195.62) | 0.35 (0.09, 1.67) | 18.00 (4.68, 86.36) | 0.15 (0.04, 0.74) |
| **Aruba** | Not Available | Not Available | Not Available | Not Available |
| **Australia** | Not Available | Not Available | 5,078.61 (1,319.83, 24,362.21) | 0.36 (0.09, 1.71) |
| **Austria** | Not Available | Not Available | 1,527.58 (396.99, 7,327.82) | 0.37 (0.10, 1.80) |
| **Azerbaijan** | 347.25 (90.24, 1,665.76) | 0.55 (0.14, 2.63) | 135.11 (35.11, 648.11) | 0.21 (0.06, 1.02) |
| **Bahamas** | Not Available | Not Available | Not Available | Not Available |
| **Bahrain** | 182.42 (47.41, 875.06) | 0.67 (0.17, 3.20) | Not Available | Not Available |
| **Bangladesh** | 1,060.51 (275.60, 5,087.28) | 0.55 (0.14, 2.66) | 73.55 (19.11, 352.81) | 0.04 (0.01, 0.18) |
| **Barbados** | 25.11 (6.53, 120.47) | 0.61 (0.16, 2.92) | Not Available | Not Available |
| **Belarus** | 461.88 (120.03, 2,215.63) | 0.75 (0.20, 3.61) | 305.07 (79.28, 1,463.41) | 0.50 (0.13, 2.38) |
| **Belgium** | 4,095.45 (1,064.32, 19,645.97) | 0.82 (0.21, 3.93) | Not Available | Not Available |
| **Belize** | 16.04 (4.17, 76.95) | 0.99 (0.26, 4.77) | 8.26 (2.15, 39.63) | 0.51 (0.13, 2.45) |
| **Benin** | 98.96 (27.77, 418.26) | 1.08 (0.30, 4.57) | 14.86 (4.17, 62.81) | 0.16 (0.05, 0.69) |
| **Bermuda** | Not Available | Not Available | Not Available | Not Available |
| **Bhutan** | 8.97 (2.33, 43.05) | 0.49 (0.13, 2.33) | 0.11 (0.03, 0.53) | 0.01 (0.00, 0.03) |
| **Bolivia** | 164.92 (42.86, 791.12) | 0.51 (0.13, 2.46) | 20.33 (5.28, 97.50) | 0.06 (0.02, 0.30) |
| **Bosnia and Herzegovina** | 75.61 (19.65, 362.72) | 0.42 (0.11, 2.04) | Not Available | Not Available |
| **Botswana** | 182.05 (47.91, 854.86) | 1.25 (0.33, 5.85) | 10.28 (2.71, 48.27) | 0.07 (0.02, 0.33) |
| **Brazil** | 12,327.77 (3,203.74, 59,136.65) | 0.60 (0.16, 2.89) | 5,501.32 (1,429.68, 26,389.97) | 0.27 (0.07, 1.29) |
| **British Virgin Islands** | Not Available | Not Available | Not Available | Not Available |
| **Brunei** | Not Available | Not Available | Not Available | Not Available |
| **Bulgaria** | Not Available | Not Available | Not Available | Not Available |
| **Burkina Faso** | 115.05 (32.71, 476.26) | 0.99 (0.28, 4.11) | 4.38 (1.25, 18.13) | 0.04 (0.01, 0.16) |
| **Burundi** | 10.17 (2.97, 40.39) | 0.35 (0.10, 1.40) | 1.02 (0.30, 4.06) | 0.04 (0.01, 0.14) |
| **Cabo Verde** | 9.10 (2.37, 43.66) | 0.53 (0.14, 2.56) | Not Available | Not Available |
| **Cambodia** | 83.61 (21.73, 401.10) | 0.50 (0.13, 2.40) | 14.18 (3.68, 68.00) | 0.08 (0.02, 0.41) |
| **Cameroon** | 378.54 (112.00, 1,472.95) | 1.23 (0.36, 4.80) | 17.40 (5.15, 67.70) | 0.06 (0.02, 0.22) |
| **Canada** | Not Available | Not Available | 8,455.34 (2,197.37, 40,560.51) | 0.50 (0.13, 2.38) |
| **Cayman Islands** | Not Available | Not Available | Not Available | Not Available |
| **Central African Republic** | 14.66 (4.64, 51.54) | 0.89 (0.28, 3.15) | 0.76 (0.24, 2.67) | 0.05 (0.01, 0.16) |
| **Chad** | 226.51 (70.76, 812.61) | 1.84 (0.57, 6.60) | 9.31 (2.91, 33.42) | 0.08 (0.02, 0.27) |
| **Channel Islands** | Not Available | Not Available | Not Available | Not Available |
| **Chile** | Not Available | Not Available | 1,065.43 (276.88, 5,110.89) | 0.42 (0.11, 2.02) |
| **China** | 59,602.16 (15,489.38, 285,913.20) | 0.55 (0.14, 2.63) | 17,910.75 (4,654.64, 85,918.36) | 0.16 (0.04, 0.79) |
| **Colombia** | 1,938.27 (503.72, 9,297.92) | 0.56 (0.15, 2.70) | 684.49 (177.89, 3,283.53) | 0.20 (0.05, 0.95) |
| **Comoros** | 10.54 (2.81, 48.47) | 1.72 (0.46, 7.91) | 1.37 (0.36, 6.29) | 0.22 (0.06, 1.03) |
| **Congo** | 149.13 (42.40, 617.33) | 1.27 (0.36, 5.27) | 14.89 (4.23, 61.64) | 0.13 (0.04, 0.53) |
| **Costa Rica** | 174.56 (47.12, 785.69) | 0.35 (0.09, 1.57) | 24.05 (6.49, 108.25) | 0.05 (0.01, 0.22) |
| **Côte d'Ivoire** | 630.58 (163.88, 3,024.92) | 1.96 (0.51, 9.40) | 29.41 (7.64, 141.09) | 0.09 (0.02, 0.44) |
| **Croatia** | 180.74 (56.46, 648.42) | 0.34 (0.11, 1.21) | Not Available | Not Available |
| **Cuba** | 209.64 (54.48, 1,005.67) | 0.31 (0.08, 1.51) | 91.64 (23.82, 439.60) | 0.14 (0.04, 0.66) |
| **Curaçao** | Not Available | Not Available | Not Available | Not Available |
| **Cyprus** | Not Available | Not Available | Not Available | Not Available |
| **Czech Republic** | Not Available | Not Available | 572.26 (148.72, 2,745.16) | 0.30 (0.08, 1.43) |
| **Democratic People's Republic of Korea** | Not Available | Not Available | Not Available | Not Available |
| **Denmark** | Not Available | Not Available | 1,718.19 (463.82, 7,733.65) | 0.52 (0.14, 2.33) |
| **Djibouti** | Not Available | Not Available | Not Available | Not Available |
| **Dominica** | Not Available | Not Available | Not Available | Not Available |
| **Dominican Republic** | 808.21 (210.04, 3,877.02) | 1.23 (0.32, 5.90) | 401.99 (104.47, 1,928.34) | 0.61 (0.16, 2.94) |
| **Democratic Republic of the Congo** | 409.27 (106.36, 1,963.29) | 1.29 (0.33, 6.16) | 21.09 (5.48, 101.16) | 0.07 (0.02, 0.32) |
| **Ecuador** | 747.83 (194.34, 3,587.34) | 0.77 (0.20, 3.68) | 292.90 (76.12, 1,405.04) | 0.30 (0.08, 1.44) |
| **Egypt** | 3,557.17 (924.43, 17,063.83) | 1.16 (0.30, 5.58) | 530.92 (137.98, 2,546.84) | 0.17 (0.05, 0.83) |
| **El Salvador** | 115.47 (33.27, 468.21) | 0.48 (0.14, 1.94) | Not Available | Not Available |
| **Equatorial Guinea** | 197.78 (52.71, 909.24) | 1.83 (0.49, 8.39) | Not Available | Not Available |
| **Eritrea** | 15.60 (4.05, 74.82) | 0.67 (0.18, 3.23) | 1.00 (0.26, 4.78) | 0.04 (0.01, 0.21) |
| **Estonia** | Not Available | Not Available | Not Available | Not Available |
| **Ethiopia** | 490.41 (127.45, 2,352.52) | 0.83 (0.22, 4.00) | 27.69 (7.20, 132.85) | 0.05 (0.01, 0.23) |
| **Faroe Islands** | Not Available | Not Available | Not Available | Not Available |
| **Fiji** | 31.47 (8.18, 150.98) | 0.73 (0.19, 3.51) | Not Available | Not Available |
| **Finland** | Not Available | Not Available | 705.21 (192.83, 3,108.02) | 0.28 (0.08, 1.22) |
| **France** | Not Available | Not Available | 17,538.60 (4,557.92, 84,133.15) | 0.65 (0.17, 3.11) |
| **French Polynesia** | Not Available | Not Available | Not Available | Not Available |
| **Gabon** | 248.54 (68.84, 1,072.64) | 1.57 (0.43, 6.76) | 49.97 (13.84, 215.67) | 0.31 (0.09, 1.36) |
| **Gambia** | 11.44 (3.13, 50.40) | 1.30 (0.36, 5.74) | 0.34 (0.09, 1.52) | 0.04 (0.01, 0.17) |
| **Georgia** | 37.76 (9.81, 181.16) | 0.25 (0.06, 1.18) | Not Available | Not Available |
| **Germany** | Not Available | Not Available | 10,086.38 (2,758.00, 44,453.02) | 0.27 (0.07, 1.19) |
| **Ghana** | 359.62 (98.34, 1,584.95) | 0.89 (0.24, 3.91) | 4.52 (1.24, 19.94) | 0.01 (0.00, 0.05) |
| **Gibraltar** | Not Available | Not Available | Not Available | Not Available |
| **Greece** | Not Available | Not Available | 1,033.04 (268.46, 4,955.50) | 0.47 (0.12, 2.25) |
| **Greenland** | Not Available | Not Available | Not Available | Not Available |
| **Grenada** | 6.67 (1.73, 32.01) | 0.72 (0.19, 3.46) | Not Available | Not Available |
| **Guam** | Not Available | Not Available | Not Available | Not Available |
| **Guatemala** | 400.99 (114.01, 1,659.91) | 0.68 (0.19, 2.83) | 68.55 (19.49, 283.75) | 0.12 (0.03, 0.48) |
| **Guinea** | 23.69 (7.01, 92.19) | 0.40 (0.12, 1.54) | 0.69 (0.20, 2.67) | 0.01 (0.00, 0.04) |
| **Guinea-Bissau** | 11.14 (2.89, 53.42) | 1.02 (0.27, 4.92) | Not Available | Not Available |
| **Guyana** | 31.45 (8.49, 141.55) | 1.00 (0.27, 4.51) | 6.60 (1.78, 29.71) | 0.21 (0.06, 0.95) |
| **Haiti** | 69.48 (19.24, 299.84) | 0.80 (0.22, 3.45) | 5.42 (1.50, 23.37) | 0.06 (0.02, 0.27) |
| **Honduras** | 161.25 (41.90, 773.50) | 0.88 (0.23, 4.20) | 46.87 (12.18, 224.86) | 0.25 (0.07, 1.22) |
| **Hong Kong** | Not Available | Not Available | Not Available | Not Available |
| **Hungary** | Not Available | Not Available | Not Available | Not Available |
| **Iceland** | Not Available | Not Available | Not Available | Not Available |
| **India** | 7,250.68 (1,884.30, 34,781.69) | 0.35 (0.09, 1.66) | 385.85 (100.27, 1,850.91) | 0.02 (0.00, 0.09) |
| **Indonesia** | 6,943.75 (1,804.54, 33,309.38) | 0.78 (0.20, 3.76) | 1,365.01 (354.74, 6,548.00) | 0.15 (0.04, 0.74) |
| **Iran** | 2,625.77 (682.38, 12,595.89) | 0.51 (0.13, 2.46) | Not Available | Not Available |
| **Iraq** | 3,587.32 (932.27, 17,208.49) | 1.69 (0.44, 8.12) | Not Available | Not Available |
| **Ireland** | Not Available | Not Available | Not Available | Not Available |
| **Isle of Man** | Not Available | Not Available | Not Available | Not Available |
| **Israel** | Not Available | Not Available | Not Available | Not Available |
| **Italy** | Not Available | Not Available | 5,470.84 (1,421.76, 26,243.75) | 0.27 (0.07, 1.32) |
| **Jamaica** | 97.44 (25.32, 467.44) | 0.71 (0.18, 3.40) | 21.36 (5.55, 102.44) | 0.16 (0.04, 0.74) |
| **Japan** | Not Available | Not Available | 10,519.95 (2,733.92, 50,464.50) | 0.21 (0.06, 1.02) |
| **Jordan** | 472.08 (122.68, 2,264.58) | 1.33 (0.35, 6.37) | 151.46 (39.36, 726.54) | 0.43 (0.11, 2.04) |
| **Kazakhstan** | 1,092.07 (298.61, 4,812.99) | 0.55 (0.15, 2.41) | 256.34 (70.09, 1,129.74) | 0.13 (0.04, 0.57) |
| **Kenya** | 490.04 (127.35, 2,350.74) | 0.79 (0.21, 3.80) | 8.89 (2.31, 42.63) | 0.01 (0.00, 0.07) |
| **Kiribati** | 1.85 (0.48, 8.88) | 0.49 (0.13, 2.33) | Not Available | Not Available |
| **Kosovo** | Not Available | Not Available | Not Available | Not Available |
| **Kuwait** | 1,643.08 (427.00, 7,881.91) | 1.00 (0.26, 4.80) | Not Available | Not Available |
| **Kyrgyzstan** | 47.55 (12.36, 228.11) | 0.68 (0.18, 3.27) | 4.52 (1.17, 21.69) | 0.06 (0.02, 0.31) |
| **Laos** | 110.78 (28.79, 531.41) | 0.94 (0.24, 4.50) | Not Available | Not Available |
| **Latvia** | Not Available | Not Available | Not Available | Not Available |
| **Lebanon** | 369.15 (95.94, 1,770.83) | 0.82 (0.21, 3.93) | Not Available | Not Available |
| **Lesotho** | 10.76 (3.46, 37.08) | 0.39 (0.13, 1.35) | 4.75 (1.52, 16.36) | 0.17 (0.06, 0.60) |
| **Liberia** | 14.23 (3.94, 61.43) | 0.83 (0.23, 3.59) | 0.76 (0.21, 3.29) | 0.04 (0.01, 0.19) |
| **Libya** | Not Available | Not Available | Not Available | Not Available |
| **Liechtenstein** | Not Available | Not Available | Not Available | Not Available |
| **Lithuania** | Not Available | Not Available | Not Available | Not Available |
| **Luxembourg** | Not Available | Not Available | Not Available | Not Available |
| **Macao SAR, China** | Not Available | Not Available | Not Available | Not Available |
| **Madagascar** | 125.14 (32.52, 600.29) | 1.24 (0.32, 5.95) | 2.80 (0.73, 13.43) | 0.03 (0.01, 0.13) |
| **Malawi** | 51.56 (13.40, 247.32) | 0.88 (0.23, 4.21) | 9.83 (2.56, 47.17) | 0.17 (0.04, 0.80) |
| **Malaysia** | 1,656.64 (447.21, 7,456.60) | 0.52 (0.14, 2.33) | Not Available | Not Available |
| **Maldives** | 18.14 (4.71, 87.03) | 0.64 (0.17, 3.06) | 2.47 (0.64, 11.84) | 0.09 (0.02, 0.42) |
| **Mali** | 225.32 (58.56, 1,080.89) | 1.69 (0.44, 8.09) | 15.38 (4.00, 73.77) | 0.12 (0.03, 0.55) |
| **Malta** | Not Available | Not Available | Not Available | Not Available |
| **Marshall Islands** | 2.19 (0.57, 10.52) | 0.87 (0.23, 4.17) | 3.19 (0.83, 15.32) | 1.26 (0.33, 6.06) |
| **Mauritania** | 59.01 (16.14, 260.07) | 1.08 (0.30, 4.78) | 3.32 (0.91, 14.65) | 0.06 (0.02, 0.27) |
| **Mauritius** | 51.88 (13.83, 238.51) | 0.42 (0.11, 1.93) | Not Available | Not Available |
| **Mexico** | 7,129.79 (1,852.89, 34,201.78) | 0.58 (0.15, 2.77) | Not Available | Not Available |
| **Micronesia** | 9.39 (2.44, 45.07) | 2.53 (0.66, 12.12) | Not Available | Not Available |
| **Moldova** | Not Available | Not Available | 13.57 (3.53, 65.08) | 0.17 (0.04, 0.82) |
| **Monaco** | Not Available | Not Available | Not Available | Not Available |
| **Mongolia** | 43.62 (11.93, 192.25) | 0.38 (0.10, 1.68) | Not Available | Not Available |
| **Montenegro** | 26.40 (6.86, 126.64) | 0.59 (0.15, 2.82) | Not Available | Not Available |
| **Morocco** | 992.75 (257.99, 4,762.23) | 0.94 (0.24, 4.49) | 250.25 (65.03, 1,200.45) | 0.24 (0.06, 1.13) |
| **Mozambique** | 227.51 (59.13, 1,091.39) | 1.38 (0.36, 6.64) | 8.87 (2.30, 42.55) | 0.05 (0.01, 0.26) |
| **Myanmar** | 256.09 (75.77, 996.48) | 0.41 (0.12, 1.60) | Not Available | Not Available |
| **Namibia** | 125.01 (32.49, 599.67) | 0.98 (0.25, 4.70) | 52.43 (13.63, 251.51) | 0.41 (0.11, 1.97) |
| **Nauru** | 0.00 (0.00, 0.00) | 0.00 (0.00, 0.00) | Not Available | Not Available |
| **Nepal** | 78.29 (21.41, 345.03) | 0.37 (0.10, 1.65) | 1.15 (0.32, 5.09) | 0.01 (0.00, 0.02) |
| **Netherlands** | Not Available | Not Available | 4,383.88 (1,139.28, 21,029.61) | 0.53 (0.14, 2.54) |
| **New Caledonia** | Not Available | Not Available | Not Available | Not Available |
| **New Zealand** | Not Available | Not Available | 596.00 (154.89, 2,859.05) | 0.32 (0.08, 1.55) |
| **Nicaragua** | 100.07 (26.01, 480.02) | 0.85 (0.22, 4.06) | 33.11 (8.60, 158.84) | 0.28 (0.07, 1.34) |
| **Niger** | 53.85 (13.99, 258.30) | 0.69 (0.18, 3.30) | 0.98 (0.26, 4.71) | 0.01 (0.00, 0.06) |
| **Nigeria** | 9,098.20 (2,487.79, 40,097.86) | 1.77 (0.48, 7.80) | 495.66 (135.53, 2,184.51) | 0.10 (0.03, 0.42) |
| **Northern Mariana Islands** | Not Available | Not Available | Not Available | Not Available |
| **Norway** | Not Available | Not Available | 953.43 (260.70, 4,201.98) | 0.20 (0.05, 0.86) |
| **Oman** | 586.80 (152.50, 2,814.89) | 0.77 (0.20, 3.71) | 742.23 (192.89, 3,560.50) | 0.98 (0.25, 4.69) |
| **Pakistan** | 2,926.07 (760.42, 14,036.42) | 1.08 (0.28, 5.17) | 582.40 (151.35, 2,793.77) | 0.21 (0.06, 1.03) |
| **Palau** | Not Available | Not Available | Not Available | Not Available |
| **Panama** | 406.78 (111.23, 1,792.79) | 0.87 (0.24, 3.84) | Not Available | Not Available |
| **Papua New Guinea** | 127.54 (33.15, 611.82) | 0.76 (0.20, 3.66) | Not Available | Not Available |
| **Paraguay** | 253.58 (68.45, 1,141.37) | 0.91 (0.25, 4.10) | 101.30 (27.35, 455.94) | 0.36 (0.10, 1.64) |
| **Peru** | 479.90 (124.72, 2,302.11) | 0.25 (0.06, 1.20) | 100.23 (26.05, 480.82) | 0.05 (0.01, 0.25) |
| **Philippines** | 2,316.35 (601.97, 11,111.61) | 0.65 (0.17, 3.11) | 779.14 (202.48, 3,737.54) | 0.22 (0.06, 1.05) |
| **Poland** | Not Available | Not Available | Not Available | Not Available |
| **Portugal** | Not Available | Not Available | Not Available | Not Available |
| **Puerto Rico** | Not Available | Not Available | Not Available | Not Available |
| **Qatar** | 391.79 (101.82, 1,879.45) | 0.21 (0.05, 1.00) | Not Available | Not Available |
| **Republic of Korea** | Not Available | Not Available | 3,671.90 (954.25, 17,614.19) | 0.26 (0.07, 1.27) |
| **Romania** | 599.03 (155.68, 2,873.58) | 0.32 (0.08, 1.53) | Not Available | Not Available |
| **Russia** | Not Available | Not Available | Not Available | Not Available |
| **Rwanda** | 18.74 (4.87, 89.91) | 0.23 (0.06, 1.11) | 1.33 (0.35, 6.37) | 0.02 (0.00, 0.08) |
| **Saint Kitts and Nevis** | Not Available | Not Available | Not Available | Not Available |
| **Saint Lucia** | Not Available | Not Available | Not Available | Not Available |
| **Saint Martin** | Not Available | Not Available | Not Available | Not Available |
| **Saint Vincent and the Grenadines** | Not Available | Not Available | Not Available | Not Available |
| **Samoa** | 5.23 (1.39, 24.06) | 0.69 (0.18, 3.17) | Not Available | Not Available |
| **San Marino** | Not Available | Not Available | Not Available | Not Available |
| **Sao Tome and Principe** | 1.83 (0.48, 8.78) | 0.55 (0.14, 2.62) | 0.03 (0.01, 0.13) | 0.01 (0.00, 0.04) |
| **Saudi Arabia** | Not Available | Not Available | Not Available | Not Available |
| **Senegal** | 235.56 (61.22, 1,129.99) | 1.58 (0.41, 7.60) | 1.77 (0.46, 8.47) | 0.01 (0.00, 0.06) |
| **Serbia** | 275.81 (71.68, 1,323.08) | 0.70 (0.18, 3.36) | Not Available | Not Available |
| **Seychelles** | Not Available | Not Available | Not Available | Not Available |
| **Sierra Leone** | 55.46 (14.41, 266.03) | 1.38 (0.36, 6.63) | 4.32 (1.12, 20.73) | 0.11 (0.03, 0.52) |
| **Singapore** | Not Available | Not Available | 629.44 (199.37, 2,213.18) | 0.22 (0.07, 0.77) |
| **Sint Maarten (Dutch part)** | Not Available | Not Available | Not Available | Not Available |
| **Slovakia** | Not Available | Not Available | Not Available | Not Available |
| **Slovenia** | Not Available | Not Available | Not Available | Not Available |
| **Solomon Islands** | 5.31 (1.38, 25.45) | 0.47 (0.12, 2.27) | Not Available | Not Available |
| **Somalia** | Not Available | Not Available | Not Available | Not Available |
| **South Africa** | 925.45 (273.82, 3,601.10) | 0.28 (0.08, 1.08) | 392.37 (116.09, 1,526.78) | 0.12 (0.03, 0.46) |
| **South Sudan** | 114.68 (29.80, 550.12) | 1.18 (0.31, 5.67) | Not Available | Not Available |
| **Spain** | Not Available | Not Available | 2,989.76 (884.61, 11,633.68) | 0.23 (0.07, 0.88) |
| **Sri Lanka** | 189.68 (49.29, 909.89) | 0.24 (0.06, 1.14) | 105.81 (27.50, 507.58) | 0.13 (0.03, 0.64) |
| **Sudan** | 584.16 (166.09, 2,418.16) | 0.76 (0.21, 3.13) | 37.98 (10.80, 157.23) | 0.05 (0.01, 0.20) |
| **Suriname** | 53.81 (14.34, 247.38) | 1.06 (0.28, 4.87) | 19.99 (5.33, 91.88) | 0.39 (0.10, 1.81) |
| **Swaziland** | 27.06 (7.03, 129.79) | 0.64 (0.17, 3.07) | 12.48 (3.24, 59.88) | 0.30 (0.08, 1.42) |
| **Sweden** | Not Available | Not Available | 1,234.91 (402.21, 4,172.17) | 0.22 (0.07, 0.74) |
| **Switzerland** | Not Available | Not Available | 2,188.43 (568.73, 10,497.97) | 0.31 (0.08, 1.50) |
| **Syria** | 287.70 (74.77, 1,380.08) | 0.80 (0.21, 3.81) | Not Available | Not Available |
| **Taiwan** | Not Available | Not Available | Not Available | Not Available |
| **Tajikistan** | 140.39 (36.48, 673.44) | 1.30 (0.34, 6.21) | 16.67 (4.33, 79.95) | 0.15 (0.04, 0.74) |
| **Tanzania** | 481.00 (125.00, 2,307.37) | 1.01 (0.26, 4.84) | 28.29 (7.35, 135.73) | 0.06 (0.02, 0.28) |
| **Thailand** | 2,027.96 (533.70, 9,523.01) | 0.52 (0.14, 2.45) | 508.97 (133.95, 2,390.04) | 0.13 (0.03, 0.62) |
| **The former Yugoslav Republic of Macedonia** | 53.82 (13.99, 258.17) | 0.50 (0.13, 2.42) | 20.90 (5.43, 100.25) | 0.20 (0.05, 0.94) |
| **Timor-Leste** | 26.56 (6.90, 127.40) | 0.98 (0.25, 4.70) | 1.76 (0.46, 8.45) | 0.07 (0.02, 0.31) |
| **Togo** | 31.04 (8.71, 131.19) | 0.78 (0.22, 3.30) | 1.02 (0.29, 4.32) | 0.03 (0.01, 0.11) |
| **Tonga** | 3.29 (0.86, 15.79) | 0.72 (0.19, 3.47) | Not Available | Not Available |
| **Trinidad and Tobago** | 183.29 (47.63, 879.23) | 0.76 (0.20, 3.66) | 80.92 (21.03, 388.19) | 0.34 (0.09, 1.62) |
| **Tunisia** | 486.36 (126.40, 2,333.09) | 1.10 (0.29, 5.28) | 116.94 (30.39, 560.96) | 0.26 (0.07, 1.27) |
| **Turkey** | 5,808.77 (1,509.58, 27,864.84) | 0.74 (0.19, 3.56) | 1,944.57 (505.35, 9,328.15) | 0.25 (0.06, 1.19) |
| **Turkmenistan** | 214.95 (55.86, 1,031.14) | 0.54 (0.14, 2.60) | 66.94 (17.40, 321.13) | 0.17 (0.04, 0.81) |
| **Turks and Caicos Islands** | Not Available | Not Available | Not Available | Not Available |
| **Tuvalu** | 0.00 (0.00, 0.00) | 0.00 (0.00, 0.00) | Not Available | Not Available |
| **Uganda** | 204.62 (58.18, 847.01) | 0.75 (0.21, 3.11) | 17.79 (5.06, 73.65) | 0.07 (0.02, 0.27) |
| **Ukraine** | 520.32 (135.22, 2,495.97) | 0.46 (0.12, 2.20) | 84.24 (21.89, 404.08) | 0.07 (0.02, 0.36) |
| **United Arab Emirates** | 794.07 (206.36, 3,809.16) | 0.20 (0.05, 0.97) | Not Available | Not Available |
| **United Kingdom** | Not Available | Not Available | 15,149.68 (3,937.09, 72,673.43) | 0.54 (0.14, 2.57) |
| **United States** | 114,939.84 (29,870.52, 551,369.59) | 0.64 (0.17, 3.06) | 77,538.78 (20,150.74, 371,955.68) | 0.43 (0.11, 2.07) |
| **Uruguay** | Not Available | Not Available | 173.62 (45.12, 832.88) | 0.32 (0.08, 1.54) |
| **Uzbekistan** | 664.33 (172.65, 3,186.83) | 0.98 (0.26, 4.72) | 36.11 (9.38, 173.20) | 0.05 (0.01, 0.26) |
| **Vanuatu** | 3.72 (0.97, 17.83) | 0.45 (0.12, 2.18) | Not Available | Not Available |
| **Venezuela** | 2,372.33 (616.52, 11,380.16) | 0.67 (0.17, 3.19) | Not Available | Not Available |
| **Viet Nam** | 1,498.55 (389.44, 7,188.57) | 0.82 (0.21, 3.94) | 106.90 (27.78, 512.79) | 0.06 (0.02, 0.28) |
| **Virgin Islands (U.S.)** | Not Available | Not Available | Not Available | Not Available |
| **West Bank and Gaza** | Not Available | Not Available | Not Available | Not Available |
| **Yemen** | 504.51 (134.47, 2,319.36) | 1.65 (0.44, 7.58) | 79.30 (21.14, 364.58) | 0.26 (0.07, 1.19) |
| **Zambia** | 140.50 (38.92, 606.37) | 0.58 (0.16, 2.50) | 16.86 (4.67, 72.76) | 0.07 (0.02, 0.30) |
| **Zimbabwe** | 121.70 (35.06, 493.45) | 0.90 (0.26, 3.66) | 4.43 (1.28, 17.96) | 0.03 (0.01, 0.13) |

**WEB APPENDIX TABLE 8.** Total economic losses attributable to not recommended breastfeeding practices by country and by approach.

|  | **Total future cost (health system, mortality and cognitive) attributed to not breastfeeding (EBF 0-5 months vs. non-EBF)** (US$ m). | **Total future cost (health system, mortality and cognitive) of not breastfeeding (EBF 0-5 months vs. non-EBF) as a percent (%) of GNI** | **Sensitivity analysis: Total future cost (health system, mortality and cognitive) attributable to not breastfeeding (Approach 1: BF at 6 months vs. no BF)** (US$ m). | **Sensitivity analysis: Total future cost (health system, mortality and cognitive) of not breastfeeding (Approach 1: BF at 6 months vs. no BF) as a percent (%) of GNI** |
| --- | --- | --- | --- | --- |
| **Afghanistan** | 490.99 (138.33, 2,108.26) | 2.47 (0.70, 10.59) | 321.70 (91.44, 1,377.67) | 1.62 (0.46, 6.92) |
| **Albania** | 83.44 (22.11, 397.78) | 0.68 (0.18, 3.22) | 25.11 (6.95, 117.98) | 0.20 (0.06, 0.95) |
| **Algeria** | 2,700.73 (713.57, 12,894.54) | 1.40 (0.37, 6.68) | Not Available | Not Available |
| **American Samoa** | Not Available | Not Available | Not Available | Not Available |
| **Andorra** | Not Available | Not Available | Not Available | Not Available |
| **Angola** | 4,776.31 (1,486.43, 17,427.92) | 4.57 (1.42, 16.68) | Not Available | Not Available |
| **Antigua and Barbuda** | Not Available | Not Available | Not Available | Not Available |
| **Argentina** | 4,284.27 (1,160.57, 20,276.61) | 0.79 (0.21, 3.75) | 1,661.15 (478.88, 7,693.43) | 0.31 (0.09, 1.42) |
| **Armenia** | 49.62 (14.13, 230.65) | 0.42 (0.12, 1.97) | 26.84 (8.21, 121.38) | 0.23 (0.07, 1.04) |
| **Aruba** | Not Available | Not Available | Not Available | Not Available |
| **Australia** | Not Available | Not Available | 5,078.61 (1,319.83, 24,362.21) | 0.36 (0.09, 1.71) |
| **Austria** | Not Available | Not Available | 1,531.64 (401.05, 7,331.89) | 0.38 (0.10, 1.80) |
| **Azerbaijan** | 586.99 (157.00, 2,790.29) | 0.93 (0.25, 4.41) | 374.84 (101.87, 1,772.64) | 0.59 (0.16, 2.80) |
| **Bahamas** | Not Available | Not Available | Not Available | Not Available |
| **Bahrain** | 183.22 (48.21, 875.86) | 0.67 (0.18, 3.21) | Not Available | Not Available |
| **Bangladesh** | 1,381.01 (362.88, 6,603.87) | 0.72 (0.19, 3.45) | 394.05 (106.39, 1,869.40) | 0.21 (0.06, 0.98) |
| **Barbados** | 25.15 (6.56, 120.51) | 0.61 (0.16, 2.92) | Not Available | Not Available |
| **Belarus** | 483.03 (134.78, 2,262.62) | 0.79 (0.22, 3.68) | 326.22 (94.03, 1,510.40) | 0.53 (0.15, 2.46) |
| **Belgium** | 4,096.98 (1,065.85, 19,647.50) | 0.82 (0.21, 3.93) | Not Available | Not Available |
| **Belize** | 17.72 (4.66, 84.70) | 1.10 (0.29, 5.24) | 9.94 (2.64, 47.37) | 0.62 (0.16, 2.93) |
| **Benin** | 252.25 (71.27, 1,063.94) | 2.75 (0.78, 11.61) | 168.15 (47.67, 708.49) | 1.84 (0.52, 7.73) |
| **Bermuda** | Not Available | Not Available | Not Available | Not Available |
| **Bhutan** | 12.72 (3.37, 60.65) | 0.69 (0.18, 3.28) | 3.86 (1.07, 18.14) | 0.21 (0.06, 0.98) |
| **Bolivia** | 266.86 (71.75, 1,267.12) | 0.83 (0.22, 3.94) | 122.27 (34.17, 573.51) | 0.38 (0.11, 1.78) |
| **Bosnia and Herzegovina** | 78.49 (21.42, 370.51) | 0.44 (0.12, 2.08) | Not Available | Not Available |
| **Botswana** | 257.67 (69.47, 1,200.90) | 1.76 (0.48, 8.22) | 85.90 (24.26, 394.30) | 0.59 (0.17, 2.70) |
| **Brazil** | 14,000.76 (3,745.73, 66,528.13) | 0.68 (0.18, 3.25) | 7,174.30 (1,971.68, 33,781.45) | 0.35 (0.10, 1.65) |
| **British Virgin Islands** | Not Available | Not Available | Not Available | Not Available |
| **Brunei** | Not Available | Not Available | Not Available | Not Available |
| **Bulgaria** | Not Available | Not Available | Not Available | Not Available |
| **Burkina Faso** | 199.59 (57.92, 821.04) | 1.72 (0.50, 7.09) | 88.91 (26.45, 362.92) | 0.77 (0.23, 3.13) |
| **Burundi** | 28.99 (8.91, 113.26) | 1.01 (0.31, 3.94) | 19.85 (6.24, 76.94) | 0.69 (0.22, 2.68) |
| **Cabo Verde** | 12.81 (3.37, 61.20) | 0.75 (0.20, 3.58) | Not Available | Not Available |
| **Cambodia** | 137.73 (37.04, 653.73) | 0.83 (0.22, 3.92) | 68.29 (19.00, 320.64) | 0.41 (0.11, 1.92) |
| **Cameroon** | 830.70 (247.16, 3,226.74) | 2.70 (0.80, 10.50) | 469.56 (140.30, 1,821.49) | 1.53 (0.46, 5.93) |
| **Canada** | Not Available | Not Available | 8,455.34 (2,197.37, 40,560.51) | 0.50 (0.13, 2.38) |
| **Cayman Islands** | Not Available | Not Available | Not Available | Not Available |
| **Central African Republic** | 39.29 (12.60, 137.58) | 2.40 (0.77, 8.40) | 25.39 (8.20, 88.71) | 1.55 (0.50, 5.41) |
| **Chad** | 547.09 (171.24, 1,961.44) | 4.45 (1.39, 15.94) | 329.89 (103.39, 1,182.25) | 2.68 (0.84, 9.61) |
| **Channel Islands** | Not Available | Not Available | Not Available | Not Available |
| **Chile** | Not Available | Not Available | 1,065.43 (276.88, 5,110.89) | 0.42 (0.11, 2.02) |
| **China** | 66,064.16 (17,640.97, 314,128.30) | 0.61 (0.16, 2.89) | 24,372.76 (6,806.23, 114,133.47) | 0.22 (0.06, 1.05) |
| **Colombia** | 2,200.19 (595.45, 10,421.90) | 0.64 (0.17, 3.03) | 946.42 (269.62, 4,407.52) | 0.27 (0.08, 1.28) |
| **Comoros** | 17.42 (4.70, 79.83) | 2.84 (0.77, 13.03) | 8.25 (2.25, 37.65) | 1.35 (0.37, 6.15) |
| **Congo** | 222.96 (64.09, 919.79) | 1.90 (0.55, 7.85) | 88.72 (25.93, 364.10) | 0.76 (0.22, 3.11) |
| **Costa Rica** | 187.07 (52.36, 832.14) | 0.37 (0.10, 1.66) | 36.57 (11.73, 154.70) | 0.07 (0.02, 0.31) |
| **Côte d'Ivoire** | 1,186.10 (308.69, 5,687.12) | 3.69 (0.96, 17.68) | 584.93 (152.46, 2,803.29) | 1.82 (0.47, 8.72) |
| **Croatia** | 181.08 (56.80, 648.75) | 0.34 (0.11, 1.21) | Not Available | Not Available |
| **Cuba** | 228.97 (64.33, 1,070.07) | 0.34 (0.10, 1.61) | 110.97 (33.66, 504.01) | 0.17 (0.05, 0.76) |
| **Curaçao** | Not Available | Not Available | Not Available | Not Available |
| **Cyprus** | Not Available | Not Available | Not Available | Not Available |
| **Czech Republic** | Not Available | Not Available | 572.26 (148.72, 2,745.16) | 0.30 (0.08, 1.43) |
| **Democratic People's Republic of Korea** | Not Available | Not Available | Not Available | Not Available |
| **Denmark** | Not Available | Not Available | 1,718.19 (463.82, 7,733.65) | 0.52 (0.14, 2.33) |
| **Djibouti** | Not Available | Not Available | Not Available | Not Available |
| **Dominica** | Not Available | Not Available | Not Available | Not Available |
| **Dominican Republic** | 989.20 (262.75, 4,714.21) | 1.51 (0.40, 7.18) | 582.98 (157.18, 2,765.53) | 0.89 (0.24, 4.21) |
| **Democratic Republic of the Congo** | 899.05 (237.00, 4,295.22) | 2.82 (0.74, 13.49) | 510.86 (136.12, 2,433.09) | 1.60 (0.43, 7.64) |
| **Ecuador** | 930.33 (247.43, 4,431.35) | 0.96 (0.25, 4.55) | 475.40 (129.20, 2,249.05) | 0.49 (0.13, 2.31) |
| **Egypt** | 4,452.69 (1,181.68, 21,231.22) | 1.46 (0.39, 6.94) | 1,426.44 (395.22, 6,714.23) | 0.47 (0.13, 2.20) |
| **El Salvador** | 135.91 (40.02, 547.33) | 0.56 (0.17, 2.27) | Not Available | Not Available |
| **Equatorial Guinea** | 402.31 (109.79, 1,836.50) | 3.71 (1.01, 16.95) | Not Available | Not Available |
| **Eritrea** | 27.13 (7.25, 129.07) | 1.17 (0.31, 5.58) | 12.53 (3.46, 59.03) | 0.54 (0.15, 2.55) |
| **Estonia** | Not Available | Not Available | Not Available | Not Available |
| **Ethiopia** | 853.60 (223.68, 4,084.72) | 1.45 (0.38, 6.94) | 390.89 (103.42, 1,865.05) | 0.66 (0.18, 3.17) |
| **Faroe Islands** | Not Available | Not Available | Not Available | Not Available |
| **Fiji** | 31.62 (8.33, 151.13) | 0.73 (0.19, 3.51) | Not Available | Not Available |
| **Finland** | Not Available | Not Available | 705.21 (192.83, 3,108.02) | 0.28 (0.08, 1.22) |
| **France** | Not Available | Not Available | 17,538.60 (4,557.92, 84,133.15) | 0.65 (0.17, 3.11) |
| **French Polynesia** | Not Available | Not Available | Not Available | Not Available |
| **Gabon** | 366.35 (102.86, 1,574.55) | 2.31 (0.65, 9.92) | 167.78 (47.86, 717.58) | 1.06 (0.30, 4.52) |
| **Gambia** | 20.94 (5.74, 92.25) | 2.39 (0.65, 10.51) | 9.85 (2.70, 43.36) | 1.12 (0.31, 4.94) |
| **Georgia** | 46.31 (13.76, 211.73) | 0.30 (0.09, 1.38) | Not Available | Not Available |
| **Germany** | Not Available | Not Available | 10,086.38 (2,758.00, 44,453.02) | 0.27 (0.07, 1.19) |
| **Ghana** | 594.22 (167.29, 2,595.96) | 1.47 (0.41, 6.41) | 239.12 (70.19, 1,030.94) | 0.59 (0.17, 2.55) |
| **Gibraltar** | Not Available | Not Available | Not Available | Not Available |
| **Greece** | Not Available | Not Available | 1,033.04 (268.46, 4,955.50) | 0.47 (0.12, 2.25) |
| **Greenland** | Not Available | Not Available | Not Available | Not Available |
| **Grenada** | 6.71 (1.77, 32.05) | 0.73 (0.19, 3.47) | Not Available | Not Available |
| **Guam** | Not Available | Not Available | Not Available | Not Available |
| **Guatemala** | 570.99 (166.24, 2,346.52) | 0.97 (0.28, 4.00) | 238.55 (71.72, 970.35) | 0.41 (0.12, 1.65) |
| **Guinea** | 90.01 (27.32, 347.42) | 1.51 (0.46, 5.81) | 67.01 (20.52, 257.90) | 1.12 (0.34, 4.32) |
| **Guinea-Bissau** | 29.07 (7.62, 139.08) | 2.68 (0.70, 12.80) | Not Available | Not Available |
| **Guyana** | 38.58 (10.57, 172.82) | 1.23 (0.34, 5.50) | 13.73 (3.87, 60.98) | 0.44 (0.12, 1.94) |
| **Haiti** | 161.33 (45.58, 692.04) | 1.85 (0.52, 7.95) | 97.27 (27.84, 415.57) | 1.12 (0.32, 4.78) |
| **Honduras** | 195.00 (52.36, 926.52) | 1.06 (0.28, 5.03) | 80.63 (22.64, 377.87) | 0.44 (0.12, 2.05) |
| **Hong Kong** | Not Available | Not Available | Not Available | Not Available |
| **Hungary** | Not Available | Not Available | Not Available | Not Available |
| **Iceland** | Not Available | Not Available | Not Available | Not Available |
| **India** | 14,457.81 (3,843.62, 68,902.94) | 0.69 (0.18, 3.29) | 7,592.98 (2,059.59, 35,972.16) | 0.36 (0.10, 1.72) |
| **Indonesia** | 9,353.40 (2,507.66, 44,458.56) | 1.05 (0.28, 5.01) | 3,774.66 (1,057.86, 17,697.18) | 0.43 (0.12, 2.00) |
| **Iran** | 3,156.66 (846.50, 15,006.80) | 0.62 (0.17, 2.93) | Not Available | Not Available |
| **Iraq** | 4,754.65 (1,243.19, 22,767.60) | 2.24 (0.59, 10.75) | Not Available | Not Available |
| **Ireland** | Not Available | Not Available | Not Available | Not Available |
| **Isle of Man** | Not Available | Not Available | Not Available | Not Available |
| **Israel** | Not Available | Not Available | Not Available | Not Available |
| **Italy** | Not Available | Not Available | 5,470.84 (1,421.76, 26,243.75) | 0.27 (0.07, 1.32) |
| **Jamaica** | 106.15 (28.86, 501.75) | 0.77 (0.21, 3.65) | 30.06 (9.09, 136.75) | 0.22 (0.07, 0.99) |
| **Japan** | Not Available | Not Available | 10,519.95 (2,733.92, 50,464.50) | 0.21 (0.06, 1.02) |
| **Jordan** | 529.01 (140.22, 2,523.42) | 1.49 (0.39, 7.10) | 208.39 (56.90, 985.38) | 0.59 (0.16, 2.77) |
| **Kazakhstan** | 1,370.43 (389.33, 5,964.99) | 0.69 (0.19, 2.99) | 534.70 (160.81, 2,281.74) | 0.27 (0.08, 1.14) |
| **Kenya** | 879.17 (233.63, 4,190.17) | 1.42 (0.38, 6.78) | 398.02 (108.59, 1,882.06) | 0.64 (0.18, 3.04) |
| **Kiribati** | 3.56 (0.93, 17.02) | 0.93 (0.24, 4.47) | Not Available | Not Available |
| **Kosovo** | Not Available | Not Available | Not Available | Not Available |
| **Kuwait** | 1,712.27 (451.10, 8,179.83) | 1.04 (0.27, 4.99) | Not Available | Not Available |
| **Kyrgyzstan** | 67.14 (17.96, 319.27) | 0.96 (0.26, 4.57) | 24.10 (6.77, 112.84) | 0.35 (0.10, 1.62) |
| **Laos** | 235.26 (61.68, 1,125.48) | 1.99 (0.52, 9.53) | Not Available | Not Available |
| **Latvia** | Not Available | Not Available | Not Available | Not Available |
| **Lebanon** | 385.77 (102.94, 1,835.54) | 0.86 (0.23, 4.07) | Not Available | Not Available |
| **Lesotho** | 37.89 (12.36, 129.92) | 1.38 (0.45, 4.74) | 31.88 (10.42, 109.19) | 1.16 (0.38, 3.99) |
| **Liberia** | 32.01 (9.02, 137.41) | 1.87 (0.53, 8.04) | 18.54 (5.29, 79.27) | 1.08 (0.31, 4.64) |
| **Libya** | Not Available | Not Available | Not Available | Not Available |
| **Liechtenstein** | Not Available | Not Available | Not Available | Not Available |
| **Lithuania** | Not Available | Not Available | Not Available | Not Available |
| **Luxembourg** | Not Available | Not Available | Not Available | Not Available |
| **Macao SAR, China** | Not Available | Not Available | Not Available | Not Available |
| **Madagascar** | 177.79 (47.30, 847.22) | 1.76 (0.47, 8.40) | 55.46 (15.50, 260.37) | 0.55 (0.15, 2.58) |
| **Malawi** | 96.45 (26.69, 454.29) | 1.64 (0.45, 7.73) | 54.73 (15.85, 254.14) | 0.93 (0.27, 4.32) |
| **Malaysia** | 1,662.14 (452.71, 7,462.10) | 0.52 (0.14, 2.33) | Not Available | Not Available |
| **Maldives** | 18.99 (5.03, 90.60) | 0.67 (0.18, 3.18) | 3.32 (0.96, 15.41) | 0.12 (0.03, 0.54) |
| **Mali** | 503.08 (131.65, 2,408.53) | 3.77 (0.99, 18.03) | 293.13 (77.09, 1,401.41) | 2.19 (0.58, 10.49) |
| **Malta** | Not Available | Not Available | Not Available | Not Available |
| **Marshall Islands** | 3.36 (0.90, 16.00) | 1.33 (0.36, 6.34) | 4.36 (1.16, 20.80) | 1.73 (0.46, 8.23) |
| **Mauritania** | 126.43 (34.84, 555.92) | 2.32 (0.64, 10.21) | 70.74 (19.62, 310.50) | 1.30 (0.36, 5.70) |
| **Mauritius** | 59.77 (16.87, 269.52) | 0.48 (0.14, 2.18) | Not Available | Not Available |
| **Mexico** | 8,245.72 (2,206.13, 39,199.29) | 0.67 (0.18, 3.18) | Not Available | Not Available |
| **Micronesia** | 9.39 (2.44, 45.07) | 2.53 (0.66, 12.12) | Not Available | Not Available |
| **Moldova** | Not Available | Not Available | 13.57 (3.53, 65.08) | 0.17 (0.04, 0.82) |
| **Monaco** | Not Available | Not Available | Not Available | Not Available |
| **Mongolia** | 67.08 (18.81, 293.35) | 0.59 (0.16, 2.56) | Not Available | Not Available |
| **Montenegro** | 27.16 (7.36, 128.49) | 0.60 (0.16, 2.86) | Not Available | Not Available |
| **Morocco** | 1,246.85 (329.12, 5,953.28) | 1.18 (0.31, 5.61) | 504.35 (136.16, 2,391.50) | 0.48 (0.13, 2.26) |
| **Mozambique** | 436.76 (115.05, 2,086.91) | 2.66 (0.70, 12.69) | 218.12 (58.23, 1,038.06) | 1.33 (0.35, 6.31) |
| **Myanmar** | 430.71 (128.73, 1,670.67) | 0.69 (0.21, 2.68) | Not Available | Not Available |
| **Namibia** | 237.15 (63.21, 1,129.44) | 1.86 (0.49, 8.84) | 164.58 (44.34, 781.28) | 1.29 (0.35, 6.12) |
| **Nauru** | 0.24 (0.06, 1.11) | 0.15 (0.04, 0.71) | Not Available | Not Available |
| **Nepal** | 104.78 (29.78, 456.49) | 0.50 (0.14, 2.18) | 27.65 (8.69, 116.54) | 0.13 (0.04, 0.56) |
| **Netherlands** | Not Available | Not Available | 4,383.88 (1,139.28, 21,029.61) | 0.53 (0.14, 2.54) |
| **New Caledonia** | Not Available | Not Available | Not Available | Not Available |
| **New Zealand** | Not Available | Not Available | 596.00 (154.89, 2,859.05) | 0.32 (0.08, 1.55) |
| **Nicaragua** | 127.98 (33.94, 610.29) | 1.08 (0.29, 5.16) | 61.02 (16.54, 289.10) | 0.52 (0.14, 2.45) |
| **Niger** | 260.70 (68.91, 1,244.64) | 3.33 (0.88, 15.89) | 207.84 (55.17, 991.05) | 2.65 (0.70, 12.66) |
| **Nigeria** | 21,057.16 (5,776.41, 92,714.71) | 4.10 (1.12, 18.04) | 12,454.63 (3,424.15, 54,801.36) | 2.42 (0.67, 10.66) |
| **Northern Mariana Islands** | Not Available | Not Available | Not Available | Not Available |
| **Norway** | Not Available | Not Available | 957.74 (265.02, 4,206.29) | 0.20 (0.05, 0.86) |
| **Oman** | 615.12 (162.86, 2,935.11) | 0.81 (0.21, 3.87) | 770.56 (203.26, 3,680.72) | 1.01 (0.27, 4.85) |
| **Pakistan** | 5,819.98 (1,552.70, 27,709.51) | 2.14 (0.57, 10.20) | 3,476.31 (943.62, 16,466.86) | 1.28 (0.35, 6.06) |
| **Palau** | Not Available | Not Available | Not Available | Not Available |
| **Panama** | 470.80 (131.09, 2,063.32) | 1.01 (0.28, 4.42) | Not Available | Not Available |
| **Papua New Guinea** | 223.98 (58.85, 1,070.95) | 1.34 (0.35, 6.40) | Not Available | Not Available |
| **Paraguay** | 305.55 (84.54, 1,364.73) | 1.10 (0.30, 4.91) | 153.27 (43.43, 679.30) | 0.55 (0.16, 2.44) |
| **Peru** | 646.71 (175.43, 3,061.45) | 0.34 (0.09, 1.59) | 267.03 (76.76, 1,240.16) | 0.14 (0.04, 0.64) |
| **Philippines** | 3,757.41 (997.19, 17,908.58) | 1.05 (0.28, 5.01) | 2,220.19 (597.70, 10,534.51) | 0.62 (0.17, 2.95) |
| **Poland** | Not Available | Not Available | Not Available | Not Available |
| **Portugal** | Not Available | Not Available | Not Available | Not Available |
| **Puerto Rico** | Not Available | Not Available | Not Available | Not Available |
| **Qatar** | 417.36 (113.12, 1,976.06) | 0.22 (0.06, 1.05) | Not Available | Not Available |
| **Republic of Korea** | Not Available | Not Available | 3,671.90 (954.25, 17,614.19) | 0.26 (0.07, 1.27) |
| **Romania** | 601.63 (158.28, 2,876.18) | 0.32 (0.08, 1.53) | Not Available | Not Available |
| **Russia** | Not Available | Not Available | Not Available | Not Available |
| **Rwanda** | 35.00 (9.47, 165.92) | 0.43 (0.12, 2.05) | 17.59 (4.95, 82.39) | 0.22 (0.06, 1.02) |
| **Saint Kitts and Nevis** | Not Available | Not Available | Not Available | Not Available |
| **Saint Lucia** | Not Available | Not Available | Not Available | Not Available |
| **Saint Martin** | Not Available | Not Available | Not Available | Not Available |
| **Saint Vincent and the Grenadines** | Not Available | Not Available | Not Available | Not Available |
| **Samoa** | 5.93 (1.62, 27.06) | 0.78 (0.21, 3.56) | Not Available | Not Available |
| **San Marino** | Not Available | Not Available | Not Available | Not Available |
| **Sao Tome and Principe** | 3.54 (0.96, 16.75) | 1.05 (0.29, 4.99) | 1.74 (0.49, 8.11) | 0.52 (0.15, 2.42) |
| **Saudi Arabia** | Not Available | Not Available | Not Available | Not Available |
| **Senegal** | 351.68 (92.62, 1,680.61) | 2.36 (0.62, 11.30) | 117.89 (31.86, 559.09) | 0.79 (0.21, 3.76) |
| **Serbia** | 289.15 (80.47, 1,354.75) | 0.74 (0.20, 3.45) | Not Available | Not Available |
| **Seychelles** | Not Available | Not Available | Not Available | Not Available |
| **Sierra Leone** | 135.92 (35.93, 648.85) | 3.39 (0.90, 16.18) | 84.78 (22.64, 403.55) | 2.11 (0.56, 10.06) |
| **Singapore** | Not Available | Not Available | 629.44 (199.37, 2,213.18) | 0.22 (0.07, 0.77) |
| **Sint Maarten (Dutch part)** | Not Available | Not Available | Not Available | Not Available |
| **Slovakia** | Not Available | Not Available | Not Available | Not Available |
| **Slovenia** | Not Available | Not Available | Not Available | Not Available |
| **Solomon Islands** | 8.47 (2.26, 40.34) | 0.75 (0.20, 3.59) | Not Available | Not Available |
| **Somalia** | Not Available | Not Available | Not Available | Not Available |
| **South Africa** | 2,282.10 (701.71, 8,771.28) | 0.68 (0.21, 2.62) | 1,749.01 (543.98, 6,696.96) | 0.52 (0.16, 2.00) |
| **South Sudan** | 306.30 (80.79, 1,463.21) | 3.16 (0.83, 15.08) | Not Available | Not Available |
| **Spain** | Not Available | Not Available | 2,989.76 (884.61, 11,633.68) | 0.23 (0.07, 0.88) |
| **Sri Lanka** | 202.97 (53.62, 968.93) | 0.25 (0.07, 1.22) | 119.11 (31.83, 566.63) | 0.15 (0.04, 0.71) |
| **Sudan** | 1,325.20 (395.53, 5,403.47) | 1.72 (0.51, 6.99) | 779.02 (240.23, 3,142.54) | 1.01 (0.31, 4.07) |
| **Suriname** | 58.33 (15.75, 267.02) | 1.15 (0.31, 5.25) | 24.51 (6.74, 111.52) | 0.48 (0.13, 2.19) |
| **Swaziland** | 73.90 (19.83, 351.18) | 1.75 (0.47, 8.31) | 59.33 (16.05, 281.26) | 1.40 (0.38, 6.65) |
| **Sweden** | Not Available | Not Available | 1,241.73 (409.04, 4,178.99) | 0.22 (0.07, 0.74) |
| **Switzerland** | Not Available | Not Available | 2,188.43 (568.73, 10,497.97) | 0.31 (0.08, 1.50) |
| **Syria** | 345.94 (91.24, 1,652.55) | 0.96 (0.25, 4.57) | Not Available | Not Available |
| **Taiwan** | Not Available | Not Available | Not Available | Not Available |
| **Tajikistan** | 219.15 (57.94, 1,046.11) | 2.02 (0.53, 9.65) | 95.43 (25.79, 452.62) | 0.88 (0.24, 4.18) |
| **Tanzania** | 878.61 (231.95, 4,195.26) | 1.84 (0.49, 8.80) | 425.91 (114.30, 2,023.62) | 0.89 (0.24, 4.24) |
| **Thailand** | 2,232.98 (614.21, 10,328.43) | 0.57 (0.16, 2.66) | 713.99 (214.46, 3,195.46) | 0.18 (0.06, 0.82) |
| **The former Yugoslav Republic of Macedonia** | 56.14 (15.22, 265.37) | 0.53 (0.14, 2.49) | 23.22 (6.67, 107.45) | 0.22 (0.06, 1.01) |
| **Timor-Leste** | 58.04 (15.17, 277.96) | 2.14 (0.56, 10.26) | 33.24 (8.72, 159.01) | 1.23 (0.32, 5.87) |
| **Togo** | 64.49 (18.45, 270.98) | 1.62 (0.46, 6.82) | 34.47 (10.03, 144.11) | 0.87 (0.25, 3.63) |
| **Tonga** | 3.91 (1.05, 18.53) | 0.86 (0.23, 4.08) | Not Available | Not Available |
| **Trinidad and Tobago** | 204.01 (55.08, 966.27) | 0.85 (0.23, 4.03) | 101.65 (28.48, 475.23) | 0.42 (0.12, 1.98) |
| **Tunisia** | 520.24 (137.30, 2,484.07) | 1.18 (0.31, 5.62) | 150.81 (41.29, 711.94) | 0.34 (0.09, 1.61) |
| **Turkey** | 6,077.01 (1,624.20, 28,910.98) | 0.78 (0.21, 3.69) | 2,212.81 (619.97, 10,374.28) | 0.28 (0.08, 1.33) |
| **Turkmenistan** | 449.59 (118.84, 2,145.50) | 1.13 (0.30, 5.41) | 301.58 (80.37, 1,435.49) | 0.76 (0.20, 3.62) |
| **Turks and Caicos Islands** | Not Available | Not Available | Not Available | Not Available |
| **Tuvalu** | 0.12 (0.03, 0.52) | 0.19 (0.05, 0.83) | Not Available | Not Available |
| **Uganda** | 429.78 (128.02, 1,753.47) | 1.58 (0.47, 6.43) | 242.96 (74.91, 980.11) | 0.89 (0.27, 3.60) |
| **Ukraine** | 574.51 (164.08, 2,667.58) | 0.51 (0.14, 2.36) | 138.43 (50.75, 575.70) | 0.12 (0.04, 0.51) |
| **United Arab Emirates** | 838.92 (228.90, 3,964.89) | 0.21 (0.06, 1.00) | Not Available | Not Available |
| **United Kingdom** | Not Available | Not Available | 15,149.68 (3,937.09, 72,673.43) | 0.54 (0.14, 2.57) |
| **United States** | 114,968.11 (29,898.79, 551,397.86) | 0.64 (0.17, 3.06) | 77,567.05 (20,179.02, 371,983.95) | 0.43 (0.11, 2.07) |
| **Uruguay** | Not Available | Not Available | 173.62 (45.12, 832.88) | 0.32 (0.08, 1.54) |
| **Uzbekistan** | 936.64 (246.65, 4,475.57) | 1.39 (0.37, 6.63) | 308.41 (83.38, 1,461.94) | 0.46 (0.12, 2.17) |
| **Vanuatu** | 6.62 (1.77, 31.43) | 0.81 (0.22, 3.84) | Not Available | Not Available |
| **Venezuela** | 2,441.24 (685.42, 11,449.06) | 0.68 (0.19, 3.21) | Not Available | Not Available |
| **Viet Nam** | 1,828.41 (483.20, 8,722.05) | 1.00 (0.26, 4.78) | 436.76 (121.54, 2,046.27) | 0.24 (0.07, 1.12) |
| **Virgin Islands (U.S.)** | Not Available | Not Available | Not Available | Not Available |
| **West Bank and Gaza** | Not Available | Not Available | Not Available | Not Available |
| **Yemen** | 713.49 (194.96, 3,256.38) | 2.33 (0.64, 10.65) | 288.28 (81.63, 1,301.61) | 0.94 (0.27, 4.26) |
| **Zambia** | 350.68 (101.19, 1,494.68) | 1.45 (0.42, 6.17) | 227.04 (66.94, 961.07) | 0.94 (0.28, 3.97) |
| **Zimbabwe** | 263.37 (75.97, 1,067.43) | 1.95 (0.56, 7.92) | 146.10 (42.19, 591.94) | 1.08 (0.31, 4.39) |
